# Sports Pre‐Participation Evaluation in Healthy Adults—A Survey‐Based Assessment of Guideline Acceptability and Feasibility Among Sports Physicians and Participants

**DOI:** 10.1002/gin2.70032

**Published:** 2025-08-20

**Authors:** Käthe Goossen, Alina Weise, Anja Hirschmüller, Christine Joisten, Sandra Jaax, Charlotte Kreutz, Nadja Koensgen, Jessica Breuing

**Affiliations:** ^1^ Institute for Research in Operative Medicine (IFOM) Witten/Herdecke University Cologne Germany; ^2^ Departement of Orthopedic Surgery and Traumatology, Medical Center Albert‐Ludwigs‐University of Freiburg Freiburg Germany; ^3^ Department for Physical Activity in Public Health, Institute of Movement and Neurosciences German Sport University Cologne Cologne Germany; ^4^ German Federation of Sports Medicine and Prevention Frankfurt am Main Germany

**Keywords:** acceptability, feasibility, guideline, patient involvement, sports pre‐participation evaluation, survey

## Abstract

**Introduction:**

Sports pre‐participation evaluations (PPEs) aim to identify people at increased risk of acute adverse events during physical activity. Little is known about their acceptability and feasibility among physicians and participants (‘interest holders’). During the development of a consensus‐based PPE guideline in Germany targeted at healthy adults who plan to take up or resume intense physical activity, we used surveys to assess interest holder perceptions of the acceptability and feasibility of the PPE, and to obtain their feedback on draft guideline recommendations.

**Methods:**

Cross‐sectional online surveys were conducted separately in two interest holder groups. Acceptability was evaluated among adults (≥ 18 years) in Germany (‘consumers’), the target population of the PPE guideline. Feasibility was investigated among potential guideline users, which included sports medicine specialists and general practitioners practising in Germany (‘sports physicians’). Agreement with nine acceptability and feasibility statements was analysed descriptively. Comments on draft recommendations were collected and grouped thematically.

**Results:**

A total of 144 consumers and 204 physicians (186 with certification in sports medicine) completed the survey. Among the consumers, 82% would consider making use of a PPE, 8% were neutral, and 10% would not. Only 1% had ‘concerns or reservations’. The main barrier was a potential non‐reimbursement of the PPE by their health insurance (61%). Of the 186 certified sports physicians, 83% would offer or already offer a PPE, 12% were neutral, and 5% would not offer a PPE. Potential barriers were non‐reimbursement by health insurers (20%), the required time and effort (13%), and qualification issues (14%). About half of the draft recommendations (11/23, 48%) were modified based on interest holder feedback.

**Discussion:**

Conducting consumer and end‐user surveys as part of the guideline development process increased awareness of the main acceptability and feasibility issues, provided relevant suggestions to the draft recommendations, and identified considerations for guideline implementation.

## Summary

What is already known on this topic?

- Interest holder involvement is recommended during guideline development to ensure that the guideline is feasible and acceptable, and to facilitate its uptake.
- Approaches to involving patients and the public, as described in the GIN Public Toolkit, include consultation (e.g., surveys, focus groups or interviews) and participation (e.g., patient representatives on guideline panels).

What this study adds to guideline development?

- Surveys were a useful alternative to recruiting consumer representatives to the guideline panel in a context where no consumer organisation existed and the consumer group was heterogeneous.
- Parallel surveys allowed consultation with multiple interest holder groups on feasibility and acceptability as well as on draft recommendations.
- Consulting interest holders before finalising and voting on the recommendations helped the guideline group to include interest holder views in the guideline.

**Abbreviations**
BMI: body mass index
CROSS: Consensus‐Based Checklist for Reporting of Survey Studies
DGSP: German Federation of Sports Medicine and Prevention (*Deutsche Gesellschaft für Sportmedizin und Prävention e.V.)*
PPE: pre‐participation evaluation
WHO: World Health Organization

## 1 Introduction

Regular physical activity is positively associated with healthy life expectancy and well‐being [1, 2, 3, 4]. Population‐based studies have demonstrated the beneficial effects of exercise on physical health [1], mental well‐being [5, 6], healthy ageing [2, 7], and prolonged life expectancy [1, 4, 8]. However, sedentary behaviour is widespread, particularly in high‐income countries [9]. At an individual level, the benefits of physical activity greatly outweigh the risks, but in rare cases serious musculoskeletal injuries or cardiovascular adverse events may occur [10, 11, 12, 13, 14, 15]. The risk is higher in men, in older people with pre‐existing health problems, functional limitations, or sedentary lifestyle, and in the context of intensive exercise [16, 17, 18, 19, 20].

Sports pre‐participation evaluations (PPE) aim to identify risk factors and medical conditions that predispose individuals to exercise‐induced adverse events [21, 22, 23, 24]. A PPE thereby promotes the safe initiation of physical activity, its resumption after an extended pause, or its regular conduct [25]. It generally includes a medical history and a physical examination. The choice of specific diagnostic tests is the subject of international debate [26]. A lack of robust evidence on the impact of a PPE on patient‐relevant outcomes contributes to this controversy [14, 25, 27, 28]. Therefore, guidelines in this area rely mainly on consensus and clinical expertise [28].

Guideline development should involve all relevant interested groups [29, 30, 31, 32], including clinical experts, the target population, and guideline users [33, 34] (henceforth referred to as ‘interest holders’ [35, 36]). Interest holder involvement helps to ensure that a guideline is feasible and acceptable [37], and facilitates guideline uptake [38].

The German Federation of Sports Medicine and Prevention (DGSP) has recently developed a consensus‐based guideline on the PPE for healthy adults [39]. The guideline is targeted at a population of adults, including individuals recovering from conditions such as cancer, joint injuries or joint surgeries, who appear healthy and plan to take up or resume intense physical activity. It is not intended for people with chronic diseases, such as cardiovascular disease or diabetes mellitus, due to the specific risks in this population and their need for adjusted PPE [40, 41]. It complements existing PPE guidance, for example, for young [42, 43, 44, 45, 46, 47] or elite athletes [22], athletes with cardiovascular disease [23, 48, 49, 50] and pregnant/postpartum women [51, 52].

**Figure 1:**
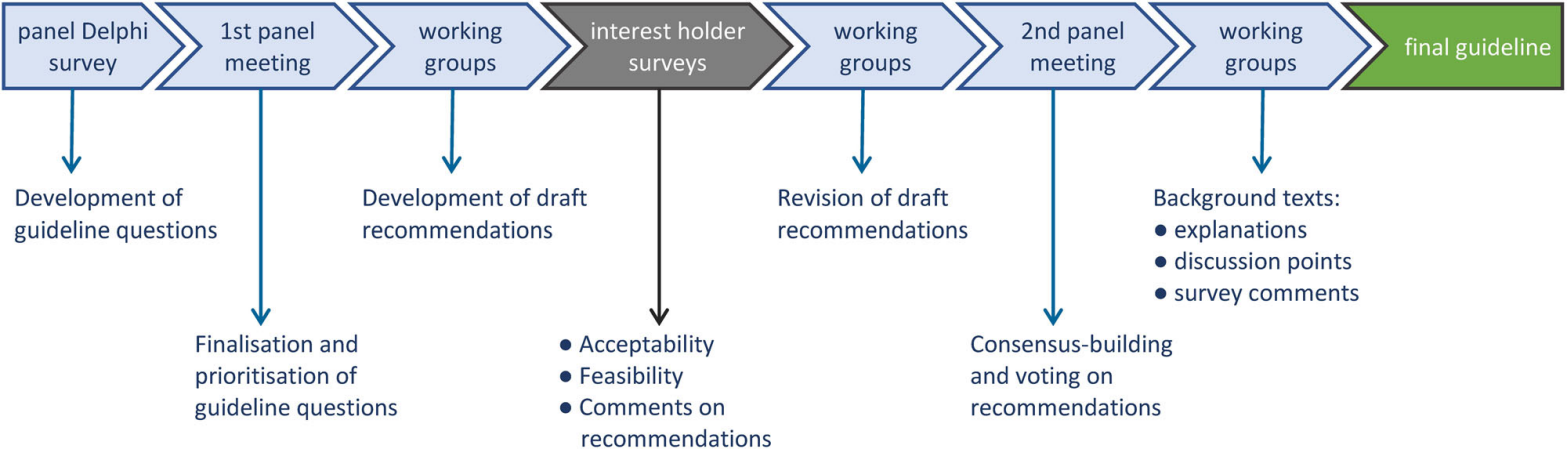
Development process for the German consensus‐based guideline on the PPE for healthy adults.

The guideline provides recommendations on the indication for a PPE, its components and an algorithm for test selection. The guideline development process followed the Guidance Manual and Rules for Guideline Development by the Association of the Scientific Medical Societies in Germany for consensus‐based guidelines [53]. Due to the lack of evidence from comparative studies for most of the clinical questions in the PPE guideline [25], the DGSP did not consider the development of an evidence‐based guideline to be feasible or resource‐efficient, and decided to develop the guideline based on consensus. The process involved the development of guideline questions by an interdisciplinary guideline panel composed of delegates from 16 healthcare and sports organisations, the development of recommendations in working groups of clinical and methodology experts, and a structured consensus conference to discuss, finalise and vote on each recommendation (Figure 1).

To achieve broad consensus among clinical experts, users and potential participants during guideline development, we added a step ‘interest holder surveys’ to this process after draft recommendations were available. We conducted online surveys among sports physicians and sedentary or physically active adults in Germany (‘consumers’) and used the results to revise draft recommendations and to inform discussions in the guideline panel. A broad consensus seemed particularly important in this area, where (1) evidence of benefits and harms is weak or lacking [38] and (2) no appropriate consumer organisation could be identified that could have nominated a member to represent the diverse target population on the guideline panel.

The objectives of this survey study were to assess the feasibility and acceptability of the PPE, to obtain interest holder feedback on draft recommendations, and to adapt the recommendations accordingly.

## 2 Methods

This study is reported using the Consensus‐based Checklist for Reporting of Survey Studies (CROSS) where applicable [54].

### 2.1 Definitions

For the purpose of this study, we defined acceptability as the extent to which consumers consider a PPE to be appropriate based on anticipated cognitive and emotional responses to the examination, with seven component constructs: affective attitude, burden, test coherence, perceived test effectiveness, self‐efficacy, financial opportunity costs and ethicality [55, 56]. Feasibility was defined as the extent to which sports physicians perceive that they have the skills, resources and equipment to administer and use a PPE in their daily practice. We adapted the same seven component constructs as for acceptability [55, 56].

### 2.2 Study Design

This study consisted of two cross‐sectional online surveys, one of consumers and one of sports physicians. The study was approved by the Ethics Committee of the University of Freiburg, Germany (24‐1249‐S1). We collected the primary data in October 2023. At that time in the guideline development process, draft consensus‐based recommendations were available and could be included in the surveys (see Figure 1), and there was still an opportunity to revise these recommendations based on the survey results.

### 2.3 Study Population

#### 2.3.1 Consumer Survey

Eligible participants were adults (≥ 18 years) resident in Germany. They could be physically inactive or active at any level. Individuals with insufficient German language skills to complete the survey were excluded. This survey used single‐stage convenience sampling. The target sample size was *N* = 150.

#### 2.3.2 Sports Physicians' Survey

Eligible participants were physicians certified in sports medicine or general medicine practising in Germany. In Germany, formal qualification in sports medicine is based on an interdisciplinary continuing education curriculum, which is completed in addition to specialist medical training, for example, in internal medicine or orthopaedics. The theoretical part (240 h) and covers sports medicine knowledge drawn from various medical specialties, including internal medicine, orthopaedics, neurology, psychiatry, nutritional medicine and pharmacology. It is supplemented by the sport‐specific application of sports medicine expertise and practical training (120 h) involving supervised sports medical activity in real‐life settings.

This survey used single‐stage list‐based sampling. The target sample size was *N* = 100. Target sample sizes were not calculated based on formal methods, but chosen as trade‐offs between adequate representation (i.e., capturing as many different perspectives as possible) and available resources (the feasibility of qualitatively evaluating free‐text comments in the 3‐week period imposed by the guideline processes) [57].

### 2.4 Survey Structure and Content

We used two surveys for the two different interest holder groups. The acceptability survey was administered to consumers, and the feasibility survey was administered to sports physicians. We developed our own surveys, because we could not identify any published, validated questionnaire to assess interest holders' perceived acceptability and feasibility of preventive screening examinations. Based on internationally agreed criteria for measuring the acceptability of diagnostic tests [55], one guideline methodologist proposed one or two statements for each item. Each statement was then refined by the methodology team and finalised by consensus within the guideline steering group. Table 1 shows the final survey statements. Participants were asked to rate their agreement with each statement on 5‐point Likert scales. The final questionnaires are provided in Supporting Information S2 and S3: Supporting Files 2 and 3.

**Table 1:** Domains proposed for the acceptability of diagnostic tests [55] and survey statements.^a^

Affective attitude
How the patient feels about the test. A global measure of acceptability.
*Consumer statement*: I would have a PPE done.
*Sports physician statement*: I would offer PPEs or I am already doing so.
Burden
The perceived amount of effort required to perform the test and any resulting side‐effects, both physical and psychological.
*Consumer statements*: I would accept the burden involved in a PPE (e.g., blood sampling, emotional stress caused by test results). / The effort associated with the PPE would be feasible for me (travelling, time required, other).
*Sports physician statement*:^b^ The time and effort involved in PPEs would be feasible in my practice or clinic.
Test coherence
The extent to which the patient understands why the test is being done given their symptoms and context.
*Consumer statement*: I understand the purpose of a PPE.
*Sports physician statement*: I understand why PPEs would be useful for my patients.
Perceived test effectiveness^c^
The extent to which the patient believes that the test is likely to achieve its purpose given their symptoms and context, and give an accurate result.
*Consumer statements*: A PPE would help to diagnose or rule out health conditions relevant to exercising./A PPE would help me to choose the most appropriate form and intensity of physical activity.
*Sports physician statements*: PPEs would help me to make correct diagnoses./PPEs would help me to advise patients on the correct mode, amount, and intensity of physical activity.
Self‐efficacy
The patient's confidence that they can complete the test
*Consumer statement*: I would be able to take the individual tests of the PPE without any problems.
*Sports physician statements*:^d^ I feel qualified to carry out the internal medicine part of a PPE./I feel qualified to carry out the orthopaedic part of a PPE.
Financial opportunity costs
The extent to which there are costs associated with having/doing the test.
*Consumer statement*: I would only go for a PPE if it is reimbursed by the health insurance company.
*Sports physician statement*: I could only offer PPEs if they are reimbursed by statutory or private health insurance.
Ethicality
The extent to which the test is a good fit with the patient's ideological, religious or political beliefs and preferences.
*Consumer statement*: I would have concerns or reservations about having a PPE done.
*Sports physician statement*: I would have concerns or reservations about offering PPEs.

Abbreviation: PPE = pre‐participation evaluation.

^a^ Questions translated from the German survey text by the authors.

^b^ For the feasibility survey, we asked about resources instead of burden.

^c^ Test effectiveness was surveyed in the context of the goals defined for the PPE by the DGSP, which were to make correct diagnoses and to advise patients on the correct form, amount and intensity of physical activity.

^d^ For the feasibility survey, we asked about qualification instead of self‐efficacy.

#### 2.4.1 Consumer Survey

The survey was structured into three parts. Part 1 contained sociodemographic and individual characteristics including age, gender, height, weight, statutory or private health insurance, chronic illness, current level and goal of physical activity, and previous experience with the PPE (10 questions). Part 2 contained nine questions on the acceptability of the PPE and a free‐text field for comments (Table 1). In Part 3, participants were asked to comment on 23 draft recommendations. For each of the draft recommendations, we provided two alternatives. In the online survey, we used explanatory videos about the survey and about the PPE, and mouse‐over tooltips to explain technical language.

#### 2.4.2 Sports Physicians' Survey

The survey was structured into three parts. In Part 1, we recorded sociodemographic and professional characteristics including age, gender, specialty, sector of care (outpatient or inpatient), setting of practice, employment status, certification in sports medicine and previous experience with the PPE in elite and recreational athletes (11 questions). In Part 2, participants were asked to comment on the same 23 draft recommendations. Part 3 contained nine questions on the feasibility of the PPE and a free‐text field for comments (Table 1).

### 2.5 Survey Preparation and Administration

We hosted the survey on LimeSurvey Community Edition (Version 5.6.8) [58]. We pre‐tested the online surveys by asking two recreationally active consumers (one female aged > 60, one male aged < 60) and two sports physicians (one academic hospital‐based sports physician specialised in orthopaedics, and one non‐academic private practice‐based sports physician specialised in general medicine) to complete the survey in the presence of one researcher. These pre‐tests were performed according to the think‐aloud method [59]. The researcher recorded any remarks on the content and comprehensibility of the surveys. We then adapted the surveys according to the comments received. In the pre‐tests, the participants completed all items of the survey and there were no missing data.

#### 2.5.1 Consumer Survey Recruitment

Following ethical approval and completion of the pre‐tests, we invited participants through digital flyers. These contained information on the aims, scope, and content of the research project as well as a link and QR code to the online survey. We contacted the organisations listed in Supporting Information S1: Appendix 1 for recruitment, providing digital pictures and video versions of the flyer. Participants were not screened as this was an anonymous online survey. The survey was initially open for 2 weeks. Due to slow recruitment, this period was extended to 24 days, from 6 to 30 October 2023.

#### 2.5.2 Sports Physicians' Survey Recruitment

Sports physicians were invited to the survey via the email newsletter of the DGSP. In addition, the survey was distributed via the list server general medicine (‘Listserv Allgemeinmedizin’, https://www.degam.de/im-dialog) of the German Society of General Practice and Family Medicine, which reaches more than 1000 general practitioners. We sent out the initial invitation on 4 October 2023 and followed it up with a reminder email on 11 October 2023. The survey was closed after 2 weeks, on 18 October 2023.

### 2.6 Ethical Considerations

The Ethics Committee of the University of Freiburg approved the study (Reference Number 23‐1369‐S2). Responders' identity remained anonymous, and confidentiality was maintained.

### 2.7 Data Analysis

We exported the results for all completed surveys from LimeSurvey into MS Excel for data processing and analysis. Missing data were not imputed.

We used descriptive statistics to present sociodemographic, personal and professional characteristics of respondents. The percentages calculated for all participant characteristics used the number of respondents as the denominator, and the percentage of missing data was reported for each item.

The primary outcomes were the acceptability (consumer survey) and feasibility of the PPE (sports physicians' survey). We categorised the acceptability and feasibility responses for each statement (see Table 1) into agreement (values of 4 and 5 on the Likert scale), neutral (value of 3) or disagreement (values of 1 and 2) and calculated percentages for each.

A formal qualitative content analysis of free‐text comments was not performed due to the large number of comments and short time frame available for processing. Instead, we grouped all free‐text comments on draft recommendations by thematic category. One researcher derived thematic categories from the comments using an inductive approach. The categories were discussed within the research team until consensus was reached. We narratively present results in thematic categories and provide illustrative quotes based on participants' comments translated from German. A complete list of thematic categories and themes is provided in Supporting Information S1: Supporting File 1.

### 2.8 Use of Interest Holder Survey Results to Change Recommendations

We discussed the aggregated comments with the guideline panel, who made changes to recommendations where they considered it appropriate. The panel subsequently discussed and voted on modified recommendations in a consensus conference.

In addition, we counted recommendations that were modified following feedback by consumers and/or sports physicians and present the results using descriptive statistics.

### 2.9 Deviations From the Protocol

Pre‐specified inclusion criteria for the sports physicians' survey had limited eligibility of participants to physicians with certification in sports medicine. When finalising the survey design, we made the decision to also include general practitioners and included a filter question by certification in sports medicine (yes/no). The reason was that this might provide more comprehensive information of the actual care provision in Germany.

Having observed in the consumer survey that some groups were underrepresented compared to the general population, we conducted an exploratory multivariable binary logistic regression of the target group of the guideline, that is, a subpopulation of adults who were sedentary or recreationally active but not competitive athletes. The aim was to assess whether the perspective on the acceptability of the PPE of underrepresented groups within the sample differed significantly compared to the rest of the sample. Details are presented in Supporting Information S1: Appendix 2.

## 3 Results

### 3.1 Recruitment and Respondent Characteristics

#### 3.1.1 Consumer Survey

One organisation included a link to the survey on their website, two in their newsletters. Other organisations we contacted did not reply or did not participate (see Supporting Information S1: Appendix 1). A total of 145 people completed the survey. One participant was excluded due to age (< 18 years). With 144 eligible participants, the recruitment target (*N* = 150) was almost reached. We could not calculate the response rate because the number of people who received the survey invitation via social media was unknown.

The sample analysed included relevant groups in terms of age, gender, physical activity and chronic disease (Table 2). Compared to the general population in Germany, older (≥ 60) and sedentary people, people with obesity, musculoskeletal or metabolic conditions, and men were underrepresented in the sample.

**Table 2:** Sociodemographic and other personal characteristics of respondents to the consumer survey (*N* = 144, with population rates for Germany for comparison, where available), and sociodemographic and professional characteristics of respondents to the sports physicians' survey (*N* = 204).

	Rates in the sample	Population rates in Germany (literature data)
Consumer survey		
Age^a^	58.3% aged 18–39	29.4% of adults aged 20–40
	27.8% aged 40–59	32.8% of adults aged 40–60
	11.8% aged ≥ 60	35.4% of adults aged ≥ 60 [60]
	2.1% n.r.	
Gender^a^	31.3% male	49.3% male
	65.3% female	50.7% female [61]
	1.4% diverse	
	0.7% non‐binary	
	1.4% n.r.	
BMI^a^^,^ b	3.5% underweight (BMI < 18.5)	2.0% underweight (BMI < 18.5)
	86.1% normal/overweight (BMI 18.5–30)	81.2% normal/overweight (BMI 18.5–30)
	9.0% obese (BMI ≥ 30)	16.8% obese (BMI ≥ 30) [62]
	1.4% n.r.	
Health insurance^a^	84.0% statutory	88% statutory
	14.6% private	10% private [63]
	1.4% n.r.	
Chronically ill	22.9% overall	in women/men aged < 50 [64]:
	4.9% musculoskeletal disease	22.4/15.8% musculoskeletal disease
	4.9% respiratory disease	7.1/5.2% respiratory disease
	3.5% cardiovascular disease	1.7/2.2% cardiovascular disease
	2.8% metabolic disease	21.2/24.9% cardiometabolic disease^c^
	6.9% other disease	
	1.4% n.r.	
Level of physical activity^a^^,^ d	20.1% rarely/never active	in women/men [65]:
	34.0% recreationally active	57.4%/52.0% rarely/never active
	36.8% trained	
	6.9% highly trained	
	0% elite	
	1.4% n.r.	
Goal of physical activity^a^	23.6% maintain level of activity	
	15.3% take up or return to sport	
	20.8% be active more often	
	18.8% be active at higher intensity	
	18.8% participate in competitions	
	1.4% n.r.	
Sports physicians' survey		
Age^a^	9.8% aged 18–39	
	48.5% aged 40–59	
	34.3% aged ≥ 60	
	7.4% n.r.	
Gender^a^	67.6% male	
	29.1% female	
	0% diverse	
	0% non‐binary	
	3.3% n.r.	
Area of specialty^e^	32% general medicine	
	26% internal medicine	
	22% orthopaedics/traumatology	
	16% cardiology	
	5% surgery	
	2% paediatrics	
	14% other	
	2% n.r.	
Sector^e^	80% outpatient	
	14% inpatient	
	6% n.r.	
Setting^e^	67% in private practice	
	21% in hospital	
	12% n.r.	
Employment^e^	53% self‐employed	
	40% employed	
	7% n.r.	
Focus of activity^e^	83% non‐academic	
	12% academic	
	5% n.r.	
PPE at least once/month^e^	39% in competitive athletes	
	75% in recreational athletes	
	2% n.r.	
Aware of the previous German PPE guideline^e^	43% aware of it and using it	
	28% aware of it	
	21% unaware of it	
	5% base the PPE on other sources/documents	
	3% n.r.	

Abbreviations: BMI = body mass index, n.r. = no response, PPE = pre‐participation evaluation.

^a^ Difference to 100% were missing answers.

^b^ Calculated from height and weight.

^c^ Hypertension, hypercholesterolaemia, diabetes, obesity (BMI ≥ 30).

^d^ Definition of physical activity levels according to McKay et al. [66]: rarely/never active = do not meet minimum activity guidelines/occasional and/or incidental physical activity; recreationally active = at least 150 min at a relaxed intensity or 75 min at a high intensity per week and muscle‐strengthening activities at least twice a week; trained = regular training approximately three times a week, with focus on a specific sport; highly trained = training and competing at the national level; elite = training and competing at the international level.

^e^ More than one answer possible.

#### 3.1.2 Sports Physicians' Survey

Sports physicians were recruited via the email newsletter of the DGSP, which reached 4148 sports physicians. With 205 physicians completing the survey, twice the recruitment target (*N* = 100) was achieved. One response was excluded due to the country of practice (Italy). The response rate was 4.9% (204 eligible respondents/4148 newsletter recipients).

The sample analysed included 186 physicians with and 18 without certification in sports medicine (Table 2).

### 3.2 Acceptability and Feasibility of the PPE

#### 3.2.1 Consumer Survey—Descriptive Results

**Figure 2:**
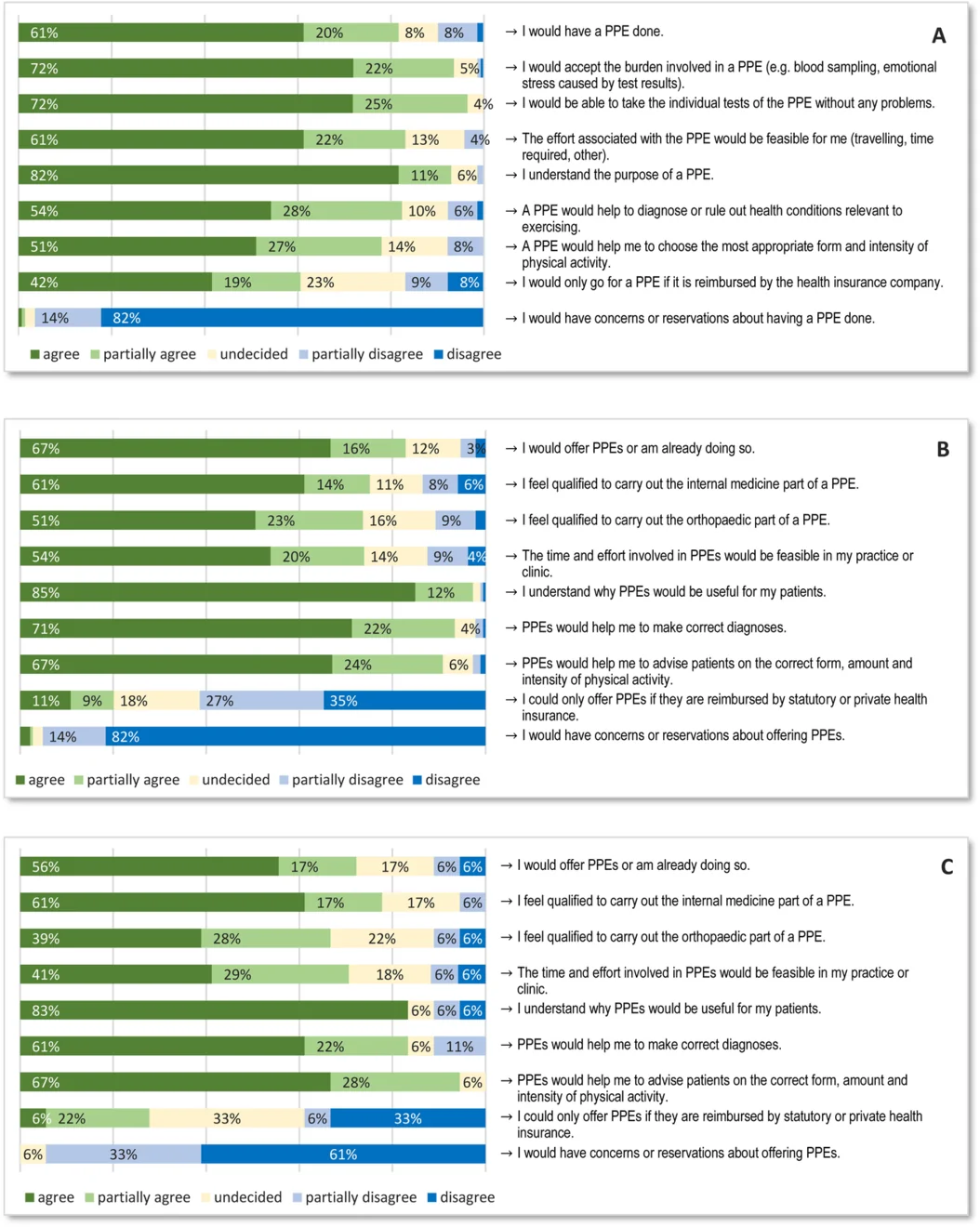
Rates of acceptability and feasibility of the PPE in percent, with number of responses received in brackets behind each question. (A) Acceptability of the PPE to consumers; (B) feasibility of the PPE according to sports physicians with certification and (C) without certification in sports medicine. Rates of 2% or less were omitted for clarity.

Of the consumers who responded to the acceptability questions (between 97% and 99% of survey participants for each question), 82% would consider participating in a PPE, 8% were neutral and 10% would not. Only 1% had ‘concerns or reservations’ (Figure 2A). A majority understood the purpose of a PPE. However, the results showed some variability in the perceived effectiveness in achieving the two objectives of the PPE, that is, to diagnose or rule out health conditions relevant to exercising (82% agreement, 8% disagreement) and to help choose the most appropriate form and intensity of physical activity (78% agreement, 8% disagreement). The most important barrier to acceptability was the potential non‐reimbursement of the PPE by health insurance companies.

#### 3.2.2 Sports Physicians' Survey

Of *N* = 204 physicians (*N* = 186 with/*N* = 18 without certification in sports medicine), between 98% and 99% responded to each feasibility question. Among them, 83% with/72% without certification would offer a PPE or already do so (Figure 2B,C). Certified sports physicians consistently understood the purpose of the PPE (97%), more so than physicians without certification (83%). Most respondents agreed with its goals, that is, to make correct diagnoses and to help advise patients on the best mode, amount and intensity of physical activity. Only 3% of physicians with and 0% without certification had ‘concerns or reservations’ about offering a PPE. Potential barriers to feasibility were non‐reimbursement of the examination by health insurance companies, the time and effort involved and the lack of qualification for the internal medicine or orthopaedic part of the PPE.

### 3.3 Findings From Comments on Draft Recommendations

All comments to the draft recommendations are provided in Supporting Information S1: Appendices 3 and 4.

#### 3.3.1 Consumer Survey

A total of 84/144 consumers (58%) used the free text fields to comment on draft recommendations. Some consumers highlighted that the acceptability of the PPE depended on a low access threshold and cost. A recreationally active participant suggested ‘*When joining a sports club/gym, include a recommendation for a (voluntary) sports examination in the admission contract, possibly with a reference to cooperating doctors. Covered by the health insurance company*’. Others proposed including the PPE in the regular GP check‐up examination.

Several participants stressed the increased need for sports medicine counselling for people with chronic diseases. A recreationally active participant with type 1 diabetes noted that ‘*for people with diabetes, a sports medical evaluation should urgently be included in the guidelines*’.

Some consumers emphasised that communication should be clear, understandable, and sensitive. A sedentary participant stated that ‘*the gynaecological history should be taken with sensitivity. Women, especially middle‐aged women, may have experienced difficult situations regarding certain questions in this area, e.g., birth, miscarriage*’. In addition, participants noted that their individual motivation to engage in physical activity should be strengthened during the PPE, for example, by documenting health improvements.

From the consumer perspective, the PPE should always lead to individual training recommendations. Goals and a way to achieve them should be agreed. A simple clearance statement was not considered acceptable by itself.

#### 3.3.2 Sports Physicians' Survey

A total of 162/186 sports physicians with certification (87%) and 17/18 without certification in sports medicine (94%) used the free text fields to comment on draft recommendations. Sports physicians argued that the feasibility of the PPE depends on its simplicity and coverage of the costs. Relevant quotes include that ‘*if the examination is too complex and therefore too expensive, too few people will use it*’.

Many also cited resources as a barrier to feasibility. Unequal geographical coverage was noted as leading to inequity in the provision of this preventive health examination. One respondent wrote: ‘*Unfortunately, daily practice in rural areas is increasingly characterised by the advanced age and multi‐morbidity of patients and a growing shortage of general practitioners. This leaves little room for the hobby of sports medicine*’.

Some respondents to the survey also highlighted the importance of taking a medical history and physical examination. These were also considered feasible in less well‐equipped medical practices. One respondent noted ‘*In my opinion, the mandatory instrumental requirements for sports medical check‐ups should be kept to a minimum. Medical history and clinical examination should be the main focus, as this makes a great deal possible (…). This can then be supplemented according to the medical history, findings, and age. After all, we are talking about screening almost the entire population*’.

In contrast, several respondents argued that diagnosis and individual training recommendations would be better achieved by including exercise stress testing in the PPE routine. Quotes by respondents include ‘*Stress ECG always, because objectification of the medical history*’.

Several respondents observed that another barrier to feasibility could be the different specialisations that precede a certification in sports medicine in Germany. One concluded that a PPE ‘*should be divided into two parts: internal medicine and orthopaedics. Both specialties do not understand enough about the other*’.

Several respondents also noted that the guideline recommendations would be easier to implement if they were more specific, and requested a history and examination form to be included with the guideline.

### 3.4 Use of Interest Holder Survey Results to Change Recommendations

**Figure 3:**
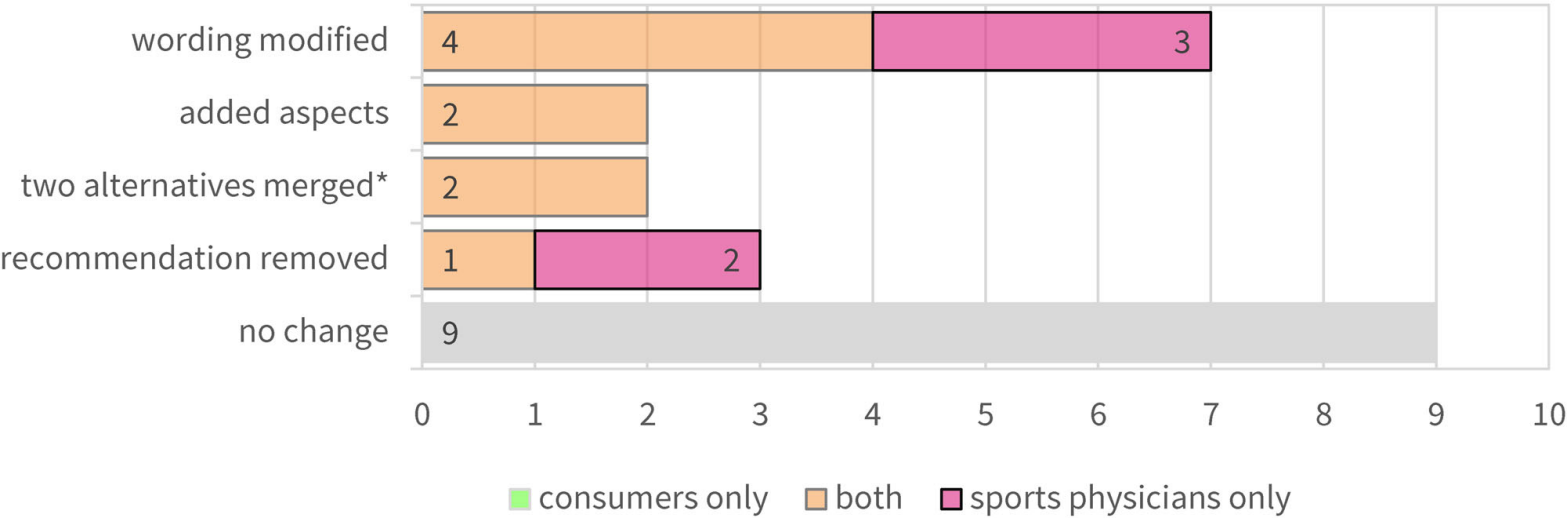
Modifications made to recommendations based on survey comments by interest holder groups (*N* = 23); *for three recommendations, we provided two alternatives for the wording.

Eleven (48%) of 23 draft guideline recommendations were modified as a result of survey feedback (Figure 3). Supporting Information S1: Appendix 5 lists all draft recommendations, a selection of relevant survey comments, and the resulting changes made to the recommendations. All recommendations were consensus‐based good practice statements. Changes were made to recommendations related to guideline questions on indications, timing, communication with patients, medical history, prognosis, clinical and laboratory assessment, instrumental diagnostics and referral to specialist care. There were no changes to recommendations related to the qualification of the healthcare provider, individual training recommendations, or clearance to participate in sports.

Most changes were made based on comments from both consumers and sports physicians (9/23, 39% of recommendations), none based on comments from consumers alone and four based on comments from sports physicians (17% of recommendations).

### 3.5 Use of Interest Holder Survey Results to Change Explanatory Text

In the explanatory text accompanying each guideline recommendation, the guideline authors highlighted important feedback from sports physicians and consumers whenever they considered it relevant to the interpretation of the recommendations, or when it provided useful implementation considerations.

## 4 Discussion

The majority of consumers considered the PPE to be acceptable and understood its goals. They identified potential non‐reimbursement as the main barrier. Other issues raised by consumers were first, the request for a low threshold to access the PPE, second, the increased need for sports medical counselling for people with chronic conditions, third, the desire for clear, understandable, and sensitive communication, and fourth, that the PPE should always lead to individualised training recommendations.

Most sports physicians considered the PPE to be feasible, but respondents highlighted three barriers to its successful implementation. These were its potential non‐reimbursement by health insurance companies, the additional burden on resources, and the lack of qualification for either the internal medicine or orthopaedic part of the PPE. In addition, sports physicians commented that to be feasible, the PPE should be simple and mainly consist of the history and physical examination. Beyond feasibility, several respondents expressed a preference for instrumental diagnostics, such as exercise testing.

The comments on individual recommendations showed variability in perceptions of which elements of a PPE were considered essential and which were not. The degree of variability mirrored the amount of discussion that took place within the guideline panel. The grades of recommendation were not changed as a result of the surveys.

The findings of our surveys largely agree with published studies on PPE. There is consensus in the literature that everyone should be motivated to exercise because of the extensive health benefits of physical activity [1, 2, 4, 6]. In our surveys, the PPE was seen as a tool to achieve this. In contrast to other studies [67, 68], the surveys did not indicate that interest holders perceived the PPE as an additional barrier to exercise. However, this in particular may be a consequence of selection biases in the recruitment to this survey. Because of the financial and opportunity costs associated with screening, the scope of testing is generally differentiated according to risk stratification [69, 70]. Many survey responses accepted that the scope of testing should depend on age, the presence of cardiovascular risk factors, previous injuries, and others. The responses by some participants, both consumers and sports physicians, deviated from the main tone of the published studies, asking for more extensive and regular testing as a means of objectifying diagnoses and documenting training progress, without regard to the associated cost of testing. The need for reassurance and specific guidance to identify the risks associated with exercising in people with chronic illness expressed in the surveys is also reflected in the literature [41, 71]. Mairoana and colleagues proposed to move away from medical ‘clearance’ for exercise programmes and towards physicians providing relevant clinical guidance to assess and manage any risks associated with exercise programmes [72]. This is in line with the expectations of the consumers who responded to our survey.

The survey results added value to the development of the German PPE guideline. The response rate to the acceptability and feasibility questions was high, with at most 3% of missing data for each interest holder group. They emphasised the main acceptability and feasibility issues to the guideline panel. The free‐text fields of each survey provided direct feedback on the recommendations from 84 consumers and 179 sports physicians. In both surveys, the high proportion of respondents to who chose to comment on the draft recommendations demonstrated the willingness of the interest holder groups to contribute to guideline content. The added content from a broader interest holders' perspective enriched the discussions during guideline development, and the guideline panel subsequently modified several proposed recommendations. In addition, the guideline authors included important interest holder feedback in the explanatory text.

### 4.1 Implications for Research

Although studies are difficult to conduct in the screening and prevention contexts [73, 74, 75], trustworthy recommendations require robust evidence from controlled studies. Further research is needed to clarify the effect of the PPE on patient‐relevant outcomes.

This study contributes to the currently scarce literature on the impact of consumer involvement in guideline development. Previous research has shown that it is difficult to assess the impact and outcomes of consumer involvement in guidelines, because it is often not reported [76, 77]. Reporting should improve to support meta‐research in this field.

### 4.2 Implications for Guideline Development

Our German experience with the conduct of consumer and guideline user surveys may inform the global guideline development community in several ways. First, we have shown how surveys can be co‐administered to multiple interest holder groups. Guideline developers may consider including further groups such as policy makers or payers of health services [78]. Second, we have found that surveys can help to incorporate consumer views into guideline development when recruiting consumer representatives into guideline panels is challenging, for example, when no consumer organisation exists, or when the consumer group is heterogeneous and a wider range of views is sought. This may apply particularly to prevention or screening guidelines. Third, interest holder surveys can serve to make consensus‐based recommendations more robust and trustworthy by including a variety of different perspectives particularly but not only when evidence is limited or absent. Fourth, comments collected from consumers and guideline users can be a valuable source of implementation considerations. For example, the survey results emphasised that offering a PPE to a wider population of healthy adults may require appreciable healthcare provider time that would no longer be available for diagnosis and treatment [79].

### 4.3 Implications for Health Policy

The findings highlight aspects relevant to the implementation of preventive health examinations in the field of sports medicine. They provided the perspective of interest holders on health policy issues such as accessibility, reimbursement and provider qualification. In this context, the call for the establishment of a standardised EU‐wide medical specialty in sports medicine gains relevance, as it would help ensure consistent screening standards and support cross‐border recognition of qualifications [80].

### 4.4 Limitations

The study has a number of limitations. First, as recruitment and administration of the surveys was online, the study may be subject to non‐coverage and self‐selection biases [81, 82, 83]. A comparison of the consumer sample to the general population in Germany indicated under‐representation of older and sedentary people, people with musculoskeletal or metabolic disorders including obesity, and men. Because older and sedentary people are at increased risk of adverse events related to physical exertion, their perspective would have been particularly relevant to the guideline. Exploratory statistical analysis did not, however, give any indication that the perspective in these groups differed significantly from the majority views. Second, social status and economic level of survey respondents was not captured in the survey, and could therefore not be analysed. Third, consumers' perceptions of the PPE may have been influenced by the information texts and videos about the PPE that we included in the recruitment materials and on the first page of the survey. Fourth, the individual comments were varied and often contradictory, and their aggregation may have led to oversimplifications. Fifth, the difficulties in recruiting a larger number of consumers from the general population in the time available probably resulted in a public involvement group that was not statistically representative. Sixth, because the comments from consumers and sports physicians were presented to the guideline working groups at the same time, we are unable to say whether consumer comments alone would have influenced guideline development to the same extent. Seventh, the national setting in Germany may limit the generalisability of the results to countries with different healthcare systems and different attitudes towards preventive health examinations. Finally, the applicability of the results to other contexts may be limited by the design of the underlying guideline, which was developed through a formal consensus process but without a systematic review of the literature [84].

## 5 Conclusions

Our survey study found the PPE to be acceptable to consumers as long as costs are covered by their health insurance. Most sports physicians perceived the PPE to be feasible, but some had concerns about reimbursement, resources, and qualification issues. The comments received raised awareness of the main acceptability and feasibility issues during the development of the German PPE guideline and led to modifications of the draft recommendations.

## Author Contributions

Conceptualisation and methodology: Käthe Goossen, Alina Weise, Jessica Breuing, Anja Hirschmüller, Christine Joisten and Nadja Koensgen. Data acquisition and interpretation: Sandra Jaax, Käthe Goossen, Alina Weise, Anja Hirschmüller and Charlotte Kreutz. Writing – original draft: Käthe Goossen. Writing – review and editing: Käthe Goossen, Alina Weise, Anja Hirschmüller, Sandra Jaax, Charlotte Kreutz, Christine Joisten, Nadja Koensgen and Jessica Breuing.

## Ethics Statement

The surveys received approval from the Ethics Committee of the University of Freiburg (Reference Number 23‐1369‐S2).

## Consent

Survey participants were informed that by submitting their answers to the survey, they would provide consent to participate and consent for scientific publication of anonymous results. Participants were able to withdraw from the survey at any time prior to submitting their answers.

## Conflicts of Interest

C.J. is president of the DGSP. A.H. is a member of the scientific advisory committee of the DGSP. K.G. is a member of GIN Public. K.G. and A.W. were involved in guideline development as methodologists. S.J., C.K., N.K. and J.B. have no conflicts of interest.

## Registration

A protocol was registered with the Open Science Foundation prior to initiation of the survey study. It is accessible via https://doi.org/10.17605/OSF.IO/DZA6G.

## Supporting information

SupportingyFile1.

Supporting‐File‐2_Consumer‐survey_English_revised.

Supporting‐File‐3_Sports‐physicians‐survey_English_revised.

## Data Availability

The data sets used during the current study are available in the Online Supporting Information or from the corresponding author on reasonable request.
