# Supplementary material for: Sports Pre‐Participation Evaluation in Healthy Adults—A Survey‐Based Assessment of Guideline Acceptability and Feasibility Among Sports Physicians and Participants
Source: Clin Public Health Guidel. 2025 Aug 20;2(4):e70032. doi: 10.1002/gin2.70032 (PMC13563407; doi:10.1002/gin2.70032)
Supplement: Supplementary file 1 [file GIN2-2-e70032-s001.pdf]

# Supplementary File 1


Appendix 1. Consumer survey recruitment .......... 2

Appendix 2. Details of exploratory statistical analysis of the consumer survey .......... 2

Appendix 3. Comments on draft recommendations from the consumer perspective .......... 5

Appendix 4. Comments on draft recommendations from the physicians' perspective .......... 38

Appendix 5. Effect of consumer and sports physicians’ surveys on recommendations .......... 78

## Appendix 1. Consumer survey recruitment

**Table** List of organisations contacted for the recruitment of consumers

| | |
|---|---|
| **Sports clubs and federations** | German Olympic Sports Confederation (Deutscher Olympischer Sportbund) |
| | State sports associations of all 16 German federal states ('Landessportbunde') |
| | Sports associations for people with disabilities of all 16 German federal states ('Behindertensportverbände') |
| **Health insurance providers** | Statutory health insurance providers (Audi BKK, Bergische Krankenkasse, BMW BKK, BKK Herkules, BKK Wirtschaft und Finanzen, energie-BKK, Mobil Krankenkasse, Siemens BKK, Viactiv Krankenkasse, WMF BKK) |
| **Academic institutions** | German Sport University Cologne |
| | Witten/Herdecke University |
| **Other** | Customer advice centres of all 16 German federal states |
| | Social media channel of the DGSP |
| | General social media (dedicated Instagram channel and Facebook page, personal WhatsApp networks) |

## Appendix 2. Details of exploratory statistical analysis of the consumer survey

### Aims

Exploratory statistical analysis (binary logistic regression) was carried out with the aim to estimate whether the perspective of under-represented groups in the sample (older adults, sedentary people, obese people) differed from that of the majority of the sample.

In addition, some of the sports physicians raised concerns about the feasibility of the PPE at a national scale: if a substantial proportion of the adult population were to take up the offer of a PPE, the available resources would be insufficient to deliver it alongside the daily routine in sports-medical practices. We used multivariate binary logistic regression to analyse the responses of sedentary or recreationally active adults on whether they "would have a PPE done". Our hypothesis was that some physical activity goals (see Table 1), particularly an increase in intensity or frequency, would correlate with a positive response to this question.

### Methods

All analyses were carried out using IBM SPSS Statistics (Version 28).

From the full dataset, we included only adults who were sedentary or recreationally active. We excluded data for children and for consumers who were athletes at the national or international level (Figure 1).

Acceptability of the PPE was assessed using agreement to the statement "I would have a PPE done" on a 5-point Likert scale using the following values:

1: does not apply ("trifft nicht zu")
2: mostly does not apply ("trifft eher nicht zu")
3: partially applies ("teils-teils")
4: mostly applies ("trifft eher zu")
5: applies ("trifft zu")

Values of 4 or 5 on the Likert scale were categorised as agreement, values of 1 to 3 as non-agreement.

Continuous variables were categorised for analysis (Table 1).

Multivariate analysis limited to 5 independent dichotomous variables due to the small sample size (*N*=130) and low number of events (N=25 consumers with non-agreement, corresponding to values of 1-3 on the Likert scale) [1, 2].

We analysed acceptability of the PPE using agreement to the statement "I would have a PPE done" as the dependent variable in binary logistic regression analysis with the following independent categorical variables: age (<60 vs. ≥60 years), BMI (<30 vs. ≥30), chronic condition (no vs. yes), activity level (rarely/never active vs. recreationally active vs. trained), and goal (maintain level of activity vs. take up or return to sport vs. be active more often vs. be active at higher intensity vs. participate in competitions) (Tables 2 and 3).

**Results**

**Figure 1.** Flow chart for statistical analysis of the consumer survey

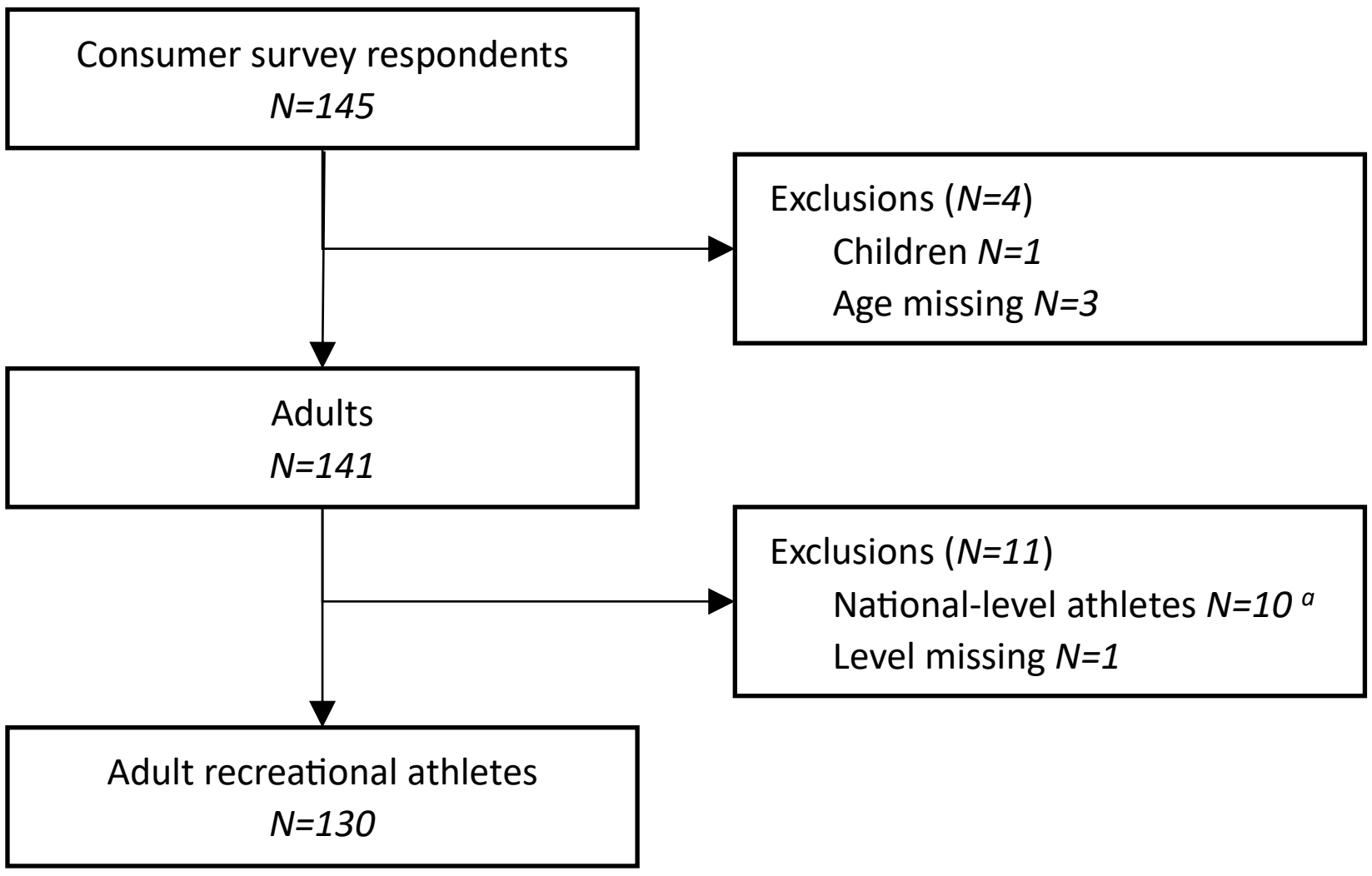


[a] National-level athletes (highly trained), defined as training and competing at the national level; there were no elite athletes, defined as training and competing at the international level.

After exclusion of responses for adults who compete at the national or international level (*N*=10) and people with missing data for age (*N*=3) or level of physical activity (*N*=1), the sample contained *N*=130 adults who were sedentary or recreationally active (Table 1).

**Table 1**. Characteristics of the sample used for statistical analysis (N=130).

| | Category | n (%) | Missing data, n (%) |
|---|---|---|---|
| Agreement (dependent variable) | agreement (Likert 4-5)<br>non-agreement (Likert 1-3) | 105 (80.8)<br>25 (19.2) | – |
| Age | <60 [a]<br>≥60 | 113 (86.9)<br>17 (13.1) | – |
| BMI | <30 [a]<br>≥30 (adipose) | 117 (90.0)<br>13 (10.0) | – |
| Chronic health condition | no [a]<br>yes | 99 (76.2)<br>31 (23.8) | – |
| Physical activity level [b] | recreationally active or trained [a]<br>sedentary | 101 (77.7)<br>29 (22.3) | – |
| Goal of physical activity | maintain level of activity [a]<br>increase level of activity | 32 (24.6)<br>97 (76.4) | 1 (0.8) |
| Goal of physical activity (sensitivity analysis) | maintain level of activity [a]<br>take up or return to sport<br>be active more often<br>be active at higher intensity<br>participate in competitions | 32 (24.6)<br>21 (16.2)<br>30 (23.1)<br>25 (19.2)<br>21 (16.2) | 1 (0.8) |

[a] *Reference category for logistic regression analysis.* [b] Definition of physical activity levels according to [3]: sedentary (rarely/never active), defined as do not meet minimum activity guidelines/occasional and/or incidental physical activity; recreationally active, defined as at least 150 minutes at a relaxed intensity or 75 minutes at a high intensity per week AND

muscle-strengthening activities at least twice a week; trained, defined as regular training approximately 3 times a week, with focus on a specific sport.

Multivariable binary logistic regression analysis showed no significant association of acceptability with older age, body mass index (BMI) ≥30, a chronic health condition, or a sedentary lifestyle (Tables 2 and 3). The analysis also indicated that people who had the goal of increasing their level of activity were about 3 times more likely to consider having a PPE done than people who planned to maintain their level of physical activity (Table 2). A sensitivity analysis indicated that this was driven mainly by people currently inactive, who intended to take up or return to sports (Table 3).

**Table 2**. Results of binary logistic regression analysis, main analysis using 2 categories for goal of physical activity (N=130).

| | Category | adjusted OR [95% CI] |
|---|---|---|
| Age | <60 | (reference category) |
| | ≥60 | 0.48 [0.13-1.81] |
| BMI | <30 | (reference category) |
| | ≥30 (adipose) | 2.71 [0.29-25.17] |
| Chronic health condition | no | (reference category) |
| | yes | 1.60 [0.43-5.91] |
| Physical activity level | recreationally active or trained | (reference category) |
| | sedentary | 0.86 [0.24-3.05] |
| Goal of physical activity | maintain level of activity | (reference category) |
| | increase level of activity | 2.95 [1.08-8.04] |

CI=confidence interval; OR=odds ratio.

**Table 3**. Results of binary logistic regression analysis, sensitivity analysis using 5 categories for goal of physical activity (N=130).

| | Category | adjusted OR [95% CI] |
|---|---|---|
| Age | <60 | (reference category) |
| | ≥60 | 0.41 [0.10-1.63] |
| BMI | <30 | (reference category) |
| | ≥30 (adipose) | 2.01 [0.20-20.53] |
| Chronic health condition | no | (reference category) |
| | yes | 1.76 [0.45-6.81] |
| Physical activity level | recreationally active or trained | (reference category) |
| | sedentary | 0.57 [0.12-2.75] |
| Goal of physical activity (sensitivity analysis) | maintain level of activity | (reference category) |
| | take up or return to sport | 11.74 [0.95-145.79] |
| | be active more often | 1.94 [0.53-7.04] |
| | be active at higher intensity | 3.31 [0.76-14.35] |
| | participate in competitions | 2.77 [0.65-11.90] |

CI=confidence interval; OR=odds ratio.

**References**


1. Freedman, D.A. and D.A. Freedman, *A Note on Screening Regression Equations.* The American Statistician, 1983. **37**(2): p. 152-155.
2. Vittinghoff, E. and C.E. McCulloch, *Relaxing the rule of ten events per variable in logistic and Cox regression.* Am J Epidemiol, 2007. **165**(6): p. 710-8.
3. McKay, A.K.A., et al., *Defining Training and Performance Caliber: A Participant Classification Framework.* Int J Sports Physiol Perform, 2022. **17**(2): p. 317-331.

# Appendix 3. Comments on draft recommendations from the consumer perspective

All text was translated with www.DeepL.com/Translator (free version) with manual modifications.

**1 Alternative A: Offer a sports medical check-up to adults who want to take up or return to sport.**

**Important from the citizen's point of view**

- The offer should be very low-threshold for popular sports so that it is accepted. I fear that many people will forego it if this examination is associated with costs. Suggestion: When joining a sports club/gym, include a recommendation for a (voluntary) sports examination in the admission contract, possibly with a reference to cooperating doctors. Covered by the health insurance company.
- "With a list of available sports doctors in the neighbourhood

**Training recommendations as a result of the examination**

- Definitely useful, especially if training recommendations are generated from the results. Simply stating "Can do sport" or "Should not do sport" is not helpful.

**For both (re)starting and for regular sport**

- The separation of the two variants in terms of content is not clear to me - if preventive examinations are justifiable for both (new) beginners and active athletes, a combined recommendation could also be made, focussing on active and future athletes.
- Recommendation 1, alternative A and B are two different questions for me. Is there an offer for adults who are starting out in sport or who have already been doing sport for a certain period of time? The offer for both groups would be desirable in my view
- Adults who want to start or return to sport or who do sport should be offered a sports medical check-up.

**Preference for alternative B ('Offer a sports medical check-up to adults who do sport.')**

- No. Only after a certain length of time in sport. Otherwise no space/appointments for those who need it.
- It should also be offered to adults who are not starting sport again but want to increase the frequency/intensity.

**To be clarified in the text**

- Perhaps include who can carry out such examinations and how you can be referred to the relevant doctor (direct appointment/referral by a doctor).
- I think the term "starting sport" is too soft, if someone signs up to the gym to do light exercise once a week, that would be too much for me. I would argue in favour of specifying the intensity of the sport.

**Suggested editorial changes**

- Replace "again" with "after a longer period of time"?

**Costs**

- "The costs of the preventive medical check-up are to be covered by the health insurance funds and the subsidy funds.

**Agreement, encouragement**

- I think it's very important that if a new athlete wants to, they can start and do so safely. No overloading through "internet training plans" etc. Get to know the stress limits of the moment.
- I think it's very good to be able to rule out possible cardiovascular diseases beforehand and help patients to recommend the right intensity of training

- I find it useful to be able to rule out any existing cardiovascular diseases. It also makes sense to find the right sport for you.
- Yes, this makes sense, as you may be at an increased risk of injury after a break from sport.

**Depending on the type of sport or training intensity**

- I think the intensity and frequency of the sport should be taken into account. I think that a sports medical examination would not be necessary for people who only do light sport 1-2 times a week. Or I would call it general health care - something that would be good for everyone, regardless of whether they do sport or not.
- Depends on the sport

**Strength of the recommendation**

- I think the recommendation should be changed to "can".

**Simple approval of alternative A (‘Offer a sports medical check-up to adults who want to take up or return to sport.’)**

- definitely
- This wording is clearer than alternative B"
- I find it more accurate.
- Yes
- Yes
- Yes
- Yes
- Useful
- Agree
- Agree
- great
- I support
- Alternative A makes more sense to me as it is formulated more concretely.

**Unclear**

- Offer definitely, but not a must, I expect previous knowledge from athletes who have already been involved in sport

**Target group: children**

- A medical check-up is not only necessary for adults but also for children! Accordingly, it should be called: People who want to start or return to sport ..."
- Why only adults? Children and adolescents should not be excluded.

**1 Alternative B: Offer a sports medical check-up to adults who do sport.**

---

**Important from a citizen's point of view**

- Here, too, I see the clubs / studios as having a duty. The offer must be very low-threshold, otherwise the examination will not take place.
- I think it would be great if this were a point in the check-up[1]...

**Both for (re)starting and for regular sport**

- ... who do sport or would like to start in the future...

---

[1] the „check-up“ is a regular preventive medical examination by a general practitioner, which is offered to people with statutory health insurance every three years

- Recommendation 1, alternatives A and B are 2 different questions for me. Is there an offer for adults who are starting to do sport or who have already been doing sport for a certain period of time? The offer for both groups would be desirable from my point of view
- I would favour this alternative and add "or would like to start".
- I think regular examinations are important. For everyone.

**Preference for alternative A ('Offer a sports medical check-up to adults who want to take up or return to sport.')**

- That would be too much for me in the context of statutory health insurance, it should be specified by chronic illnesses or similar.
- I think this alternative is too general compared to A; I feel much less directly addressed here.
- alternative A, on the other hand, is more open and has a preventative effect, for example for people who have had a longer break from sport and may overestimate their body without an examination

**Preference for alternative B ('Offer a sports medical check-up to adults who do sport.')**

- ... anyway, as the load limits shift. Upwards through training, downwards again with age. Flu etc. as lung stress.
- Once a year
- As a voluntary offer, this is certainly a good way to recognise possible overloads/incorrect loads in good time and, if necessary, take countermeasures through advice
- Basic services for generally active athletes. More services for athletes at a higher level. And not only raise the level according to squad status.
- More suitable than alternative A. People who are already active in sport can also train in a way that is detrimental to their health and benefit from a sports medical examination
- Better, as all adults are included! Active athletes in particular benefit from regular sports medical examinations. In this way, sporting activities can be specifically adapted to individual comorbidities and thus preventively counteract joint diseases, for example, through exercises/training units that are gentle on the joints. Currently, athletes often only go to the doctor when they have complaints such as tibial crest syndrome and are then told how they can take preventative action. By then, however, it is often too late and, in the worst case, the condition has already manifested itself or become chronic. This could be prevented by sports medical examinations, which also include preventative training. This is particularly useful in amateur sport!
- I think it's good, regular offers for competitions would also be good
- Yes, especially if the state of health changes
- Yes, because professional support can improve adherence and continue to support athletes in the future.
- Yes, because a lot can go wrong, especially when training in fitness centre chains.
- Makes sense above all if it is carried out regularly and training recommendations are adapted. The frequency should be discussed with the doctor depending on any existing risk factors or pre-existing conditions.
- Very good, everyone can then decide for themselves whether they want to do it
- Sensible recommendation, because the body and performance goals often change, even in long-term athletes.

**Preference for an examination only if necessary**

- If necessary
- I don't think it's necessary for all adults who do sport to have this examination, as those who have been doing sport for a long time may already be aware of their body and the risks of sport.
- Not necessarily everyone - if someone has been doing their favourite sport for a long time and is doing well, then everything is fine

**To be clarified in the text**

- Who are adults? From the age of 18?

**Strength of the recommendation**

- "I think the recommendation should be changed to ""can"".

**Costs**

- "The costs of the preventive medical check-up are to be covered by the health insurance funds and the subsidy funds.
- If it can be billed via the health insurance fund, this is a good thing, similar to preventive medical check-ups with specialists

**Simple agreement with alternative B ('Offer a sports medical check-up to adults who do sport.')**

- also great
- Yes
- Yes
- Useful
- Agree
- I support

**Disagreement of alternative B ('Offer a sports medical check-up to adults who do sport.') with reasons**

- No prevention in this case
- Sport reduces the risk of illness. Preventive examinations should be justified in particular by risks."

**Simple disagreement of alternative B ('Offer a sports medical check-up to adults who do sport.')**

- No

**Unclear**

- I find it useful to be able to rule out any existing cardiovascular diseases. Also useful for finding the right sport for you.

**Target group children**

- A preventive check-up is not only necessary for adults but also for children! Accordingly, it should be called: People who are new to sport or want to take up sport again should be offered a sports medical check-up. The check-up should be repeated at regular intervals (annually for children and adolescents up to and including the age of 17, and every two years for people aged 18 and over)."

From the citizens' perspective.

- A low-threshold offer is desired, especially for recreational sports, possibly in combination with the health check-up.

**2 Alternative A: Use the relevant guidelines for people with chronic diseases.**

**2 Alternative B: Use the relevant guidelines for people with diseases.**

**Clearer wording required**

- That depends: If only chronic diseases are really explicitly meant/relevant, then it should be worded as such.
- "Overall unclear objective of the recommendation (use of the guidelines for sports medical check-ups or use of the specific guidelines for the treatment of existing illnesses) - further comment assumes that it is a matter of applying the guidelines for sports medical check-ups.
- With pre-existing conditions

- confusing - what conditions are meant here as opposed to "chronic conditions"? It would not be clear to me to what extent acute illnesses are relevant with regard to the indication of sports medical examinations... Then I'll just go when I'm healthy again...?

**Preference for a general sports examination regardless of existing illnesses**

- Yes, why only chronic ones? Better general
- Shouldn't such guidelines apply to everyone who has such check-ups, regardless of illness, whether chronic or not?

**Preference for a sports examination for people with any illness**

- Prefer all diseases
- I think this alternative is better because it applies to all illnesses
- I think it's better because it includes acute AND chronic illnesses.
- I think that the guidelines should be applied to all illnesses.
- Yes, because the guidelines should be taken into account by specialised doctors for all illnesses.
- Sensible, as certain illnesses do not permit all sports.
- Sensible, every adult should have the opportunity for a sports medical examination if sport is practised

**Preference for a general sports examination with specific elements for the respective (also non-chronic) illness**

- Why only people with chronic illnesses? A basic examination for everyone and then more specialised tests for certain illnesses (not just chronic ones) would make sense.
- I think it would be better, as I don't understand why only chronic illnesses should be taken into account.
- The same applies here as with 2A, whereby the possible risks of resuming sport too early or too intensively should also be explained. Depending on the type of sport and sporting goals, specialist sports support should also be referred to if necessary.

**Preference for a sports examination for people with chronic illnesses**

- ... sorry, I don't know, but in the case of chronic illnesses this is actually already obligatory.
- Chronic illnesses are a better definition than illnesses.
- It's probably clear that people with acute COVID-19 illness don't train. But I definitely see the need to focus on chronic diseases.
- Recommendations for chronic illnesses are more relevant, whereas preventive sports medicine is not a priority for acute illnesses.
- I think this (alternative B) tends to be formulated too generally - after all, "illnesses" can be all sorts of things, e.g. a temporary cold.
- alternative A is more precise

**Preference for using the sports examination guideline with specific elements for chronic diseases**

- Rather use guidelines specifically focussed on sport, the general guidelines contain too little information about sport
- Preference for alternative A - diseases that are irrelevant in terms of content, especially acute ones, do not require extensive consideration."

**Preference for the use of sports medicine elements in guidelines on chronic diseases (possibly supplemented by the guideline on sports examinations)**

- Definitely, and professional training counselling / training support should always be used.
- I think that's appropriate. Does every guideline have a passage on exercise?
- For people with diabetes, a sports medical evaluation should urgently be included in the guidelines.
- Unfortunately, the guidelines for people with type 1 diabetes contain far too little about exercise, no fitness trainer knows anything about it and doctors avoid the subject. They don't know anything about it. However, there is sufficient evidence on the topic, it could very well be reworked into guidelines, and

education and training programmes could be developed and offered. We had already applied for an innovation fund for this, which was unfortunately rejected.
- This makes sense, as certain chronic illnesses do not permit all sports.
- If the relevant guideline does not cover topics relating to sports medicine screening, then the other guideline should be used.

**Preference for the use of guidelines for chronic diseases**

- Is that the assumption?
- If they are S3 guidelines definitely! For others, there should at least be an orientation towards the guidelines
- Yes, the guideline for the respective disease should be used.
- "Should guidelines not always be used?

**Preference for the use of guidelines for any disease**

- "Should guidelines not always be used?
- Yes, the guideline for the respective disease should be used.

**To be clarified in the text**

- Define diseases!
- Simple approval of alternative vs. Good idea
- Yes
- Yes
- Yes
- Yes
- Useful
- Agree
- Agree
- Absolutely

**Simple agreement with alternative B ('Use the relevant guidelines for people with diseases.')**

- I prefer this more general formulation.
- Yes
- Yes
- Yes
- Yes
- Yes that sounds sensible
- Yes

**Simple disagreement of alternative B ('Use the relevant guidelines for people with diseases.')**

- No

**Unclear**

- Depends on the illness
- I think an individualised plan makes much more sense... what helps with A doesn't necessarily help with B. It depends on the different physiques alone

| From the citizens' perspective.<br>• There is an increased need for sports medicine counselling for people with chronic illnesses. |
|---|

**3 A sports medical check-up should be conducted when starting a more intensive sports and exercise programme.**

**Important from a citizen's point of view**

- I would find it more useful in the check-up and better information on the topic.

**To be clarified in the text**

- In general? From a certain age?
- Support in terms of content - I only see overlaps with recommendation 1, which recommends an offer. This implicitly recommends the actual implementation of the programme.
- What is an intensive sports programme?

**Suggested editorial changes**

- "Should be conducted" sounds very strict and prescriptive. I would rather suggest "is recommended" or similar.
- "Before" starting instead of "when" starting?
- Also.

**Agreement, encouragement**

- Because intensive sports units begin with a slow build-up. A sports check-up would be useful beforehand
- That sounds more precise to me than above
- Yes, it would be desirable for this to always be part of programmes
- If the volume or load is to be increased significantly within a short period of time, it is advisable to have an examination. The examination should then also result in recommendations for training control/adaptation and any necessary compensatory training to avoid consequential damage.

**Restricted agreement depending on age, risk factors and type of sport**

- From a certain age (e.g. 40 years) in order to rule out typical illnesses such as hypertension, diabetes, obesity or similar beforehand or to specifically adapt the exercise and sports programme accordingly
- also for normal sporting activities
- If necessary
- That depends on how old I am and what sport I want to do.
- Yes, but it depends on your age and state of health.
- Depending on how long it's been since your last check-up
- Agree not absolutely necessary

**Strength of the recommendation**

- "I think the recommendation should be changed to ""can"". Keep barriers to sport low!"

**Simple agreement**

- Definitely
- Definitely!!!
- I think that's good.
- Yes
- Yes,
- Yes. Definitely
- Yes, it's just better
- Yes.
- fits
- Agree
- Good
- Agree

**Simple disagreement**

- No

**Target group Children**

- The preventive medical check-up must be repeated annually for children and adolescents up to and including the age of 17, and every two years for persons aged 18 and over.

| From the citizens' perspective. |
|---|
| • Information on the topic of sports medical check-ups is desired.<br>• The intensity and frequency of sport should be taken into account when organising the examination. |

**4 Alternative A: A sports medical check-up should be repeated regularly.**

**Proposed editorial changes**

- if necessary.
- Suggested improvement: ...can be repeated regularly for long-term planned or sustained sporting activity

**Agreement, preference for this alternative (individual time interval, depending on risk profile/sport type)**

- Definitely, especially to make effects measurable for beginners
- "For competitive athletes, this will hopefully be done anyway. Otherwise, an examination at regular intervals should be decided on an individual basis"
- Regular repetition always makes sense. The frequency of repetition should be discussed with your doctor depending on risk factors, previous illnesses / injuries and the level of sporting activity (pure recreational sport, ambitious recreational athlete or competitive athlete). as well as the extent of the repeat examination.
- I prefer this formulation because there can be constant changes in the organism/movement apparatus that require an adjustment of sporting movement/sporting behaviour.
- Yes, with restrictions. Depending on the type of sport practised, among other things.
- Yes and no, only if someone actually does sport. A little "kicking" is not the sport.
- Depending on how long it's been since the last examination
- Depends on the patient and their physical condition. If the first examination was unremarkable, I don't think it necessarily has to be repeated or perhaps only every 5 years.
- Correct in my eyes.
- It makes sense, as the ravages of time gnaw at everything, including yourself. Perhaps a sports medical check-up can help you to find out at an early stage whether you can still do the sport or type of sport you have chosen.
- What is regular? In addition, the need for repeat examinations is certainly different for someone with chronic pre-existing conditions than for someone who is 22 years young and healthy. Unless, again, it is a high performance athlete.

**Preference for the alternative (specify time interval)**

- "Regularly" is too vague: every year? Every 3 years? Every 10 years?
- The preventive medical check-up must be repeated annually for children and adolescents up to and including the age of 17, and every two years for persons aged 18 and over.
- At what frequency
- Yes, once a year
- This is the only way to ensure long-term prevention. This is the only way to determine the correct performance level and thus control training. Once a year. For competitive athletes (also at NRW2 level) twice a year.

---

[2] regional level (NRW=North-Rhine Westphalia)

- Regular seems vague if it is not more specific. Is it then still preventive or concomitant? It says at the top for admission.
- Define time interval!
- Too unspecific regarding regular

**To be clarified in the text**

- In general? From what age?

**Simple agreement**

- Definitely.
- YES!!!
- In favour

**Disagreement, proposed modifications**

- I think it is enough to do this once or when there is a change
- Indispensable in competitive sport, not relevant for the average athlete
- Not absolutely necessary, rather on one's own initiative
- Only for a risk profile.

**Simple disagreement**

- no
- No

## 4 Alternative B: A sports medical check-up should be repeated regularly at intervals of 1-3 years.

**Even more specific time interval**

- 1 year is too short, 3 years is conceivable.
- This is also too general for me, without specifying the disease
- "The screening examination should be repeated at regular intervals (annually for children and adolescents up to and including the age of 17, every two years for people aged 18 and over).
- In my opinion, repeating a corresponding examination every three years is not sufficient, especially for children and adolescents."
- I find the period 1-3 years too vague.
- More specific, but still broad
- That's far too little considering the enormous strain of sport and all kinds of infections.
- Right, preferably every year

**Agree, preference for this alternative ('A sports medical check-up should be repeated regularly at intervals of 1-3 years.')**

- For insulin-dependent diabetes definitely
- Better than vs. Better than alternative A, as concrete time specifications define a framework and may lead to a reminder ("Oh yes, every 1-3 years")
- Better defined!
- This is specific in the intervals, I think that's better
- This alternative is better because it is concrete.
- I would choose this alternative as it is more precise.
- Interval specification is good
- Is more precise than alternative A. I like this alternative better because it is clearer what is meant by regular.
- Yes, much more precise and sensible.
- I think it's better, even every 10 years is "regular". The car also has to be MOT'd every 2 years. I think that's a good interval.

**Preference for the alternative (individual time interval, depending on risk profile/sport)**

- Comment for alternative A (For competitive athletes, this will hopefully be done anyway. Otherwise, an examination at regular intervals should be decided on an individual basis)
- Definitely, the time intervals should be individually agreed with the doctor. See answer 4A. (Regular repetition always makes sense. The frequency of repetition should be clarified with the respective doctor depending on risk factors, previous illnesses / injuries and the extent of the sporting activity (pure recreational sport, ambitious recreational athlete or competitive athlete). as well as the extent of the repeat examination).
- For people with previous illnesses or competitive athletes, yes. Otherwise I would recommend 5 years
- Is the time interval realistic? Presumably more needs-orientated, or every 5 years except for strenuous sports (boxing, marathons, etc.)?
- Would have said 5 years if the findings were normal...or depending on the sport...

**Simple agreement**

- I think that's good
- Yes
- Yes
- Yes
- Yes
- YES!!!
- sounds good, no idea
- I agree

**Agreement, encouragement**

- Good idea! And the health insurance company could give you points in your bonus booklet for this.
- Very good, things can always change, whether for the better or worse

**Disagreement, proposed modifications**

- Only with a risk profile.

**Simple disagreement**

- I think that's exaggerated.
- No
- No

| From the citizens' perspective.<br>• Information on individual examination intervals is requested. |
|---|

**5 Also use the results of the sports medical check-up to derive individualised exercise and training recommendations.**

**Important from a citizen's point of view**

- Definitely. It would be great to be able to get FTP, VOmax, maxHF
- Definitely and important so that participants know what the test is for.
- Isn't that what it's for?
- The findings should be incorporated into the training plan
- This is exactly why they are valuable for recreational athletes - how often they train, how intensively, etc
- Good, so the examination also makes sense for the patient beyond the exclusion of potential pre-existing conditions
- Yes, to see what is feasible and what is not or to see where there are deficits that need to be compensated for.

- This is the only way a preventive examination makes sense. Simply stating whether a person can / may / should do sport or not is usually of little use to the patient in question as they usually have no other qualified contact person (trainer etc.) to help them organise their training.
- Absolutely! I don't think a sports medical check-up without a recommendation makes sense!

**Individualised recommendation preferred**

- Again, an individual decision if it is important for a sports programme
- Depends on the individual case

**Proposed editorial changes**

- Term "derivation" awkward, in my opinion better "subsequent creation"
- It is unclear to me what is meant by "derivation".
- Couldn't you leave out "Bewgungs"?

**To be clarified in the text**

- Full agreement! Perhaps define who makes these recommendations (sports doctor, physiotherapist, GP, etc.).

**Simple agreement**

- +
- Definitely
- Definitely
- Definitely
- I find that very plausible
- I think it's good
- Well understandable.
- Yes
- Yes
- Yes
- Yes
- Yes!
- Yes.
- Sounds plausible.
- Agree
- agree
- Absolutely
- Absolutely.
- Would make sense
- Agree

**Agreement, doubts about feasibility**

- Ideally, yes. However, this would require doctors to have comprehensive knowledge of the sport or trainers to be able to correctly assess the examination results. I doubt that this can be realised in reality for every examination and think that this can only take place in high performance sport.

**Unclear**

- ???

From the citizens' perspective.

- The sports medical check-up is considered useful if training recommendations are generated from it. The mere statement "Can do sport" or "Should not do sport" does not seem helpful.

**6 Evidence-based communication strategies should be used to convey exercise recommendations.**

**Important from a citizen's point of view**

- Provide a report at the end so that you can read up on what you have forgotten or not heard
- Yes, but at eye level
- Makes sense, but the goal and the way to get there should still be communicated clearly and comprehensibly.

**Individualised communication preferred**

- Suitable strategies should be used for the respective target group
- In my opinion, it should be emphasised here that the communication should be adapted to the individual needs/communication style of the respondents.

**To be clarified in the text**

- "evidence-based" might be difficult for some people to understand.
- Examples?
- Info field on "evidence-based" important, give example if necessary
- Why?
- What is an evidence-based communication strategy?

**Agreement, encouragement**

- These are always helpful for every patient contact
- Very useful, especially for new doctor-patient relationships.
- Always useful.

**Simple agreement**

- with pleasure.
- Good
- Yes
- Yes
- Yes.
- Yes.
- Yes
- Yes
- Very useful
- Agree
- Absolutely
- Agree
- Agree

**Partial agreement**

- I don't think it's absolutely necessary, but okay.
- Would be good, I doubt the application.

**Disagreement, with reasons**

- I find the wording difficult; there are well-known communication concepts that (can) show no evidence and yet appear to make sense. I find the idea of quantifying interaction and simply making it measurable strange.
- Perhaps a little exaggerated if you start with a general sports medicine check-up

**Unclear**

- Better co-operation with GP and orthopaedists.

- I can't make much sense of that. Sounds too technical to me.

From the citizens' perspective.

- Communication should be clear and understandable and take place on an equal footing.
- Goals should be agreed and a way to achieve them should be communicated.

**7 Alternative A: A sports medical check-up should be carried out by a specialised doctor, preferably with certification in sports medicine.**

**Disagreement, qualification should be defined more broadly**

- General practitioners should also be able to offer the examination.
- "I think that's over the top. First of all, a general preliminary sports examination would be important for as many interested athletes as possible. A general practitioner can also do this"
- In my opinion, not necessarily, as there are also good physiotherapists, some of whom have a better insight into joint anatomy. They may not be able to carry out the examination themselves, but they could be consulted.
- "No, all professions that are qualified to do so should be involved (trainers, physiotherapists, sports scientists). Otherwise, this project will not be included in the care programme and too few people will benefit from such an examination!"

**Agreement, preference for this alternative ('A sports medical check-up should be carried out by a specialised doctor, preferably with certification in sports medicine.')**

- better
- Yes, because there are generally not enough sports physicians and it could be an inhibition threshold to go to a different doctor first than to simply have the examination carried out by the family doctor as part of normal check-ups/appointments
- Sounds plausible to have the additional qualification if possible
- Sports scientists or physiotherapists with additional training would also work in my opinion

**Preference for the other alternative ('A sports medical check-up should be carried out by a specialised doctor with certification in sports medicine.')**

- ... not just a name, they should take it seriously!
- B better
- Better variant B
- Better: additional qualification in sports medicine
- That sounds too larifari to me.
- No, not preferably but ONLY
- The examination only makes sense if the relevant expertise is available. The title/additional designation on the bell plate is of secondary importance if the patient-doctor relationship is right and the specialist knowledge is available. However, the additional qualification is a good indicator, especially for patients who want to have a sports medicine examination for the first time.
- Preference for alternative B - the wording "should" already covers the "preferably".
- Tend to favour recommendation 7, variant B.
- Preferably? Rather not

**Addition of further criteria to the recommendation**

- If the additional qualification in sports medicine is not available, other criteria should be determined to establish that the doctor in question is familiar with the field of sports medicine.

**Depending on the intensity of physical activity**

- Specialist knowledge should be sufficient for the initial examination for recreational sports, for more intensive strains / sports rather by sports physicians

- I think the designation and specialisation of sports medicine is particularly necessary when it comes to intensive sport (competitive sport with competitive ambitions) with a high level of training (> 4 times a week).

**Simple agreement**

- Yes
- Yes
- Yes
- Agree
- Agreement

**Simple disagreement**

- no

**Discussion of resources**

- This may not be possible, or only for private patients ... I was in the rehabilitation clinic with a doctor in training

**Unclear**

- Formulation "should" implies alternatives such as non-medical personnel who carry out the examinations
- Is sports medicine really just a "designation“

**7 Alternative B: A sports medical check-up should be carried out by a specialised doctor with certification in sports medicine.**

---

**Higher grade of recommendation**

- I think variant B is more clearly phrased. However, I feel that the "should" in both formulations leaves room for the doctor to carry out the examination without specialist knowledge. I wouldn't like that. Therefore, the following suggestion: "A sports medical check-up may only be carried out by a specialist doctor, preferably with an additional qualification in sports medicine."

**Agreement, preference for this alternative (‘A sports medical check-up should be carried out by a specialised doctor with certification in sports medicine.’)**

- ... not just a name, they should take it seriously!
- Definitely. If you are going to have a sports medicine check-up, then please get it from a doctor who specialises in this area.
- Better
- Better only from a sports physician! Such a complex topic requires more specialised knowledge. Incorrect instructions or recommendations can cause serious damage to your health.
- Better because people who do not have the additional qualification are not as suitable.
- This is probably better because hardly anything is taught about sports medicine in the degree programme itself.
- The additional qualification is a good indicator, but the quality of the examination is only really good if the doctor undergoes regular further training and thus maintains their specialist knowledge.
- The additional qualification is a sign of expertise and I wouldn't have it done by someone else
- I expect that only a sports physician is allowed to carry out a sports medical examination. After all, no internist is allowed to operate on a hip.
- It's more specific and not so vaguely worded by omitting "preferably". I think it's better.
- Yes, I think it makes more sense and appeals to me more if I can rely on the fact that only qualified sports physicians will carry out the examination.
- See previous variant (preference for variant B - the wording "should" already covers the "preferably").
- Would be the best alternative

**Preference for the other alternative (‘A sports medical check-up should be carried out by a specialised doctor, preferably with certification in sports medicine.’)**

- General practitioners should also be able to offer the examination.
- The additional qualification is required here, which is of course very bureaucratic again.
- I actually think that it is not absolutely necessary to have an additional qualification. After all, vital signs are also checked and everything else that prepares the body for sporting activities.
- In my opinion, not necessarily, as there are also good physiotherapists who sometimes have a better insight into joint anatomy. They may not be able to carry out the examination themselves, but they could be consulted.
- See above (sports scientists or physiotherapists with additional training would also work in my opinion)
- Too restrictive

**Simple agreement**

- Definitely
- Yes
- Yes
- Yes
- Yes!
- Agree
- Agree

**Simple disagreement**

- no

**Discussion of resources**

- Would be desirable but certainly not feasible given the shortage of doctors

**8 A standardised medical history form should be used as part of the sports medical check-up. This should include the following items:**
**• Personal and family medical history**
**• Individual risk profile**
**• Participation in earlier check-ups and their results**
**• Information on surgical procedures and injuries**
**• Nutritional history**
**• Gynaecological history**
**• Vaccination status**

---

**Important from a citizen’s point of view**

- "The gynaecological history should be taken with sensitivity. Women, especially middle-aged women, may have experienced difficult situations regarding certain questions in this area -> e.g. birth, miscarriage. It is good that the topic of gynaecological anamnesis is listed separately, alongside the personal anamnesis.

**Supplementary medical history questions**

- "1. intake of WADA[3]-listed products (today/past). 2. participation and results of previous medical check-ups --> supplement/concretise with performance tests (lactate, spiro, ...)"
- Possible further topics, if not already covered in the topic areas:- Current stress level and emotional stress
- Actually too little. More parameters are needed.

[3] World Anti Doping Agency

- Use of medication (influence)
- Mental state
- What is meant by gynaecological anamnesis?
- What is meant by gynaecological anamnesis? And does this mean that a gynaecological examination is carried out?
- Are mental illnesses and mental limitations recorded?

**Supplementary sports history questions**

- Previous experience in sport
- "The sports and exercise history is missing
- Question about what sport the person does/would like to do.
- Possibility of own assessment of own fitness status"

**Criticism about individual parameters**

- The question of participation in and results of other preventive examinations has only limited relevance here.
- There is no place in the sports anamnesis for asking about vaccination status."
- A general anamnesis of the family seems somewhat exaggerated. Then rather specific e.g. cardiovascular diseases.
- "Gynaecological anamnesis only in individual cases and only for specific questions, not in general (personal rights). What does the vaccination status have to do with it?"

**Depending on the sport**

- Doesn't that also depend on what sport someone wants to do? Is it different for jogging and yoga than for football or boxing, for example?
- if necessary

**To be clarified in the text**

- What does the term "individual" risk profile mean here, example?

**Proposed editorial changes**

- "Suggested changes to highlighted words: *Questionnaire on personal medical history *Gynaecological medical history"

**Agreement, encouragement**

- This definitely increases the comparability of the results should the patient be forced to change doctors.
- For me, this is part of an examination
- Very important, the woman's cycle and general gynaecological aspects should always be included in examinations. Aspects should always be included in examinations, especially in sports medical check-ups.
- Gynaecological anamnesis is very important! Cycle-adapted training was completely neglected for many years and has only recently been used in professional sport. More and more studies are currently being conducted on this topic. Bone marrow oedema is also favoured by hormonal irregularities.
- gynaecological anamnesis reasonably only in women. Otherwise, there are many gynaecological aspects to consider, so I find this very good (pregnancies, postmenopausal, taking hormones, amenorrhoea etc.). Many aspects are specific to women.

**Simple agreement**

- Covers all areas👍
- Good, everything understandable
- Yes
- Yes

- Yes, makes sense
- Sounds sensible to me.
- Nothing to complain about
- Fits, no objections
- Agree
- Agree
- Absolutely necessary

**Simple disagreement**

- no

| From the citizens' perspective. |
| --- |
| • Sensitivity is particularly important when taking a gynaecological history, as some women may have experienced difficult situations. |

**9 A validated score should be used to assess the cardiovascular risk.**

**Important from a citizen's point of view**

- Here, too, it should be noted that results from scores can be unsettling or do not match the respondent's own assessment, which can be stressful for the respondent.
- This can be useful, but should be supplemented by an individual, subjective assessment.
- As long as the determination of the score and the categorisation is easy to understand, it is certainly a useful tool for determining the current situation. However, as every person is different, personal counselling by the doctor should not be neglected.
- Possible, but should always be determined individually on the patient and take individual factors into account, score not always meaningful

**Specification of the recommended score**

- It depends on the criteria. Not every score makes sense...
- Okay, but currently not assessable as long as there are no proposals for scores.
- I can't imagine that such a value can be found and that it is meaningful.

**To be clarified in the text**

- Sounds good, but is no substitute for a professional assessment by a person with expertise. I can already see a self-test...
- What goes into this score? That should be explained.
- What is the score based on? Without a connection to the examination, it is not clear to me what this is supposed to mean.

**Possible indications for the evaluation of cardiovascular risk**

- Should only be evaluated in case of previous illnesses or family history is positive

**Proposed editorial changes**

- If I were you, I would translate "validated score" or phrase it differently.
- Is it clear to everyone what validated means?
- What is a validated score (in layman's terms, please)?
- What is a validated score?

**Simple agreement**

- +
- I think it's good
- Good idea

- Yes
- yes
- yes
- yes
- Yes!
- Yes!
- Yes, sounds plausible.
- Makes sense
- Agree
- E.g. score, yes
- Agreement

> From the citizens' perspective.
> - When communicating results from scores, care should be taken to respond to the subjective assessment, to take into account possible uncertainty or stress for the person being examined and to follow up with personal counselling.

**10 A sports history should include questions about**
**• the frequency, intensity, duration and types of sport**
**• previous experience with sport**
**• Environmental conditions during sport such as heat, cold or altitude**
**• complaints at rest and during exercise**
**• individual sport-related risk profile and**
**• any aids or restrictions (e.g. visual aids).**

---

**Important from a citizen's point of view**

- "Maybe how important sport is for your general well-being. Background: When I have orthopaedic problems, doctors often tell me not to do any sport for the next few weeks/months. But it's really hard on my psyche. I need to do a certain amount of sport to relieve tension and cope with everyday life."
- Sports medicine counselling is particularly important for people with serious pre-existing conditions and severe disabilities so that they can choose a suitable type of sport. Clubs and sports centres that allow inclusion are difficult to find.
- Knowledge of the effects of sport (cardiovascular system, hormonal balance, mobility, etc.) in terms of motivating people to exercise.

**Supplementary sports history questions**

- Occupational physical activities
- Inquiry about post-exertional malaise (PEM). If PEM is present, then classification of the severity or enquiry of the "stress limit"
- Physical activities in leisure time (e.g. gardening, housework)
- Isn't the goal and training plan missing here?
- I am missing the (non-medical) question about (own) knowledge of risks during sport. E.g. doing sport while chewing gum = life-threatening, which strictly speaking could be categorised as previous sporting experience. However, I often see people chewing gum, just like people wearing finger rings (high risk of injury), or chains on the neck during sport, earrings are conditionally dangerous.
- Motivation, fears
- Knowledge of the sport => does the person know how to do the exercises correctly? Are they instructed or do they intend to do sport alone => higher risk of injury

**Supplementary medical history questions**

- When taking a sports history for people with insulin-dependent diabetes, a much more detailed history of diabetological complication rates and previous risk management should be taken.
- "Supplements: - menstrual cycle - family history of illnesses - diet - sleep (recovery phases) - organisation of training breaks (recovery)"

- "Nutritional habits
- Question about the use of performance-enhancing drugs, and question about regular intake and dosage of dietary supplements
- See the comment on WADA-listed aids.

**Agreement, encouragement**

- definitely, from my own experience everything is important (camping at -20°C at Everest Base Camp is different from hiking in the Odenwald).
- For me, this is part of the medical history
- Yes, only then can a training recommendation make sense.

**Simple agreement**

- All understandable.
- Also sensible
- yes
- Yes
- Yes
- Yes
- Yes exactly
- Yes of course. Belongs to it
- Yes very good
- YES
- YES
- YES"
- Sounds good
- Sounds good.
- ok
- Very good!
- Makes sense
- Agree
- Agree
- Absolutely
- Agreement

| From the citizens' perspective. |
| --- |
| • Citizens emphasise the importance of asking about the individual motivation of the person being examined for sporting activity and strengthening this motivation through discussion. Individual obstacles should be taken into account and reduced through appropriate counselling. |

**11 Obtain a whole-body assessment as part of the sports medical check-up:**
**• Collection of anthropometric data (height, weight, abdominal girth, calculation of body mass index)**
**• Pulse and blood pressure measurement at rest**
**• Examination of heart and lungs**
**• Assessment of vascular status**
**• Abdominal examination**
**• Examination of the musculoskeletal system including a) mobility and stability of the large joints b) mobility and tapping pain of the spine c) muscle status / function, tone, etc. Examination of the nervous system (gait pattern, reflex status, sensitivity, cranial nerve status if applicable)**
**• Examination of the sensory organs (incl. visual acuity assessment with eye charts)**
**• Inspection of the skin**
**• Inspection of the oral cavity, recording of dental status**
**• Assessment of lymph node status**

---

**Selection of parameters depending on intensity of physical activity**

- Also appears to be very extensive. Should be more in relation to recommendation 10, i.e. adapted to the sports organisation.
- Here I would adapt the scope of the examination to the athlete's profile. An amateur athlete does not have the same needs as a competitive athlete.
- This definitely makes sense for an initial examination. Depending on the frequency of the repeat examination, the scope can be reduced or extended depending on risk factors (previous) illnesses/injuries and the level of sporting activity, whereby the full scope should be checked at least every second examination. Additional examinations such as exercise ECG or spiroergometry should also be carried out depending on the patient and type of sport. The scope described above should therefore serve as a minimum standard and not be set in stone.

**Suggestions for additional tests**

- "Could the BMI be replaced/supplemented by more helpful parameters, e.g. body fat percentage? Otherwise useful"
- "Urinalysis, rule out urinary tract infection. Bladder ultrasound, exclude residual urine formation. Incontinence?"
- In addition, I would suggest asking about the type of diet (vegan, vegetarian, mixed diet, etc.)
- Collection of anthropometric data (height, weight, waist circumference, calculation of body mass index) --> add fat percentage
- Hand strength measurement
- Include sonography

**Criticism about individual parameters**

- BMI is sometimes misleading for athletic/very muscular people
- BMI does not matter, then rather really body fat
- BMI is only partially meaningful
- As far as I know, the BMI is now criticised by many experts because it does not take into account the fact that fat people can also have a lot or little muscle mass, which has a major influence on fitness and the risk of injury.
- But this is expensive and time-consuming. Teeth ? What for? But you should check how the pelvis is positioned and possibly send it to an osteopath, orthopaedists usually don't pay any attention to it and incorrect posture impairs running performance, for example, because the hips are then not mobile.
- "-The abdominal examination is not clear to me - with what intention? - How should the vascular status be assessed? Invasively? Or as a visual diagnosis? - Of course, a healthy oral cavity status is important - however, it would possibly be sufficient if this examination, for example, were carried out additionally and not as standard per se"
- The lymph node status survey is unclear to me.
- Seems a bit exaggerated, e.g. dental status.

- "Why should the mouth area be examined during a sports medicine check-up? Why an inspection of the skin?
- It seems to me that the inspection of the oral cavity may be somewhat superfluous, depending on the type of sport involved in the check-up.
- I find the dental status and sensory organs too much in the context of sport.

**To be clarified in the text**

- "I don't understand why the dental status should be important. What significance should the skin have for a sports examination? It's unclear to me! Should the lymph node status be standard, or only if the risk factors are present?"
- Sensible, but needs to be differentiated
- Overlap with GP check-up

**Agreement, encouragement**

- Absolutely necessary and very feasible
- For me, this is part of the examination...
- Simple agreement
- Recommendation is acceptable and understandable
- Yes
- Clear
- None
- Sounds plausible
- Okay
- Okay
- very good
- super
- Super!

**Disagreement, with reasons**

- These examinations are not obvious to me and I also consider them unnecessary unless the patient has a special medical history or type of sport (e.g. boxing--> dental examination) "

**Simple disagreement**

- no

**12 Consider measuring muscle strength as part of the sports medical check-up.**

**Important from a citizen's point of view**

- I find that particularly interesting. For me, these would be values that I could use to determine a significant increase. It may also be motivating in the course of the treatment.
- It would be great to make changes visible to patients during a subsequent examination and to give them a feeling for their current condition so that they can start any strength training in a sensible way
- For what purpose? May be helpful to explain.

**Possible indications for measuring muscle strength**

- This should always be done during an initial examination or after an injury to the musculoskeletal system in order to avoid imbalances. Repeat examinations can also be carried out in longer cycles (every 2nd, 3rd or 4th repeat examination) provided there are no medical indications to the contrary.
- This could be useful to recognise the differences before the start of sporting activity and at a certain later point in time. In my opinion, however, it is particularly useful for older patients.
- This is necessary for training control, but does not necessarily have to take place during the sports medical check-up. However, this does not necessarily have to take place in the sports medical check-up.

It's more a question of whether the symmetry of the body allows for the sports units or whether misalignments can cause damage.

- I would simply advise muscle training, well measuring for old and sick people perhaps

**To be clarified in the text**

- The recommendation may leave room for manoeuvre. Which muscles are meant here and which procedure can be used?
- I only consider this to be useful to a limited extent, as the reason or the findings from this are not clear to me.
- Without context, it is not clear to me how and why this should be done.
- Why?
- What would be the reason/goal for such an examination without reasonable suspicion?
- Which muscles? Doesn't necessarily make sense for every sport.
- For what purpose?

**Proposed editorial changes**

- Hand strength measurement

**Strength of the recommendation**

- "Can"? Thought it was a binding guideline?
- I think it should be done!
- Should it absolutely be done
- should, not can
- should?
- If it "can" be done, but doesn't have to or should (and possibly won't as a rule), you can also leave out the wording.

**Agreement, as a fee-based option**

- The measurement should be offered as optional.
- Individual - possibly add-on service
- Useful, but not as part of the "basic package" --> add-on service.

**Simple agreement**

- ✓
- Please!
- with pleasure!
- Yes
- Yes gladly
- Okay
- Okay
- Great!
- Absolutely!

From the citizens' perspective.

- The purpose of measuring muscle strength should be explained.
- Measuring muscle strength can be perceived as motivating for further training if changes are made visible.

**13 A full blood count, pre-prandial blood glucose concentration and lipid status, liver/kidney values, electrolyte concentration and urine status can also be determined if necessary.**

**Important from a citizen's point of view**

- It's very good if you talk about it afterwards, if you get it. Because a "yes, it looks good" is not helpful.

**Suggestions for additional parameters**

- also well suited, lipometabolic disorders should also be investigated (GPTI/II deficiency)
- Here too, people with insulin-dependent diabetes should refer to the available CGM[4] data, as they feel they are being made fun of with a preprandial BG value.
- Blood sugar seems sensible to me, but then together with the progression during sporting activity.
- A complete blood count to recognise deficiency
- Measure vitamin D levels
- Why not a full and differential blood count?

**Strength of the recommendation**

- For me, this would be standard and not optional only if required
- Useful in the basic package.
- I wouldn't do this as a supplement, but as standard, so that nothing is overlooked that could be relevant for sporting activity, such as diabetes mellitus, which has not yet been recognized.

**Agreement, encouragement**

- Desirable as a supplement to recommendation 11
- Essential at a higher level
- Always useful for an initial examination and in the presence of corresponding risk factors to determine the current situation. Repeat examinations can be dispensed with if there are no other medical indicators or if deviations were identified as part of the general medical check-up or during the initial examination.
- These examinations should only be carried out or offered if there are actual indications of illnesses or similar. The values would be known to me, for example, and should not be taken again.
- A small blood count makes sense. The rest only on request.
- Yes, then every 3 years if a check-up is planned anyway
- You should also have something like this examined independently of sport
- Should be done; also to make before/after changes visible. And: to find out whether certain types of sport are not suitable.
- Top, certainly an advantage for competitive athletes, for example
- "Important to counteract blood sugar derailment under stress. Electrolytes and retention parameters for assessing fluid intake, electrolyte replacement under stress/sweating. Recognize the risks of protein supplementation during muscle training and inform the patient."

**Simple agreement**

- +
- ✅
- The recommendation is clearly phrased.
- I think it's good
- good
- yes
- OK
- Okay
- Okay
- Fits

[4] continuous glucose monitoring

- reasonable
- Agree
- Would make sense.

| From the citizens' perspective. |
| --- |
| • A discussion and handover of the results is requested. |

**14 Other parameters, e.g. inflammatory markers, renal retention values, should be determined if there is a corresponding clinical suspicion.**

**To be clarified in the text**

- In my opinion, due to the suspicion, this goes beyond a basic sports medicine examination and would be a specialist clarification --> referral
- Yes, but not in connection with a sports medicine examination, but rather during a general examination, e.g. at the GP.
- YES, absolutely, but please define in more detail.
- Where should the clinical suspicion come from? Shouldn't a recommendation for treatment be made to look out for signs of such illnesses?

**Possible indications for when to determine further parameters**

- only in case of clinical suspicion or e.g. conspicuous U-Stix
- or in case of previous illness

**Agreement, encouragement**

- Useful in case of clinical suspicion.
- Yes, inflammation markers are important in case of a history of injury

**Simple agreement**

- +
- ✅
- Absolutely.
- Absolutely.
- Recommendation is clearly formulated.
- Yes
- yes
- Yes
- Yes gladly
- Yes.
- Yes Okay
- Okay
- Fits
- Agree
- great

**Disagreement, with reasons, preference for additional testing**

- Essential at a higher level
- In any case to avoid possible secondary diseases.
- Should be done; also to make before/after changes visible. And: to find out whether certain sports are not suitable.

**Simple disagreement**

- no

**Unclear**

- I can’t say

**15 A 12-lead resting ECG should be recorded as part of the sports medical check-up.**

**Suggested indications for a 12-lead resting ECG**

- Essential at a higher level
- Certainly useful for performance-intensive sports and sports that require a high level of endurance (e.g. triathlon)
- "You could do it for people under 30, but especially if you have a conspicuous medical history
- for over 30s or over 40s at the latest, it should be a must"
- Yes, definitely useful if you do endurance sports.

**To be clarified in the text**

- Why is the ECG important?

**Strength of the recommendation**

- A must!!!

**Agreement, encouragement**

- Recommended, as abnormalities in the heart rhythm can be detected here.
- I think this is very good, as it is important to know whether the heart is also healthy at rest and this data can then be compared with that during exercise.
- Random findings would be possible here. If, that is, a qualified specialist evaluates them. I'm interested to know if everything is okay.
- I would do a stress ECG

**Simple agreement**

- +
- In any case
- In any case.
- Yes
- Yes gladly
- Clearly formulated
- Comprehensible.
- Okay
- Okay
- Plausible
- Very sensible
- Agree
- Absolutely
- Absolutely!
- Important!

**Disagreement, with reasons**

- I don't know, an athlete should rather be measured during physical exertion.

**Simple disagreement**

- Not useful

**16 Do not routinely perform echocardiography as part of the sports medical check-up.**

**To be clarified in the text**

- Define exceptions (e.g. shortness of breath/lung disease/hypertension/dysrhythmia, abnormal ECG, etc.).
- exactly, but when then?
- Brief explanation
- When should it be carried out? Only if...?
- Why not?

**Suggested indications for echocardiography**

- This may be useful for sports that put a lot of strain on the heart.
- Echo only if cardiac disease is known or suspected (e.g. noise on auscultation)
- Only anamnestic if there is a pre-existing condition
- As long as the ECG is normal, I agree with this recommendation. However, if the ECG shows abnormalities, an echocardiogram should be performed.
- But only if there is a pre-existing condition that makes it necessary. So only if there is an indication. I don't think it makes routine sense.
- If the ECG is abnormal, I would recommend it to get to the bottom of the cause.
- I would only recommend it if the findings for #15 were abnormal

**Agreement, encouragement**

- Yes, should only be done if indicated
- If there are corresponding indicators of necessity, this should be done. If corresponding indicators are found during the screening examination, a referral to a cardiologist for clarification should be made in any case. These results should then be taken into account in any subsequent repeat examinations.

**Simple agreement**

- +
- yes
- yes
- Yes gladly
- Yes okay
- Fits
- Sensible
- Agree

**Disagreement, preference for routine echocardiography**

- Yes, at a high level in any case
- that would be a good thing, especially for competitive athletes. HOCM doesn't cause any symptoms early on...
- Personally, I would like to see an ECHO as a matter of routine!
- Contradiction, should be carried out

**Simple disagreement**

- I cannot support this

**Unclear**

- No opinion
- Not useful at all.

**17 An exercise ECG should be recorded as part of the sports medical check-up if there is an increased cardiovascular risk and/or exercise-induced symptoms.**

**Preference for a stress ECG even in the absence of increased risks**

- Essential even at a higher level without risk
- I would always have expected that.
- An exercise ECG should be part of every sports medical examination, as the primary aim is to rule out risks during sport. Possible problems / risks can only be ruled out during exercise.
- A stress test ECG should always be carried out.
- For me, this is part of the routine examination
- I would like to have it even without symptoms, it would give me more confidence to push myself to my limits if I know that my heart is healthy
- I would like an exercise ECG to be carried out even if you suffer from partial extrasystoles, for example.
- Always do it
- Always! And not only when symptoms are present. That's the point of sport, that the heart is "stressed".
- Yes, not only if, but in principle; exercise ECG is the only examination that comes close to the future activity (sport).
- yes, but perhaps also in principle, as cardiovascular risks may only be identified in the process
- Makes sense in general in my opinion, not just if there is a known risk.
- In my opinion, this should always be carried out, as any illnesses/deviations can only occur during exercise.
- I would want to do this even without an increased c risk

**Simple agreement**

- +
- In any case
- In any case
- in any case yes
- yes
- yes
- yes
- Yes gladly
- Yes!
- Yes, with pleasure!
- Yes.
- Okay
- Fits
- Very useful
- Sensible
- Agree

**18 Consider using the results of the exercise test to determine cardiorespiratory fitness and to derive training recommendations.**

**Shared decision-making**

- If necessary or requested by the patient, this should always be done. At the very least, however, possible risks due to incorrect training should be pointed out.

**Proposed editorial changes**

- Couldn't you simply bundle 15-17 under "cardiorespiratory fitness is suitable" and then define "suitable"? This should be a main goal in order to be able to set realistic training plans.

**Agreement, encouragement**

- Essential at a higher level
- That's exactly what you want to know
- Very good! Also important for healthy patients!

**Simple agreement**

- +
- in any case
- Yes
- Yes
- yes
- yes
- Yes
- Yes!
- Yes.
- Yes, please!
- Yes, please!
- ok
- Okay
- Fits
- Very good
- Very good
- Very useful
- Sensible
- Agree
- Super!
- absolutely
- absolutely!

**19 Spiroergometry can be part of the sports medical check-up to determine fitness and for training recommendations or monitoring.**

---

**Important from a citizen's point of view**

- "That would be a great incentive for participation!
- This way, athletes get a real benefit from the examination (apart from not being diagnosed with a disease)"

**Suggested indications for spiroergometry**

- Essential at a higher level
- In any case. Spiroergometry should always be included in the initial examination and in the case of high levels of physical exertion or existing risk factors / relevant pre-existing conditions. Otherwise, it can also be offered as an option.
- Supplement: if the sporting goals justify it or if sporting goals go beyond the hobby level.
- Can be done, but does not have to be. Depends on the age of the patient.
- Can, especially if intensive sport is to be practiced and a training plan can be derived from this
- Only with previous stress
- Should definitely be done in the case of pulmonary preconditions

**Shared decision-making**

- Yes , runners want that

**To be clarified in the text**

- add-on service.

- Under what circumstances?

**Strength of the recommendation**

- can -> should!
- As above: I find "can" too imprecise.

**Agreement, encouragement**

- Also very good, as not only the heart but also the lungs are examined.
- Makes perfect sense in my opinion. These values would interest me.

**Simple agreement**

- I think it's good
- I think it's good!
- Yes
- Yes
- Yes
- Yes
- Yes with pleasure
- Fits
- Very good
- Very good
- Very good idea
- Very useful
- Sensible

**Disagreement, with reasons**

- Very interesting, but perhaps a little over the top?!

**Simple disagreement**

- no

From the citizens' perspective.
- Spiroergometry can be an incentive for undergoing a sports medical check-up.

**20 Carry out additional laboratory and instrumental examinations only in individually justified cases.**

**Preference for preventive spiroergometry**

- In my opinion, this (spiroergometry) should be carried out on everyone, so that an overview can be measured not only medically/theoretically, but also in the direct situation during sport, as complaints can only become apparent during exertion.
- No, also as a preventive measure (spiroergometry)
- No, also essential at a higher level (spiroergometry)

**Suggestions for additional testing**

- Abdominal ultrasound should be standard
- Radiation exposure should never be used provisionally, but MRI examinations in case of suspicion would be a good idea

**To be clarified in the text**

- What does instrumental mean in this case?

**Proposed editorial changes**

- Could this not be worded more clearly? "[...] should not be carried out." In justified individual cases, it is possible to deviate from the guideline anyway.
- Too vague.

**Agreement, encouragement**

- Yes, if I have any anamnestic evidence or conspicuous values, it would be interesting for me.
- Yes – as an individual health service
- Yes, really only in justified individual cases
- Makes sense in any case, also to limit the effort involved.

**Simple agreement**

- +
- I think it's good enough
- good!
- Yes
- Yes
- Yes
- Yes
- Yes
- Yes!
- Yes!
- Makes sense!
- Okay
- Okay
- Sensible
- Sensible
- Agree
- Agree

**21 Swollen joints, pain, relevant movement restrictions or instability of large joints as well as motion pain or pain on percussion of the spine with or without neurological deficits should be referred for specialist evaluation (specialist in orthopaedics/trauma surgery or physical and rehabilitative medicine).**

**Shared decision-making**

- Yes, a referral should be made in any case. Whether this is then accepted by the patient or not is up to them.

**To be clarified in the text**

- ... before, after, instead of a sports medical examination?
- "Type of pain?
- Physiotherapeutic assessments should also be given more consideration here

**Proposed editorial changes**

- "neurological deficits" could be formulated even more simply.
- Another word for referred - presented?

**Agreement, encouragement**

- I also think it makes sense. The sports physician can't do everything on their own.

**Simple agreement**

- +

- ✓
- Definitely
- Yes
- Yes
- Yes
- No proposed changes
- Clearly understandable, no proposed changes.
- ok
- Okay
- Fits
- Very useful.
- I agree
- Agree

**Disagreement, with reasons**

- so I would recommend cooling first and then sports physiotherapy, why a doctor straight away? (except for neurological deficits) and of course depending on the duration of the pain and the extent of the movement restrictions, but also far too vague a recommendation and not focusing on the person affected and their health competence
- I thought that sports physicians should also be familiar with orthopaedics, at least to a limited extent. Isn't this therefore a matter of judgment?"
- No, too much unnecessary clarification; only if there is a suspicion of illness
- only if these complaints/symptoms have not already been clarified beforehand. Many people have back pain that was previously treated by a GP when it was acute. They don't all have to see an orthopaedist now just because they are being investigated by a sports physician (e.g. for cardiovascular reasons). What should they do?
- Shouldn't physiotherapy be recommended here?

**Unclear**

- That would make sense regardless of a preliminary sports medical examination?
- This should be decided by the doctor responsible for the sports medical check-up.

**22 People with artificial joints or a history of serious injury (e.g. vertebral body fractures, recurrent joint injuries) should be monitored regularly by a specialist.**

**To be clarified in the text**

- If applicable, time specifications for regular (e.g. in addition to the sports medical check-up at an interval of XY)
- With what goal and what does regular mean?

**Proposed editorial changes**

- "recurrent" could be formulated even more simply.
- Instead of should, "is recommended "

**Agreement, encouragement bzw. Ergänzung**

- This is absolutely necessary in order to prevent/avoid complications.
- Not by sports physicians

**Simple agreement**

- +
- ✓
- Makes sense
- in this case yes

- Yes
- Yes
- Yes!
- Yes!
- Yes!
- No proposed changes
- Clearly understandable, no suggestions for changes Clearly understandable, no suggestions for changes.
- ok
- Okay
- Anyway
- Agree

**Disagreement, with reasons**

- This should also be done independently.
- Here, too, I don't see the support as being provided by specialists, but rather in the promotion of people's health literacy and in physiotherapy, especially what is meant by regular and why check-ups without complaints and restrictions - not exactly in terms of health economics
- Aren't patients being monitored by specialists anyway?
- Aren't they anyway? What is the additional benefit of the preliminary examination in these cases?

**Simple disagreement**

- no, not regularly

**Unclear**

- no opinion

**23 Risk to self and others and possible aggravation of previous injuries should be considered when assessing sports clearance.**

**Important from a citizen's point of view**

- Climbing (rope team), gliding, etc. certainly pose a risk to others, but jogging, strength training on equipment and Zumba are rather unlikely. The risk of self-endangerment should be communicated to patients, but in my opinion they should not be frightened and discouraged from doing sport.
- However, I would be careful with the term "risk to others", as this could also lead to "bans"."

**Shared decision-making**

- In any case, ultimately it is up to the athlete to decide

**To be clarified in the text**

- Risk to self and others
- How should this be assessed, what questionnaires or other tools are available??

**Further aspects of risk assessment**

- I think assessment in the sense of assessments is good, the more important question in terms of health literacy is how the person concerned understands this information and what they do with it.
- "Elite athletes in particular are already quite well positioned in this area thanks to their team of physios and doctors. Patients who are already restricted should not do any sports that are not possible due to their illness."

**Proposed editorial changes**

- " Risk to self and others" could be expressed even more simply.

**Agreement, encouragement**

- Yes, otherwise the examination and the training recommendations ideally derived from it make no sense.
- "If that didn't happen, the whole preliminary examination would be somewhat superfluous?

**Simple agreement**

- +
- ✓
- Good
- Yes
- Yes
- Yes
- Yes
- Yes
- Yes
- Yes as far as predictable
- Yes!
- Yes, sounds sensible
- No suggestions for change
- Clearly understandable, no suggestions for change.
- ok
- Agree

From the citizens' perspective.

- A written report for reference is requested.
- When communicating the risk of danger to oneself and others, care must be taken to ensure that the person being examined is not made insecure and discouraged from doing sport.

Information needs of consumers

- a list of available sports physicians in the vicinity

## Appendix 4. Comments on draft recommendations from the physicians' perspective

All text was translated with www.DeepL.com/Translator (free version) with manual modifications. We counted comments conveying agreement ('yes', 'ok', 'agree', etc.) and disagreement ('no, 'disagree', etc.).

**1 Alternative A: "Offer a sports medical check-up to adults who want to take up or return to sport" or alternative B: "Offer a sports medical check-up to adults who do sport."**

- Sports physicians: n=49 preferred (A), n=44 (B), n=55 both (separately or as joint recommendation), n=1 neither of the two
- Other specialists: n=3 preferred (A), n=6 (B), n=6 both, n=1 neither of the two (generally against the examination)

**Reasons against alternative A ('Offer a sports medical check-up to adults who want to take up or return to sport')**

- the question arises as to whether the general public should bear the costs of years of inactivity
- everyone who engages in a sporting activity should be informed about its risks and limitations

**Reasons against alternative B ('Offer a sports medical check-up to adults who do sport.')**

- much leeway with regard to indication
- too generalized
- too specific
- it is certainly not possible to examine everyone
- as an individual health service, otherwise not feasible
- exaggerated
- overvalued
- cost issue that not everyone should be offered it otherwise the very ambitious rather than the medically endangered are more likely to use it.

**Reasons against both (Sports physicians)**

- is sport/exercise really dangerous or just money making

**Reasons against both (other specialists)**

- Overuse/misuse: Adults who are active in recreational sports will rarely find themselves get into extreme stress situations. Minor exertion is well tolerated in most cases. And: people are led to believe that preventive care anticipates emerging problems that the patient would not notice himself and this will lead to incapacitation

**Offering character**

- Offer yes, but no compulsion
- The threshold is too low under B or could even induce avoidance of sport
- Exercise should not generally be tied to a medical examination at the customer's request; clearly an offering nature
- whereby an unnecessary hurdle can arise to first organize a sports medical examination, which is not available everywhere, if the adult receives a recommendation from their doctor to exercise more

**Depending on age**

- People who do sport from a certain age
- restricted to adults > 40 years; age >40 years
- Insert age specification, e.g. over 35 years, as the risk of sport in the 18 to 35 age group is very low according to current literature

- specify: From a certain age?; it makes sense to differentiate from what age; especially at an older age; possibly address age; possibly with age structure categories

**Depending on the intensity**

- especially if the performance limit is reached in training or competitions; if competitive sport
- Offer with full scope of examination only makes sense and is advisable for competitive sports/risk sports or special individual risks
- Sport, to what extent, strain and the scope of the examination depending on this scope of the examination
- if the client demands a higher level of performance

**Depending on risk**

- for recreational athletes depending on risk profile and sporting activity
- for risk-defined pre-existing conditions; for pre-existing conditions/risk factors; only for pre-existing conditions
- Offer with full scope of examination only makes sense and is advisable for competitive sports/risk sports or special individual risks
- restricted to adults > 40 years and younger who have a cardiovascular risk
- Provide regular sports medical care for athletes with e.g. CHD
- specify: All adults or healthy adults? When do they want to start exercising again after / with cardiac / oncological / orthopaedic disease?
- Especially in case of medication intake or pre-existing conditions
- if illnesses or restrictions are present; especially in case of illness or medication intake

**Specify the recommendation**

- Unclear wording: What does " want to return to sport " mean? How should the sports break be, before recommending a sports medical check-up? How is "sport" defined based on (i.e. which intensity, frequency etc.)?
- Define sport a little more: Hiking, dancing, walking are often not defined as sport. Many people only understand sport to mean running, swimming, cycling
- at regular intervals

**Specific comments to alternative B (‘Offer a sports medical check-up to adults who do sport.’)**

- How often? "Athletes" with 2-5-yearly rotations and possibly a reduced scope of examination. In my experience, you always find abnormalities here that have gone unnoticed so far, this may be a change in vision/hearing, missed vaccinations and much more.
- Alternative B, as it also includes athletes who play sport for a long time. These athletes may also develop health problems over time that should be recognized; even those who are active in sports get older and develop complaints and illnesses or feel unsafe
- change to ambitious and, in particular, very extensive and competitive sport
- only in case of specific complaints?
- as individual health service, otherwise not feasible; unrealistic, no capacity for everyone

**Financing**

- makes sense but what is about financing
- possibly offered in addition to the health check-up, depending on the amount of sport, and at best also remunerated
- the resources will not be sufficient in 5 to 10 years. It should be made clear in some way that this recommendation is not financed by the solidarity-based community for healthy people with no complaints
- in the outpatient setting, this would have to be paid by the statutory health insurance (variant B)

**Suggested wording**

- Alternative A, "can" for Alternative B
- All adults who (want to) engage in sporting activities should be offered sports medicine advice.
- "who want to do sport or take up sport again...."
- Adults who are taking up or returning to sport and adults who take up sport regularly should be offered a....
- Adults who want to take up sport should be given a...
- Adults who wish to take part in sport or who wish to take up sport again should be offered a sports medical check-up.
- Adults who play sport should be offered a regular sports medical check-up.
- Alternative B could be added as "Adults who regularly engage in intensive sport and/or take part in competitions should also be offered a sports medical check-up.
- Adults who take part in sport, or especially those who are new to sport or want to take it up again, should…

**2 Alternative A: "Use the relevant guidelines for people with chronic diseases." or alternative B: "Use the relevant guidelines for people with diseases."**

- Sports physicians: n=50 preferred (A), n=56 (B), n=15 both, n=12 neither of the two
- Other specialists: n=2 preferred (A), n=8 (B), n=2 both, n=2 neither of the two

**Reasons against alternative A ('Use the relevant guidelines for people with chronic diseases.')**

- a newly occurring hypertension would not be covered

**Reasons against alternative B ('Use the relevant guidelines for people with diseases.')**

- If this makes the rounds, the flood of inquiries will increase

**Recommendation to general**

- Both alternatives too "general"
- far too vague
- too undifferentiated
- far too general
- the respective guidelines should always be used for all chronic diseases, the respective guidelines should always be applied. because this is self-evident, this sentence can be removed
- which guidelines? who even knows the guidelines?

**Other criticism**

- Diseases are covered by statutory health insurance
- ....and then no sports medical examination? In my opinion, this specific examination is not included in the general guidelines for chronic illnesses so that it should be offered depending on the intensity of the sport.

**Reference to sport**

- the reference to sport must be included
- the guideline is ultimately for colleagues
- often do not address sport
- Where is the reference to sports medicine?
- However, it should be checked to what extent sports medicine is referred to in the guidelines for individual diseases

**Additions**

- with and after serious or chronic illnesses
- Add the word "injuries" to diseases

- acute (e.g. tumour) illnesses must also be included
- the term "chronic" may not include all relevant illnesses. For example, illnesses with recurrent symptoms are not necessarily considered chronic. There are also significant one-off events such as syncope
- Lack of reference to acute illnesses, e.g. infections (see COVID-19 and return to sport)

**Individualization**

- but individual advice necessary due to e.g. prior knowledge about performance
- but individual adaption
- I think that care tailored precisely to the patient group is good and important

**Restrictions**

- The type of disease (chronic/severe) must be restricted

**Acutely ill persons**

- In the case of acute illnesses, it would be the responsibility of any doctor to indicate a sports ban or restrictions, depending on the severity of the illness.
- B is too general, could cause a flood of examinations
- In my opinion, looking at the guidelines of specialist societies primarily, they concern chronic diseases. It creates an additional effort if the omission of the restriction "chronic" explicitly drills the sports physician to also look at the respective guidelines for acute diseases
- Acute illnesses that are a contraindication to exercise should be defined in the DGSP guideline
- Acute illnesses are also sometimes provided with guideline recommendations (e.g. myocarditis). acute diseases must also be taken into account, such as myocarditis

**Chronically ill persons**

- In the presence of chronic illnesses and sport, close examinations should be carried out, e.g. once a year, always based on the individual cardiovascular risk
- Particularly in the case of chronic illnesses, the specialist aspect dominates. For example, guidelines for the treatment of chronic heart failure, only when the specialist guidelines have been implemented can the sports medicine guidelines be applied in addition.
- sports medicine guidelines can be applied in addition
- What is chronic and what is not?; not every chronically ill person is impaired

**Clarity**

- Unclear. What does this refer to? To the chronic disease, e.g. the guideline for CHD or asthma, or to the sports medicine guideline?
- Which guidelines; which ones are there?
- Offer an example in brackets
- Unclear - sportmed guidelines? Internal medicine?
- Who actually needs individual guidelines for chronic diseases? Most people have various chronic diseases! And now ? I don't understand such recommendations

**Misunderstanding of the wording**

- What about people without known pre-existing conditions? These would be omitted in this formulation. People with illnesses certainly require special attention, but not only for them
- It's not just about chronic illnesses
- Chronic alone is too restrictive
- Not only chronic patients do sports
- The respective LL should also ....

**Suggested wording**

- Persons with illnesses that could lead to a restriction in their ability to participate in sport should be examined in accordance with the guideline.
- For persons with chronic diseases, the current guidelines issued by the relevant professional societies should be applied.
- The respective guidelines SHALL be used for persons with diseases..
- For persons with specific diseases (e.g. COPD, myocarditis) or pregnancy, the relevant guidelines SHOULD be applied.
- The relevant guidelines should be used for people with illnesses.

**3 A sports medical check-up should be conducted when starting a more intensive sports and exercise programme.**

- Sports physicians: n=89 positive, n=12 positive with modifications, n=11 negative, n=11 unclear, n=64 without comment
- Other specialists: n=10 positive, n=2 positive with modifications, n=1 negative, n= 11 unclear, n=5 without comment

**Restrictions**

- Depending on medical history and previous illnesses, especially for competitive sports, marathons etc.
- only an offer, not a “must do”
- I rather think that this should be recommended once a year and, if necessary, in the case of special features, as mentioned here, several annual examinations are not a BASIC EXAMINATIONS, which should also retain this character
- optionally yes; based on the cardiovascular risk
- Yes, but only as an individual health service
- Can be useful for endurance sports
- Not mandatory, depending on sports history; however, the offer should be available for those who want it

**Specify the recommendation**

- I am of the opinion that intensive strain can occur during any sporting activity, so I think that the degree of intensity should not be the deciding factor for the need for a check-up
- What does more intense mean? It's very relative
- I suggest that the term "more intensive" be defined more precisely in terms of duration, frequency, MET[5].
- Yes. It might be necessary to give examples to explain what is meant by more intensive sport and exercise programs
- There is a lack of specification here as to who this recommendation should apply to.
- Sure, but "more intensive" in relation to what? In my opinion, most likely to the person's initial training status
- Unclear ; Definition 'intensive' ??; Good offer
- more intensive is a term that can be interpreted very individually
- "more intense" needs to be defined more precisely in relation to age and gender
- Specify more precisely. Should a 20-year-old have a check-up before starting to surf?
- Too imprecise
- As quite a few examiners do not know what more intensive means, this sentence can be removed, or the term "more intensive" must be explained for the examiner who has no basic qualification in sports medicine. It is also possible that a physically active person may still reach higher, possibly risky heart rates despite the intended light or moderate activity.
- my conclusion: this recommendation can be deleted
- Is “intensive” defined in the guideline?

[5] MET=metabolic equivalents

- A definition of “intensive” has to be added

**Feasibility**

- for all persons, including young people ? certainly not feasible

**Additions**

- Absolutely. Also check after a certain time interval
- In general, preventive examinations should be carried out if sport is planned and a re-evaluation should be carried out if an increase is planned.

**Redundancies**

- Included in recommendation 1B above. Anyone who exercises, regularly means regardless of intensity and frequency!
- No additional examination necessary if one has been carried out previously
- Recommendation 1 is sufficient

**Financing**

- Pure money making ! How high is the risk of illness / death through intensive sport ? What is intensive sport

**Other**

- For injury prevention and to provide a basis for previous activity, I think this is also right
- This recommendation is important, as older athletes in particular (e.g. marathon runners aged 40 and over) can become ill when the intensity increases. Diseases can be detected!
- If you do sports on your own, the “should” is alright. it should be a must (!) if it is organized sports or in a sports club

**Suggested wording**

- insert “at least/latest”
- The term "intensive" is not unambiguous. Certainly the examination is more important for more intensive exercise than for light exercise. One could make the recommendation for everyone who does sport and add: "especially with higher intensity"
- A sports medical check-up should be carried out when starting a more intensive sport and exercise program or after a serious acute illness.
- or also when starting/restarting
- A RENEWED sports medical check-up should be carried out when starting a more intensive sport and exercise program.

**Suggested change of recommendation level**

- optional instead of conditional recommendation (n=1)
- strong instead of conditional recommendation (n=1)

**4 Alternative A: A sports medical check-up should be repeated regularly.**
**Alternative B: A sports medical check-up should be repeated regularly at intervals of 1-3 years.**

- Sports physicians: n=25 preferred A (8 of them with modifications), n=85 B (36 of them with modifications), n=14 no preference, n= 11 unclear
- Other specialists: n=11 preferred A (4 of them with modifications), n=2, n=2 no preference, n= 1 neither of the two

**Reasons against alternative A (‘A sports medical check-up should be repeated regularly.’)**

- “regularly” is to unspecific

**Reasons against alternative B ('A sports medical check-up should be repeated regularly at intervals of 1-3 years.')**

- too little scope for individuality with a fixed time interval, not feasible in recreational sports every 1 3 years.

**Reasons against both (Sports physicians)**

- characterised as an offer, only if necessary (e.g. change in health status), benefit in target population unclear

**Depending on the sports intensity**

- I think this makes sense, even the time specified. The interval seems logical. In ambitious, pure sporting circles, an interval of one year would make sense. For less ambitious hobby athletes three years would certainly be sufficient and, above all, reasonable to the patient.
- For hobby athletes every 3 years is possible, for competitive athletes every year
- Rather 1x/year with short check-ups for training control / training control every 3 to 6 months (depending on the type of sports and the available supervision)
- Not for all sporting activities, examinations can also be carried out by the family doctor or orthopaedist
- A distinction should be made here between types of sport and intensity. Amateur athletes certainly require less intensive examination intervals than high-performance athletes

**Depending on age**

- For adolescents (children and young people) annually, also adults > 60 years, all others every 3 years. Squad athletes have sport-specific recommendations and are not included here.
- every 2 years, from 65 years annually
- The older, the shorter the intervals
- Yes, age-adapted

**Depending on examination results**

- In my opinion, it depends on the findings that need to be followed-up. A flat rate of 1-3 years is based on which reasoning? Is there any evidence for this?
- I think the time interval should be determined depending on the examination results

**Depending on various aspects**

- yes, with addition at growth phase and after infectious and inflammatory diseases
- Yes, especially in the case of lifestyle changes or new illnesses; period is not necessarily or only necessary depending on age or previous illnesses
- Yes, staggered according to age and possible risk factors
- Yes, but determine time interval individually!
- From the age of 40 and at growth phase annually; at least once a year for elite athletes
- Time intervals should be determined according to the type of sport and age.
- This variant [A]. However, it is not clearly formulated without specifying a time period; it could be explained in the following paragraph. e.g. depending on the type of sport, intensity, ambition, with or without previous illnesses/previous injuries. Or simply unspecified as in variant B.
- possibly annually if there are corresponding risks, [variant B] only for people with a non-increased cardiovascular risk
- depending on the person and the intensity and risk of the sport, [variant B] may not be necessary, for high-performance sport yes, not for leisure activities in young healthy people in older people with a health risk yes every 3 years
- reasonable, regularly should be more specific, depending on age and gender

- That probably makes sense. Individually, depending on the risk profile and age, possibly after one year, but for young, healthy people after three years at the latest. This is also the case, for example, with diving fitness examinations in accordance with the GTÜM[6] specifications.
- Absolutely, every 3 years for amateur and recreational sports, annually for professional and competitive athletes. [...] In the case of persons with a previous illness a check-up may have to take place earlier, depending on the clinical picture. From a certain (biological) age, an annual check-up may also make sense
- [...] In which case after 1 year and in which case after 3 years? Specifically, it could be determined that in the case of chronic pre-existing illnesses, a repeat examination should be carried out after 1 year, for example, if physical activity remains the same. The question of "how often" should also be determined not only by specific complaints but also by the intensity of the sport. If you participate in competitions, the examination should take place more regularly (once a year).
- Adequate statement, as the time interval should depend on the state of health, age and intensity of sport (A). I do not consider a period of 1-3 years is not advisable, as the time interval should depend on the individual's state of health, age and sporting intensity. (B)

**Specify the recommendation**

- correct, but what is the interval based on? (B)
- There is no specification of the time intervals (annually? 2 annually?) (A), There is no indication of the criteria to be used to decide at what intervals the examinations should take place? (B)
- Regularly" = too imprecise (A), Better variant, but when rather 1 year, when rather 3 years? (B)
- please be more precise. under what conditions 1, 2 or 3 years ? does this depend on age, findings, state of health ? (B)
- 1-3 years is not precise enough
- How often? regularly is too imprecisely defined (A), when every year, when every 2 years, when every 3 years
- Better suggestion (A). However, I hope that the text will then explain when e.g. half-yearly inspections may also be necessary, When annual, etc. 1-3 years is so vague that it should not even be mentioned in a statement. This belongs in the text.
- yes, if no symptoms and unchanged sporting behaviour (B)

**Information**

- Fits, but should also be communicated by sports associations, e.g. soccer association
- Should also be demanded by the clubs.

**Offering character**

- not mandatory, depending on risk factors and complaints; however, the offer should be available for those who want it
- as an offer for sporting activities

**Reservations**

- f necessary! What are the criteria? (A), Why? Justification! (B)
- Only in the case of pre-existing illnesses and/or illnesses/injuries in the meantime
- Should be determined by the association and, in the case of recreational athletes, should be formulated beforehand in the event of changes in health or longer breaks.
- Too general, only in case of problems (A), Only if new symptoms occur or existing illness worsens (B)
- Only in the case of symptoms
- Only in case of abnormalities (A), Who, apart from squad athletes, needs an annual examination? (B)
- Why ?

[6] GTÜM=Deutsche Gesellschaft für Tauch- und Überdruckmedizin (German Society for Diving and Hyperbaric Medicine)

- no, I don't see any benefit in this for healthy athletes who regularly do moderate sport or health sport, before taking up or returning to sport yes,a health check is already carried out regularly every 3 years by the health insurance companies anyway (A), no, only in the case of risk constellations, otherwise see answer above (B)

**Financing**

- Individual time intervals. Emphasize the nature of the offer! In my opinion, we are facing difficult economic times. Large parts of the population will soon have no more money for things that may be useful (!), but which they used to be able to do without in previous times!
- Agree, payment by the health insurance company must be clarified so that it takes place (A), depending on the health examination, which is paid by the health insurance companies every 3 years from the age of 35 (B)

**Feasibility**

- is certainly not possible for recreational athletes in terms of time restrictions in normal practice, but the opportunity should be given
- Preferred [A], otherwise it will not be feasible to examine all recreational athletes every 1 to 3 years
- It would be nice, but hardly realistic. (A), Here too: Would be great if people would go along with this. (B)

**Adjusting the time interval**

- In my opinion, a good interval would be 2 years. Annually, possibly from a certain age
- 2-3
- Time intervals should be defined
- Yes( every 2 years)
- every 2 years
- yes, every 2 years, for competitive athletes every year
- every 3-4 years
- 2 years
- Depending on sport and intensity 1-5 yearly intervals
- 1x/year
- every 2-4 years and in case of complaints
- 5 years
- Better formulation, add on: earlier if necessary (B)
- 1 x annually.
- 1 year
- better, or at least every 2 years (B)
- Rather every 3-5 years
- every 2 years or after serious illness
- Yes, every 1 - 2 years makes sense
- every two years at the latest
- Every 3 years is sufficient
- Also self-explanatory, as restrictions can develop over time even without symptoms. At least annually.

**Other**

- Comparable to the intervals in the general medicine area. As this is a recommendation, doctors and patients still have the option of making individual arrangements (B)
- only applies to restriction for 1st recommendation (A)

**Suggested wording**

- (A) perhaps supplement or extend by. "A re-examination is recommended in the event of a new diagnosis of relevant illnesses or a re-examination is recommended in the event of a new diagnosis of

relevant illnesses or complaints during sporting activity (" red flags "All adults who (want to) engage in sporting activity, should be offered a sports medicine consultation.

- (A) Should be repeated regularly in the event of abnormalities or risk factors
- (A) Should be repeated at intervals to be determined on an individual basis, depending on the status determined. should have a...
- (B) better than A; insert depending on age/sport and intensity
- (B) should be repeated at 1-3 year intervals depending on the risk profile
- (B) good, but to be supplemented with and in case of complaints
- (B) A sports medical check-up should be carried out from the age of 40 or in the presence of a chronic illness be repeated regularly at intervals of 1-3 years.

**Suggested change of strength of recommendation**

- (B) here a should would be better placed for me
- (B) cannot must not, for high-performance sport yes, not for recreational use for young healthy people for older people with health risks yes
- 3 years

**5 Also use the results of the sports medical check-up to derive individualised exercise and training recommendations.**

- Sports physicians. n=104 positive, n= 6 positive with modification; n=7 negative, n=7 unclear, n=63 without comment
- Other specialists: n= 10 positive, n= 2 unclear, n= 6 without comments

**Suggested change of strength of recommendation**

- optional instead of conditional recommendation (n=4)
- strong instead of conditional recommendation (n=1)

**Additions**

- I suggest also including the recommendations on nutrition.

**Various**

- Notes for aerobic loads
- However, with all these formulations, it is important to bear in mind that many people/clients/patients might understand this to mean that this should be done within the statutory health insurance budget. Our system (unfortunately) does not reflect this. A recommendation will create an expectation.
- This is good, on the one hand to avoid overtraining and overloading, and on the other hand to achieve a health effect. Of course, this is only possible to a limited extent without ergometry.
- Who gives these? A trainer would be useful
- Depending on the task and objective: clarification of sport clearance and risk potential vs. performance and training recommendation.

**Feasibility**

- If there is close cooperation, then great, but this is often not the case in everyday life.
- Desirable, but probably too complex. It may only be possible to make general statements about sports in groups, e.g. endurance, strength, strength endurance, ball games, ...
- Yes,...that's the case for me, you can't really avoid it. Takes up a lot of additional counselling time though.
- Desirable, but certainly time-consuming.
- No, implementation too time-consuming - only in exceptional cases.
- Not necessarily our job.
- How so? Exercise instructors are generally not qualified enough to implement medical instructions. In order to issue training recommendations, there is a lack of time and payment.
- Which so-called sports physician actually knows anything about this?

- Only necessary in individual cases.
- What does an examiner without a basic qualification in sports medicine do with this recommendation? It must be ensured that the exercise and training recommendations are communicated on the basis of current evidence so that every examiner can provide professional advice, regardless of their expertise in sports medicine.
- No, because the implementation of in-depth training recommendations would also have to take into account individual competitive sporting aspects in specialised disciplines specialised disciplines that cannot be covered by sports medicine expertise

**6 Evidence-based communication strategies should be used to convey exercise recommendations.**

- Sports physicians. N= 46 positive, n=10 positive with modification; n=22 negative, n=36 unclear, n=73 without comment
- Other specialists: n= 6 positive, n=2 negative, n=3 unclear, n= 7 without comment

**Uncertainties**

- ??? (N=4)
- I can't imagine what's underneath.
- A meaningless phrase. What are "evidence-based communication strategies"? Sounds esoteric. Except for a small circle, nobody here knows what it means. People will only agree because it says "evidence-based". Formulate it more clearly or delete it.
- Too vague and imprecise, better to make it written.
- Generally yes, but is everyone able to understand it? What are evidence-based communication strategies?
- What does it mean?
- Unclear, personal trainer?? Who / what is evidence-based??
- So what?
- Difficult. What is meant by this at all.
- There is not even agreement on the therapeutic approach!!!
- What are evidence-based communication strategies?
- I don't understand, is it about didactics? Pedagogy? Not the primary topic of sports medicine.
- I don't understand
- ? Communication strategies?
- What is meant by this? I don't understand the recommendation.
- What exactly is meant by this?
- No: are "evidence-based communication strategies" actually available for all exercise recommendations?
- I don't understand that, what does it mean?
- What are evidence-based communication strategies in this case?

**Specify the recommendation**

- Yes, makes sense. Recommendation: Provide examples, such as ... (otherwise there may be too much room for interpretation).
- Useful - which are?
- More detailed description of the strategies.
- What exactly is meant by this? Give examples.
- would also have to be described
- More concrete formulations would be desirable. Which communication strategies? Examples if necessary.
- What are evidence-based communication strategies? Where can the examiner find them? At least, sources should be cited here. Better are explanations.
- Which ones exactly?

**Consider individuality**

- Yes, but also customised solutions!!!

- Less relevant, individualised approach to the individual.
- I see it more as adapting the communication to the recipient than individualised doctor-athlete communication.
- Too restrictive! You have to reach the athlete - however you can.
- No, I think this should be customised, but it doesn't cause any problems in daily life, especially if you are advising as a team.
- The communication of exercise recommendations should be adapted to the individual situation and take into account the positive and negative biopsychosocial factors. (Motivation, available time, workload, social involvement in sports clubs/groups, pre-existing conditions, etc.)

**Meaningfulness**

- That's nonsense!
- Superfluous.
- Not very meaningful.
- Too theoretical.

**Feasibility**

- Who is trained in communication and what are evidence-based communication strategies in everyday medical practice?
- .....The main thing is that people get moving and are informed about the risks in relation to their state of health, training condition - which communication strategy is used doesn't really matter...may make it too complicated in practice (scarce time resources).
- Correct, but sometimes difficult to implement.
- Great effort, uncertain advantage!
- I find the wording difficult! What exactly is meant? Motivational interviewing? Yes, but it's not that easy.... How many opportunities do doctors have (in terms of time and the number of programmes) to learn evidence-based communication strategies? I am very interested in this, but I see considerable difficulties in practical implementation. Solution: The DGSP trains us all regularly via online programmes.
- How should this be realised?
- Too complicated!
- How and where are they conveyed? I've been to a lot of sports medical advanced training, but unfortunately this was very rarely a seminar topic.
- The main thing is to look at what can be realised in daily life and, of course, to help the athlete on the way to individual movement planning.

**Relevance**

- Too specific, actually applies to all areas of medicine.
- This is true, but should actually be self-evident and applies to all areas of medicine. For me, this is superfluous.
- There is a danger that more important points will lose importance if such aspects are mentioned "equally".
- Moreover, strictly speaking, you would also have to mention how exactly an examination is carried out, for example.

**Additions**

- And above all evidence-based training concepts.

**Other**

- Please use plain language that is easy for patients to understand.
- If, then like this.
- Not necessarily only.
- I tend not to use it, I advise and recommend based on my experience.

- Yes, e.g. EFSMA guidelines.
- Training recommendations standardised as safety barriers
- Sounds more like a "speech bubble".
- Sounds far too pompous for recreational sports.

**Suggested wording**

1. I think it's good, possibly suitable/evidence-based".
2. Among other things yes.
3. If possible.
4. Or better individual communication strategies, athletes are rarely evidence-based ...
5. Yes, if possible.
6. I think the word "evidence-based" could be replaced with "individualised", because the recommendation should always be individualised to best communicate with the athlete.

**Strength of recommendation**

- Can, depending on the type of sport.

**7 Alternative A: A sports medical check-up should be carried out by a specialised doctor, preferably with certification in sports medicine.**

**7 Alternative B: A sports medical check-up should be carried out by a specialised doctor with certification in sports medicine.**

- Sports physicians. n= 39 favoured A , n=86 B (2 of them with modification), n=12 no preference, n=1 none of these, n=8 unclear, 41 without comment
- Other specialities. n=8 preferred A, n= 4 preferred B, n= 2 unclear, n= 4 without comment
- Reasons for A: Other qualification sufficient, ensure comprehensive care, ability to delegate
- Reasons for B: Provide qualification through additional qualification, strengthen additional qualification

**Arguments for alternative A ('A sports medical check-up should be carried out by a specialised doctor, preferably with certification in sports medicine.')**

**Alternative qualification**

- Yes, as long as it refers to the restricted population mentioned under 1.
- In professional sports/elite sports, yes, but not necessarily required in amateur sports.
- There are certainly capable doctors without the additional qualification, therefore A.
- It would be desirable, of course, but a doctor without the additional qualification but with appropriate cardiologic and orthopaedic expertise could also perform this; hence formulation A.
- Generally okay, I am only speaking for myself here (without the additional qualification, due to childcare and various other circumstances initially no time, then later didn't see any point in it): I repeatedly receive "preliminary findings" brought by doctors with the additional qualification, where for example the exercise ECG (if done at all) only goes up to 200 watts (for athletes who reach over 300 watts)... or various sports physicians are miles away from the topic of sports themselves, so the additional qualification does not seem to be THE quality criterion to me; alternatively, one could perhaps also provide documented evidence of, for example, 50 examinations... in that respect, I would find the "preferably" quite good.
- No, there are also excellent doctors without it, and the distribution of sports physicians is not sufficient.
- From my point of view, the additional qualification in Sports Medicine is not absolutely necessary if the respective doctor has the necessary experience.
- Correct, the sports physician should be aimed for, as they hopefully have the corresponding knowledge, but other doctors may also be equally competent. To ensure that as many athletes as possible undergo the examination, they should not be excluded. If abnormalities or uncertainties arise, one should assume that a corresponding referral will be made.
- Ideally yes, possibly with a doctor with the appropriate expertise.

- There are also doctors with extensive experience in sports medicine without the additional qualification.

**Comprehensive healthcare coverage**

- Yes. Also allows enough room for medical trainees or colleagues who deal with this (e.g., also in the context of club support, as company physicians, etc.), but (still) do not possess the additional qualification; I do not consider strict restriction to the additional qualification necessary, but believe that medical trainees with appropriate guidance, club physicians who have been dealing with these aspects for a long time, and company physicians can conduct these preventive examinations; therefore, in my opinion, the term "preferably" is more appropriate.
- That would be my preference, as there are many colleagues who have good sports medical knowledge, even without having obtained the additional qualification in Sports Medicine. Furthermore, there are rural areas where it is difficult to reach a colleague with the additional qualification in Sports Medicine, so that athletes may forego the examination in the worst case scenario because they cannot find a sports physician.
- To be able to offer corresponding preventive examinations nationwide is preferred.
- Yes, a larger circle.
- Would be okay, because more widely available.
- Probably not enough sports physicians nationwide.
- Do we have enough sports physicians?
- I think here we should approach general practitioners (GP) and paediatricians.

**Other**

- Not so much exclusivity! (regarding B)
- Better than insisting on the additional qualification. The additional qualification is rather rare and could (at least in the early stages of the guideline) lead to resentment.
- It should be delegable to a medical trainee; please translate into English.

**Arguments for alternative B (‘A sports medical check-up should be carried out by a specialised doctor with certification in sports medicine.’)**

**Qualification**

- I consider this to be absolutely essential. Preventive sports medical examinations should only be conducted by doctors with the additional qualification in Sports Medicine. Firstly, from a professional perspective.
- Better suggestion. I hope this will be confidently formulated in the following text. No one will dispute that internal medicine checks are carried out by internists, paediatric checks by paediatricians, orthopaedic checks by orthopaedists, etc. And with this understanding, sports medical examinations should also be carried out by the respective specialists with the additional qualification in Sports Medicine. It is a matter of course and should also be clearly communicated by us.
- Exactly. Only they can assess a situation or load properly.
- [A] Rejected, as not every doctor possesses the necessary knowledge.
- This variant is better, as a higher quality can be expected from sports physicians!
- Exclusively by sports physicians, firstly these colleagues have a broad interdisciplinary perspective through their training and are qualified for it.
- Always. A prerequisite so that individual training recommendations can be made. It is not sufficient here to just indicate the stress zones.
- Variant B, because athletes can only be adequately served with sufficient expertise.
- Sports medicine should be obligatory. Where else is the distinction from a typical "check-up"? Assessing findings in relation to physical activity is the added value of a "sports medical check-up". For this qualitative added value, only expert assessment is appropriate and does not allow for reliable standards, moreover, it is not objectively recognizable for patients.

- Better variant in terms of quality assurance. Anyone could be knowledgeable, in principle, according to their own assessment. Only the additional qualification ensures a basic level for the preventive examination through training and examination.
- Would be better because better professional competence can be expected

**Strengthening the additional designation**

- Secondly, there is otherwise no longer any incentive for doctors to obtain the additional qualification of sports medicine. As a long-standing organiser and scientific director of sports medicine training events, I know what I'm talking about
- Favourite alternative. This strengthens the field of sports medicine.

**Other**

- It is "our" guideline, therefore with the additional designation of sports medicine.
- Exclusively. Everything else makes us almost superfluous as sports physicians
- Yes, absolutely. I don't think that doctors who don't have the additional designation can't carry out preventive examinations. However, having the additional designation demonstrates an interest in the topic, which is essential for sound advice. well-founded counselling
- Yes, in order to establish a valid standard. However, the speciality and any additional designation should be taken into account. An internist and a surgeon have different specialisms and different skills. Ideally, there should be a permanent team (distributed practices and clinics or in an MVZ) that knows each other. Difficult to realise. Alternatively, every 5 years sports physicians could
- could organise an examination course as a revision course (similar to the X-ray refresher course or emergency medicine refresher course)
- Even though I'm not a sports physician, I would favour this alternative - the qualification makes sense and the guideline should support it. also support this.

**Further discussion points**

**Limitations**

- ok, but here too the restriction specialist therefore musculoskeletal system only orthopaedist. Lung/heart/circulation internist/cardiologist/GP.
- Then it has to be determined who can do this? Is there mandatory further training?
- Yes, but not all doctors with this additional qualification are sufficiently qualified.
- If you consult alone, yes. Ideally, the examinations should take place in a centre that specialises in this. The additional qualification in sports medicine is "just decoration" for many doctors in private practice

**Arguments against both variants.**

- regardless of whether it is "preferable" or not, each person alone decides who carries out the sports medical check-up. this is possible for any doctor with the appropriate equipment. the guideline should include all groups here: a) you are a doctor without the additional designation and your patients want you to carry out a sports medicine check-up. b) you are a doctor with the additional designation of sports medicine. b) you are a doctor with the additional designation of sports medicine. there are two different sets of information for a and b in order to ensure the quality of the examination.
- No to exclusive waste of resources
- Why not go to your GP?

**Other**

- Yes, I think that's right and important, I often see aptitudes etc. in everyday life where only one auscultation was performed, nothing else, especially in the case of recreational athletes who train intensively should also have an ECG etc. (due to my cardiologic background, I also carry out a very low-threshold ECG) - I think this is extremely important, better wording (A)
- yes! it must be made clear to patients/clients that there is a specialised internal medicine (internal medicine specialist...) AND a specialised orthopaedic (ophthalmology specialist) examination.

- and family doctor, general practitioner, occupational medicine, in case of chron. disease, he should also be seen regularly by a specialist (A), but not necessarily (B).
- and, above all, the doctor should be known for exemplifying his "recommendations" himself. This is a fundamental pedagogy. No "overweight" or / and "smoker".
- Not provable with a very heterogeneous collective of sports physicians, but good propaganda. (B)

**Suggested wording**

1. See above but better - why should??? "A sports medical check-up is carried out by a specialist doctor with the additional qualification a specialised doctor with an additional qualification in sports medicine."
2. A sports medical check-up should always be carried out by a specialist doctor with the additional qualification of sports medicine.

**Strength of recommendation**

- Why a conditional instead of a strong recommendation? Variant B would be better

**8 A standardised medical history form should be used as part of the sports medical check-up. This should include the following items:**
**• Personal and family medical history**
**• Individual risk profile**
**•Medication anamnesis** *[added subsequently]*
**• Participation in earlier check-ups and their results**
**• Information on surgical procedures and injuries**
**• Nutritional history**
**• Gynaecological history**
**• Vaccination status**

---

- Sports physicians: N= 70 positive, n= 48 positive with modification, n=3 negative, n=8 unclear, n=58 without comment
- Other specialists: n= 7 positive, n= 5 positive with modification, n= 6 without comment

**Medication and supplements**

- And above all evidence-based training concepts
- Supplements/medications.
- Yes Medication history
- Taking medication (including the pill) and nutritional supplements and painkillers!
- Intake of medication and dietary supplements
- Medication history, especially use of painkillers
- Medication history should be supplemented
- The section "Medication" could be included under "Diet" or extra
- Medication plan
- Medication status, especially lifestyle products
- Medication history
- The anamnesis on medication, dietary supplements and other regular intake of pharmacological products seems to me to be not insignificant (I always collect it)

**Family anamnesis**

- The family anamnesis should be specified very precisely
- Unconditional and obligatory: Sudden cardiac death in the immediate family up to the age of 50 years

**Sports anamnesis (see recommendation 10)**

- Training profile is also part of this
- also sports anamnesis!
- Good. + Sports anamnesis see 10.

- Sports anamnesis/exertion
- Please add: Sports anamnesis
- In addition, an anamnesis of previous sporting activities, if applicable, period of interruption, "return to sports",
- Clear In addition, previous sporting experience and current status
- Type, scope and intensity of sporting activities
- Survey of the type of sport and the intensity or goals (of course see recommendation 10)
- Current and previous sporting activities must be urgently enquired about here
- Sports history, training intensity, performance level, participation in competitions
- Problems during training
- Training goals Planned training effort/competition goals Previous experience in sport
- In addition, the individual sports history should be asked about (see recommendation 10)
- Sports history, how much experience? If necessary, derive training recommendations for useful intensity/extent of sporting activity
- Sports history and preferences/interests
- Training volume and types of sport
- In my opinion, the sports anamnesis should be supplemented, what plans or experiences exist, how do you currently train... (see below).
- + previous sports experience
- Physical activity anamnesis! Physical activity as a vital sign!
- Enquiry about sporting goals!
- Sports experience in the past
- Training intensity
- especially sports anamnesis
- Sporting activities (both no longer practised and active sports)

**Diseases and disabilities**

- Infectious diseases?
- I suggest explicitly specifying the risk profile with regard to psychological / psychosocial stress. This also corresponds to the recommendations of the IOC.
- Pre-existing orthopaedic conditions
- The question about hospitalisation often helps to jog the memory and brings up interesting problems that were often not mentioned in the above questions
- important: disabilities and functional limitations
- Corona infections?

**Other**

- Laboratory values from the last 1-3 years should be included
- Possible complaints should also be recorded in a standardised form
- Goals of the patient
- Supplement: - Current complaints? Which ones?
- Current complaints
- Last dental/ophthalmological check-up
- Allergy/intolerances
- Prevention issues

**Various combinations**

- Previous illnesses and medication should definitely be recorded
- Everything ok, supplementary: immunisation status optional, nutritional history certainly useful, but realisable (in detail), possibly like this: orientating Nutritional history, recommend nutritional counselling (possibly with reference to sports nutrition) in the event of abnormalities. Previous training anamnesis / competition experience (see below)
- Additional questions from the "smaller" disciplines such as dermatology, ENT, dentistry/ orthodontics, eyes,...

- Gynaecological & urological? // medication and nutritional supplements // allergies // optional: aids (glasses/contacts/orthotics etc.)

**Modifications**

- I am against recommending medical history forms. These questions should certainly be asked, but this can be done just as well or better in the as part of the medical history. I'm not in favour of introducing a mandatory questionnaire. We have tried this and it makes more work for us more work than it saves us time. We also didn't notice any qualitative advantage and therefore cancelled the questionnaires again. stamped out again. Anyone can do what they want. The decisive factor is the content and not the form of the survey. A recommendation should not be made.
- Nutritional history not so important and immunisation status totally unimportant, add drug history
- Results of previous screening - you can do without it! It's only useful in theory, but it's a lot of work! And in practice that many things are desirable. But money has to be earned during working hours, effectively. Unfortunately, but that's the way it is!
- I think the current DGSP questionnaire is quite good. Ad gynaecological. Anamnesis: in relation to RED-S? This could also affect men and would then not be gynaecological. Special questions with regard to unrecognised cardiovascular diseases are important; medication must be asked, sports history is also relevant for the interpretation of the results. Immunisation status: I always discuss this, but it is perhaps more the task of GPs.
- Nutritional history, can't do anything with it, too extensive in practice, consultation time...
- Nutritional history is difficult to standardise, I would leave it out. General information with the help of a flyer is sufficient in my opinion.
- useful; why vaccination status: not relevant

**Limitations**

- Yes, if you don't know the patient.
- gynaecological history only optional for women

**Uncertainties**

- Immunisation status??

**Criticism**

- Not too extensive
- In my opinion, it can be formulated more briefly. Such a detailed list runs the risk of forgetting individual things (e.g. medication/supplement anamnesis)
- Could certainly be simpler.
- Much too complicated, a simple basic set would be good and, if necessary/desired, expanded to include the points mentioned.

**Other.**

- Standards advantageous
- yes; if necessary before gynaecology. A

**Strength of recommendation**

- Still too much "should" - either it belongs in the recommendation or not...
- Vaccination status only if travelling abroad or other risk.

**9 A validated score should be used to assess the cardiovascular risk.**

- -Sports physicians: N= 73 positive, n= 21 positive with modification, n=8 negative, n=12 unclear, n=73 without comment
- Other specialists: n= 6 positive, n= 3 positive with modification, n= 2 unclear, n= 7 without comment

**Limitations**

- sounds useful, but should only be understood as a decision-making aid
- If applicable
- My favourite. If it is a score of 2, the necessary laboratory values should also be recorded
- Depends on the score!
- Yes, if available for the sport
- Yes, but the score should be standardised and reflect hard criteria
- However, it should be designed in such a way that little effort is required and may lead to a referral to cardiology, for example. The score must not be so comprehensive that only cardiology can collect it. Otherwise it would hardly be collected.
- It can be done, but it may be very high for a completely healthy person.
- I don't know what is included in the score. Otherwise, in my opinion, asking about the risk factors in the medical history would be sufficient.
- I am familiar with scores, but physical activity is usually missing. is there a score that includes physical fitness? more important than established risk factors in terms of overall mortality.
- my conclusion: either name a valid score or the recommendation can go.

**Specify**

- Yes, but the score should be standardised and reflect hard criteria
- Which of the scores?
- Suggestion: Arriba.
- More specific information or examples of validated scores and which are suitable for fulfilling the guideline. Formulation: A validated score is used.
- Yes, SCORE of the ESC
- yes. but specifically which one ???
- Yes (SCORE2 or SCORE2-OP).
- Yes, ideally at least one score should be proposed here
- A list of suitable scores should be provided here.
- SCORE 2 according to Prev. LL ESC
- Suggestions for such scores would be helpful...
- Gladly with an example
- This should be specified
- yes, but which score is the best? JBS3? Procam?
- Which one?
- E.g. SCORE 2 of the ESC
- Weighing up and recommending the existing scores would be desirable

**Criticism**

- Health assessment more important than score, exercise ECG, blood pressure setting, etc.
- No, I think that's too time-consuming. We should always ask ourselves where our focus is? I mainly see recreational athletes and ambitious amateur athletes. Patients with an increased cardiovascular risk (cvR) generally do (too) little sport and need to be informed by their GP or cardiologist about their overall cvR. I always take 2 hours, but the price for the sports medical check-up should not be that high. If cardiovascular risk factors are present, these are of course discussed. I don't need a score for that.
- No, there are only inadequate ones for intensive exercisers
- No! I prefer an assessment based on risk factors or previous illnesses
- Only possible to a limited extent, as all scores do not include physical activity
- I consider a simple ECG to check heart function to be sufficient for a BASIC EXAMINATION; further diagnostic measures are the responsibility of the statutory health insurance provider and belong in the hands of specialists
- Can be omitted, as this says nothing about the CURRENT fitness for sport
- Rather no. Which one? Validity varies.
- Scores are for specialists ..... As a practitioner, I need a simple basic check

**Age-dependent**

- possibly age-dependent? Is the score so clearly superior to the basic consideration of cv risk?
- from a certain age e.g. 35 years/45 years

**Other**

- good--here's a note: I'm currently observing an extreme increase in athletes who have atrial fibrillation. Most likely due to Basis infection (Corona?)...without "conventional cardiovascular risks".
- Emphasise recommendation!
- That goes without saying. However, sports should still be done in almost every cardiovascular risk constellation

**Suggested wording**

1. among other things
2. absolutely necessary? This makes little sense for young people. But certainly for older people. Perhaps rephrase it as follows: "For people over ... years of age and with existing cardiovascular risk factors, a validated score should be used."

**Strength of recommendation**

- I would tone it down, because not everyone needs a score. It can't be used equally well for everyone either, the information doesn't come across equally well for everyone using a score. Suggestion: "The use of a validated score can be helpful in assessing cardiovascular risk"
- Scores should be used for assessment
- strong instead of conditional recommendation please and yes important point
- can

**10 A sports history should include questions about**
**• the frequency, intensity, duration and types of sport**
**• previous experience with sport**
**• Goals of the sporting activity** *[added subsequently]*
**• Environmental conditions during sport such as heat, cold or altitude**
**• complaints at rest and during exercise**
**• individual sport-related risk profile and**
**• any aids or restrictions (e.g. visual aids).**

---

- Sports physicians: n=113 positive, n=4 negative, n=2 unclear, n=68 without comment
- Other specialists: n=9 positive, n=0 negative, n=9 without comment

**Effort**

- You can do it this way, but it is very time-consuming

**Criticism**

- Too extensive for the recreational athlete
- KISS keep it simply stupid - Do you have any complaints? What sport do you want to do? What previous sporting experience do you have? This should lead to a conversation.... Otherwise nobody does this in practice!
- The doctor should take a sport-specific medical history. Just don't make it too complicated with endless questionnaires
- It can be even more complicated. Unsuitable in practice.

**Additions: goals**

- The goal is also essential. Does he want to go jogging for health reasons or improve his marathon time to under 3 hours?
- plus the patient's / athlete's goal (getting back into running, ..., competitions, ...);

- What is the aim for the future?
- Goals of the sporting activity? Intensification planned? possibly participation in a marathon or similar?

**Further additions**

- previous sporting experience should be specified in more detail, performance tests should be asked about, greatest successes (if?) should be queried
- the sports equipment used
- Information on the support team (coach/athletics, etc.) or level of knowledge in this regard is also welcome
- Training intensity, performance level, participation in competitions, squad status, problems during training
- Structured training? alone or in a group, what pulse rates, what successes?
- Previous performance diagnostics results
- Preferences for various sports/types of exercise
- Also important: sports shoes and sportswear!

**Provide a questionnaire.**

- Such a questionnaire would also be extremely helpful

**Limitations**

- Environmental conditions during sport, such as heat, cold or altitude, are only relevant in special cases or in competitive sport
- Some things just happen and don't need to be asked. It's hot at the Ironman in Hawaii, high and cold on Mont Blanc
- Ambient conditions for whom? At what times? Rather a topic for sports counselling
- Environmental conditions do not necessarily have to be asked, the possible aids also play a subordinate role
- Only for certain questions

**Individualisation**

- Individualised objectives here too
- Depending on the type of sport, different priorities must be set, e.g. eyes and nervous system (head injuries) in contact/fighting sports or ENT and lungs in diving (although the examination should be somewhat more specific here anyway).

**11 Obtain a whole-body assessment as part of the sports medical check-up:**
**• Collection of anthropometric data (height, weight, abdominal girth, calculation of body mass index)**
**• Pulse and blood pressure measurement at rest**
**• Examination of heart and lungs**
**• Assessment of vascular status**
**• Abdominal examination**
**• Examination of the musculoskeletal system including a) mobility and stability of the large joints b) mobility and tapping pain of the spine c) muscle status / function, tone, etc. Examination of the nervous system (gait pattern, reflex status, sensitivity, cranial nerve status if applicable)**
**• Examination of the sensory organs (incl. visual acuity assessment with eye charts)**
**• Inspection of the skin**
**• Inspection of the oral cavity, recording of dental status**
**• Assessment of lymph node status**

---

- Sports medicine n= 57 positive, n=37 positive with modification, n= 18 negative, n=18 unclear, n=64 without comment
- Other specialists: n=6 positive, n=3 positive with modification, n=0 negative, n=2 unclear, n=9 without comment

**Feasibility and extent**

- Quite time-consuming for the daily GP practice -
- Ideal situation. In my experience, this is not feasible in terms of time, patient understanding or financially anyway.
- Each point can be useful under certain circumstances. But - "reduce to the max!". A child wants to take part in canoeing competitions. Does he really need a "vascular status"? Surely some combination is theoretically conceivable where the status may be needed. But the whole thing has to remain manageable and not cover every possibility in principle. A sports physician can choose (!!) what he needs. Perhaps a template where you can tick a box? But not everything has to be ticked?
- Desirable but not feasible in practice ...
- In principle, yes. But probably not feasible in everyday life? From examination of the nervous system downwards.
- ok, very extensive
- Too extensive
- Cf. recommendation 9, effort should be realisable in daily practice and also affordable, in the sense of "realisable with home remedies".
- can only be carried out to a limited extent in outpatient medicine due to the prevailing time pressure and lack of staff; in any case, a brief whole-body status should be taken.
- Everything must be comprehensive, especially lk's, dental status, visual acuity. Only adapted to the respective sport. Marathon has other problem areas than diving
- There is certainly a lot to be trimmed from the list. This scheme cannot be used for all age groups as it is far too extensive and time-consuming. Not realisable in practice
- In an internal sports medicine examination with the aim of avoiding cases of sudden cardiac death in sport, a detailed examination of the nervous system / sensory organs / oral cavity / dental status is not practicable, cannot be integrated into the time frame and is not covered by GOÄ8 (€34.86).
- A lot of effort
- Neurological examination too extensive in my opinion, not feasible in terms of time in a statutory health insurance practice,
- also lymph node status, skin dispensable in my opinion
- Very accurate and extensive so
- Too detailed for purely recreational athletes
- This scope will NOT increase acceptance. It is not always appropriate.
- For squad athletes. Can't it be simpler?
- Completely exaggerated in recreational sport

**Additions**

- Possibly vision and hearing
- Yes Coordination tests
- Yes, if necessary, specialist co-assessment by dentist, ophthalmologist, neurologist
- ECG ergometry. Lung function if necessary
- Additional psychiatric illnesses (e.g. depression, anxiety disorders)!
- In any case, a resting and exercise ECG should be performed; ideally also a cardiac echo; the lung examination should include at least a "small spirometry"
- Full body status is a matter of course. I would add coordination to the nervous system; I only consider an eye test to be only optionally required for specific sports.
- ECG and stress ECG for endurance athletes Balance test
- The orthopaedic examination could be more detailed with more room for additions
- Length differences of the extremities!

**Individualise (depending on sport/intensity)**

- I think this is too extensive! The examination should be focussed on the type of sport (e.g. visual acuity only for shooters, for example). Does the whole-body status really evidence-based for risk minimisation/detection of dangers for sport?
- The scope of the examination should be focussed on the sport being played.

- I would make this dependent on the chosen sport. A vision or hearing test is certainly not always necessary
- Dependent on popular sport, intensity of exercise
- BMI interesting for an overview, but not really state of art anymore...? the whole body status could be assessed by the various disciplines (specialist level) depending on ambition (amateurs, squad, paid sport) - this should also be budgeted separately??? this goes beyond any normal consultation and thus becomes unrealistic - which is sad
- should"? So always? That would be too restrictive! Status assessments should be carried out depending on the type of sport and the individual risk profile
- For competitive athletes, yes, for recreational athletes, depending on the extent and type of sport.
- Modified for the respective sport
- shouldn't we sports physicians limit ourselves to examinations that are directly related to physical activity? for people who are free of complaints, who want to take part in intensive sport, not all of the above examinations are necessary to determine their fitness.
- Depending on the impression, individual points mentioned above can be queried
- I think that the examination of the nervous system only needs to be carried out if there are specific questions or complaints
- I think it makes sense to provide differentiated information if specifications are made. For example, how much or how should a vascular status be assessed in an 18-year-old? In my opinion, an orientating vascular status is completely sufficient here, provided there are no vascular diseases in the medical history. When examining the heart and lungs, you can simply say that this was done by listening to the heart and lungs, but when it comes to the vascular status, I would find it difficult to palpate only the relevant arteries with the wording "assessment of the vascular status" (without restrictions). I would be more comfortable with the wording "orientating". This also applies to the abdominal examination, where palpation of possible hernial orifices and the size of the liver/spleen would suffice as an "orientating examination" in a young athlete. The same applies to the musculoskeletal system, where I won't determine the range of motion for every joint in a 20-year-old if this is suitable for orientation purposes, and even less so the muscle status. I consider the examination of the nervous system and sensory organs (as explained) to be useful (although this is actually only for orientation purposes). However, when it comes to recording the dental status (incl. tooth decay?), I wonder again how extensive this needs to be for a 20-year-old recreational athlete. That's not even recorded for fitness for sea service. As for the lymph node status, an orientating examination would be enough for me ...

**Anthropometric data**

- Don't worry, I know the literature. I don't think it makes sense to measure the abdominal circumference when I have height and weight and see the athlete in front of me. Also, the standard deviations in the abdominal circumference measurement are too large. It costs time and has no added value in this setting.
- BMI is of course somewhat questionable for athletes....especially in weight training....
- Cancelling abdominal circumference makes no sense
- Point 2: Waist-to-hip ratio is better than BMI; heart rate measurement in addition to pulse? Last dental examination/treatment, vascular status where and how?
- BMI is not explicitly required if you have height and weight.
- instead of BMI - Waist-to-hip ratio
- BMI alone is not enough and should be viewed critically. Otherwise unreserved agreement.
- BMI does not fit well with very muscular athletes. Perhaps a body fat measurement would be a better supplement here

**Dental status**

- Dental status: here I always point out the importance of semi-annual to annual examinations and ask about the last visit to the dentist.....because I think it's a bit "presumptuous" to ask for a status myself.
- Too extensive for a normal check-up. A non-dentist cannot assess the dental status anyway.
- Inspection of the oral cavity and dental status only in the event of symptoms in this area or for certain sports, e.g. diving, flying.
- Oral cavity and tooth debatable

- Orienting assessment of hearing is missing Oral cavity ok, assessment of dental status goes beyond a preventive examination and should be carried out by a dentist if there are any abnormalities.
- No dental status.
- What does dental status have to do with sport?
- Assessment of dental status should be left to the dentist

**Lymph nodes**

- What does the lymph node status have to do with sport? The sports medical examination is not a general preventive examination for chronic diseases.
- Lymph node status is too much. Only if there is a personal or family history. The same applies to visual acuity. Only for sports where this is important (shooters, golfers, etc.).
- Why lymph node status?
- Lymph nodes also debatable. Overall, too extensive a plan.

**Visual acuity estimation**

- Yes However, I consider visual acuity assessment with eye charts to be exaggerated and also not feasible in terms of time (for cadre athletes: yes).
- Visual acuity assessment is often somewhat difficult in practice, as rooms often do not offer the appropriate distances and lighting conditions and is also rather time-consuming but certainly useful for certain sports (optional if necessary)
- Lymph node status is too much. Only if you have a personal or family history. The same applies to visual acuity. Only for sports where this is important (shooters, golfers, etc.).

**Various**

- Lymph node status, visual acuity, cranial nerve status, reflexes are not necessary in the screening examination, if these are pathological, other specialists are required
- Sensory organs without eye charts, skin inspection of the skin, oral cavity, dental status depending on sport and then have it done by a specialist if necessary for sport ... very extensive, everything must also be documented.
- Examination of functional disorders and limitations
- only if required
- For example, examination of the sinuses for tapping and pressure pain as well as an otoscopy for swimmers and divers to rule out otitis, eardrum injuries, scars or tubal catarrh. What is also missing is an examination for hernias.
- In the case of the spine, "knocking pain" can be omitted as it is not evidence-based and "pressure pain" would then also have to be included.
- Neurologist/reflex status only in the case of pre-existing conditions.
- Skin only within the scope of the remaining examination. Lumbar node status groin and clavicular.
- Remove muscle tone if necessary as it is difficult to objectify

**Finances**

- However, very good examination proposals. should also be rewarded accordingly
- Yes, but should also be paid
- Agree Payment must be clarified for practical implementation
- Cf. recommendation 9, effort should be realisable in daily practice and also affordable, in the sense of "realisable with home remedies".
- this should also be budgeted for separately??? this exceeds every normal consultation hour and is therefore unrealistic - which is sad.
- It cannot be integrated into the time schedule and is not reflected in GOÄ8[7] (€34.86).

---

[7] GOÄ=Gebührenordnung für Ärzte, fee regulations for doctors

**Limitations**

- Yes, this can usually only be done on an interdisciplinary basis
- This seems redundant and, in my view, does not need to be mentioned again!

**Suggested wording**

1. A whole-body status is recorded...
2. not explicitly whole-body status, but general internal and joint status
1. Whether this is really necessary for every examination to this extent seems excessive to me, e.g. for adolescents and younger patients / athletes. Possibly better: "... A comprehensive physical examination considering internal medicine and orthopaedic aspects, which, as appropriate, should include the following points among others: ..." The BMI is a contentious parameter; reference to an analysis of body composition would be preferable.

**Strength of recommendation**

- change strong to conditional recommendation
- In fact, this should be part of a fitness test etc. before taking up more intensive sport! I do it, after years it goes quite quickly and I get a good overall picture - please again change strong to conditional recommendation

**12 Consider measuring muscle strength as part of the sports medical check-up.**

*Remark subsequently added:* **Muscle strength can be determined to measure muscular fitness, e.g. by measuring hand strength**

- Sports physicians: n=48 positive, n=28 positive with comment, n=34 negative, n=3 unclear, n=74 without comment
- Other specialists: n=6 positive, n=3 positive with comment, n=2 negative, n=0 unclear, n=7 without comment

**Criticism**

- not as part of preventive care, muscle symmetry and circumference during physical examination are sufficient in my opinion. Measurement of strength only as part of a performance test with specific training counselling
- I do not consider this necessary for all patients; rather unnecessary; not relevant; unimportant
- what for?; purpose??? For me, this falls more into the area of training management, physiotherapy or similar and not into the medical area. What effort should be made? The sense and benefit is not clear. There are various options here, the results of which are hardly relevant to recommendations
- has little medical significance; And then? Off to the dumbbells? If the athlete enjoys it, then he's already doing it anyway. And if he prefers air rifle then he can put muscle strength in 2nd place...
- We should leave strength measurement to sports scientists or physiotherapists
- I do not consider it important and dispensable. It is very important to me that a basic sports medical examination must be carried out at a certain medical level, nevertheless, it should be possible for any sports physician to carry out all the examinations required. If too much detailed examination are required, a sports physician can no longer perform all the basic examinations, the patient has to go to different doctors for one examination, which no one will go along with and will also annoy the authorised sports medicine examination centres
- A measurement of muscle strength will only rarely be available. A measurement according to Janda remains an individual assessment. I would not assume that, as it also has no influence on the recommendation and the counselling in relation to the extent that the athlete has indicated or can perform

**Feasibility**

- Absolutely not practicable - but only a can-recommendation; also rather not realisable in practice; however, only very few will have the opportunity; time-wise not possible in a statutory health insurance practice
- this will probably be necessary and feasible in very few cases
- I see the examiner's assessment as leading here, a general muscle strength measurement would be quite complex or would require time and equipment.
- can go beyond the scope of a general sports medicine examination!
- is not possible without special equipment; not using machines, only possible manually in the practice; only possible in a simple setup (manual strength measurement)
- this will be difficult to standardise; very subjective....objective measurements are not possible in practice; and how to measure? Difficult to implement in practice; standardised power meters not available for all muscle groups, measurement by degree of strength too dependent on the examiner

**Indication**

- For which indications? These should be added in a list, such as for ...
- very useful as a baseline determination
- is more likely to be associated with geriatrics (frailty); possibly only for older people
- but rather in the context of muscular imbalances
- targeted with regard to sport and patient; for specific questions, type of sport etc.; in the context of specific questions; for specific questions; depending on the type of sport ; if necessary for special sports
- if interested or required; if desired; only specialised on request...; only if necessary
- rather indicated after specific injuries such as cruciate ligament rupture in the context of return to sport, therefore not necessarily worth mentioning for me in this context

**Method**

- But then please use specific tests to be able to compare;
- possibly hand force measurement; hand grip is sufficient in my opinion; hand force both sides;
- Realisation? Which muscles? Repetition or maximum?; Which muscles? How? Statement? Simple sports motor tests please;
- but extended here: which strength tests? Which ones/goals? Either it is a specific and more in-depth examination or it does not need to be
- listed separately as it is (not should be) included in the whole body status;
- Specify how and which muscle strength should be measured; but how and why?

**Execution**

- according to Janda or objectively technical? then standardisation...;
- at least force level according to Janda, otherwise of course also goniometer or other equipment
- only best clinically

**Suggested wording**

1. A measurement of muscle strength may be recommended or carried out as part of the sports medical check-up be carried out.
2. An orientation measurement of muscle strength can be carried out as part of the sports medical check-up.
3. ... an orientating manual or, if necessary, instrumental measurement of muscle strength should be carried out
4. measurement of muscle strength using a hand ergometer

**13 A full blood count, pre-prandial blood glucose concentration and lipid status, liver/kidney values, electrolyte concentration and urine status can also be determined if necessary.**

- Sports physicians: n=67 positive, n=22 positive with comment, n=22 negative, n=11 unclear, n=65 without comment
- Other specialists: n=5 positive, n=2 positive with comment, n=3 negative, n=1 unclear, n=7 without comment

**Criticism: by default?**

- Blood glucose and lipid profile (LDL and TG) should become standard in a sports medicine examination; lipid and blood glucose values should be binding in adults over 35 years of age (ESC LL Sports Cardiology).
- I consider blood glucose, lipid status, kidney values and small BB as well as urine status to be essential
- A laboratory status is rather mandatory; normal blood test as standard; I would write this as a clear recommendation for everyone - not as a supplementary; as a matter of course as part of a preventive internal medicine programme; is obligatory; mandatory; not just "if required", but be part of the sports medicine examination as standard; not "if required" but "always as part of an initial sports medicine examination or in the course of it".
- cannot but should - if they are not from the last 1-3 years
- Should be part of the examination, lipid profile optional with corresponding risk constellation, kidney values obligatory, TSH useful
- Urine status always

**Better with the GP?**

- At the GP. Current blood count by GP must be presented for examination
- If these values have not already been determined elsewhere (GP check-up)
- Belongs in the check-up; the GP is responsible, the athlete should go to the check-up; Referral to GP in case of clinical indications
- However, I am hardly interested in such values in the sports area, but rather in the prevention area
- The laboratory test should also be carried out by the GP prior to a planned sports examination. However, the legislator should also have the costs covered by the health insurance funds. This should be a valuable component of the planned prevention strategy of the federal government.
- We sports physicians should not limit ourselves to examinations that are directly related to physical activity? in the case of people without complaints who want to do intensive sport, not all of the above-mentioned examinations are necessary in order to determine resilience; Urine status is superfluous and only given if strictly indicated. Has no significance for Sports physicians No significance for sports medical counselling. Is also always associated with circumstances and support staff
- Recommendation is more general medical, if necessary supplement with sports medicine; what does this have to do with sport?

**Preventive healthcare service?**

- Still part of preventive care? For me, if necessary means if a disease is suspected. This is then no longer part of preventive care, but as a under clarification of a disease
- At whose expense?
- On request

**Indication**

- When is the indication recommended?; when exactly? more detailed definition
- Targeted with regard to sport and patient; this also depends on the athlete: age, training volume, objectives, etc.; dependent on medical history, age and previous illnesses; possibly age-dependent
- Here it finally doesn't say "should" but "if necessary" - however, it must be clear what justifies the need! Certain age? Certain risk factors in medical history or examination?; if there are corresponding indications or at the patient's request
- for specific questions

**Definition of parameters**

- Which lipid value? Please specify which liver + kidney values?

**Further parameters**

- also a complete blood count, as well as iron status, thyroid status
- Iron metabolism and vitamin D3 are included; in women also iron metabolism; additionally iron balance
- Additional vitamin D status, attention to the risk of osteoporosis in women. Osteoporosis has a higher mortality rate than cardiovascular circulatory diseases. Indicator fractures are wrist and femoral neck or pertrochanteric fractures. Early prevention can save lives.
- If necessary, Vit. D, TSH and iron metabolism
- Thyroid values; TSH, uric acid
- Inflammation values in addition; also a sensitive CRP
- Possibly also determination of CK;
- ⍰ perhaps also lipoprotein (a). Generally determined far too rarely as part of preventive care

**Other parameters**

- I would prefer the HbA1c value instead of the pre-prandial blood glucose; better HbA1c instead of fasting blood glucose; please postprandial - people should still be able to perform on the ergometer afterwards! Why pre-prandial blood glucose status if the professional association for laboratory medicine does not recommend it for healthy people without RF?; As part of a comprehensive examination with ergometry, 2 appointments would be necessary, as otherwise ergometry would have to be performed on an empty stomach. The effort involved is unlikely to be accepted by recreational athletes
- Once in a lifetime also for lipoprotein a; for lipid status, at least the lipoprotein(a) value must be enquired about or determined (is only necessary once in a lifetime).
- Values such as ferritin etc. would be more important than lipid status
- BB, CRP, creatinine, everything else is a question of targeted examinations as SHI
- I consider electrolytes to be dispensable

**Suggested wording**

1. individually, further laboratory values may be of interest (....)
2. "should be carried out"
3. should be a component

**14 Other parameters, e.g. inflammatory markers, renal retention values, should be determined if there is a corresponding clinical suspicion.**

- Sports medicine n=81 positive, n=12 positive with comment, n=13 negative, n=5 unclear, n=76 without comment
- Other specialists: n=3 positive, n=3 positive with comment, n=0 negative, n=2 unclear, n=10 without comment

**Outside of sports medical prevention**

- is not part of the "precautionary measures for those free of complaints". If diseases are suspected, I would recommend that the patient undergo further examinations; in any case, further diagnostics if an existing disease is suspected
- is already diagnostic/curative; diagnostics should always be carried out if a disease is suspected
- but not as part of the sports medicine examination, but by the relevant specialist department; definitely outside of sports medicine!!!; leads too far into sports medical examination for preventive care
- should also be organised via the attending GP; no indication for sports medicine - delegation to the GP's department; should contact the GP.

- alternative: referral / recommendation to specialist colleagues; it makes sense to filter a basic examination and refer on to the specialist colleague; if there is a suspicion, further clarification is required. However, the question arises here as to whether this could also be a recommendation by further
- internal medicine/specialist referral can be made
- does not really need to be mentioned in detail and only as an example here that what is necessary should be done. If there is a suspicion of a disease clarified or a clarification recommended - as is the case everywhere else in medicine
- but only if desired - screening is not diagnostics

**Preference for more (rarely)**

- should always be carried out!
- If a blood count is already done, I think inflammatory markers and renal retention parameters should also be determined as standard
- I would do it at a lower threshold, but with clinical suspicion in any case.

**Parameters**

- Kidney values are already listed above and should remain there, delete here
- Kidney is already mentioned under 13, thyroid values, iron status gladly in addition
- I would always include kidney retention values
- Here I would recommend the ferritin value for ""flabbiness"", I have already pulled out people with values of 2.6 (!!) to 7 who had an odyssey of visits to the doctor, because the blood count was actually ok ... According to my observations, this happens quite often with athletes;
- Lipoprotein a with poor lipid profile or family history @ vascular occlusions/heart etc.
- Creatinine sufficient, inflammation parameters only if indicated or in elite athletes, against CK and troponin in elite athletes
- Specify inflammatory markers: CRP
- add ferritin, TSH, vitamin D here
- or in case of any positive family history / nowadays possibly also VitB12, VitD, Fe or so if very special diets exist (e.g. ultra strict vegan?)
- and other parameters also in case of clin. suspicion

**Suggested wording**

1. ... should ONLY be carried out,…

**15 A 12-lead resting ECG should be recorded as part of the sports medical check-up.**

- Sports physicians: n=84 positive, n=17 positive with comment, n=15 negative, n=4 unclear, n=67 without comment
- Other specialists: n=6 positive, n=2 positive with comment, n=1 negative, n=1 unclear, n=8 without comment
- Comments on children/adolescents omitted as not the target group

**Criticism: not enough**

- significantly worse than an ergometry
- no, a stress ECG !!!
- Either it will or it won't...
- "must" Please don't try to please everyone again with the result that we then have a vague, meaningless "guideline" that doesn't "guide"; I lack the evidence here for the 20-year-old recreational athlete. So far, all guidelines see evidence here only from the age of approx. 40 years. Otherwise, a long-term ECG would be recommended if you really want to find arrhythmias, but this is even more absurd in the care of the masses.

**Criticism: too much**

- What are the benefits for fit young people without risk factors? Money!
- Would then only be able to be carried out by cardiologists and internists. Means too great a restriction of qualified doctors
- Can be proven, but with weak evidence
- Unnecessary in the low prevalence area (positive predictive value too poor)

**Additions**

- ... the first time...., or after infections and inflammatory diseases
- at intervals or at least once every 5-10 years
- at least, more likely with ergometry

**Limitations**

- once and then depending on the intensity of the sport and concomitant illnesses or symptoms - patients are in specialist care with an ECG (in the case of cardiac issues) if they have symptoms.
- according to type of sport and intensity
- only as an initial examination if not yet available and only for the restricted group
- only for suspected cardiological problems; only for red flags
- only depending on age and risk profile, not always necessary, especially not for young recreational athletes for repeat examinations
- only if the medical history indicates a need for an ECG examination
- if there is an indication of pathology and, if possible, a comparative ECG is available

**Execution**

- sitting or lying down?
- under defined conditions (lying down, leg leads close to the foot, not to the pelvis, otherwise an old posterior wall infarction may be overlooked).
- Recording with and without (!!!) filter. If possible, evaluation should be carried out automatically with a "sports programme", such programmes are available (Schiller and Custo) are very helpful in terms of experience, literature is available (e.g. Hilde Berger)

**Suggested wording**

1. ...or more recent.
2. ...if not done otherwise.
1. a 12-lead resting ECG must be written as part of the sports medical check-up and analysed by a specialised doctor
3. "may" / as required
4. strong recommendation
5. strong recommendation, i.e. obligatory (several times)
6. "must" (several times)

**16 Do not routinely perform echocardiography as part of the sports medical check-up.**

- Sports physicians: n=62 positive, n=29 positive with comment, n=21 negative, n=9 unclear, n=66 without comment
- Other specialists: n=6 positive, n=0 positive with comment, n=5 negative, n=0 unclear, n=7 without comment
- Comments on children/adolescents and professional athletes omitted as not the target group

**Preference for a one-time echocardiography**

- Once in a lifetime, repeated in case of complaints
- Echo once (frequently mentioned)
- recommended during the initial examination to detect congenital heart defects

- is assessed differently by cardiologists (1x youth, from 40), but is not widely available. I would prefer a specific optional recommendation
- Once during adolescence and then a second time from the age of 35?

**Preference for routine echocardiography**

- but recommendation / but to be recommended; other recommendations are economically influenced- echo is one of them!
- Transthoracic echocardiography is used to recognise more cardiovascular pathologies that are not/not yet clinically or electrocardiographically conspicuous. These are primarily valvulopathies (bicuspid aortic valves, mitral valve prolapse), aortic dilatations, regional HCMP, ARVC, congenital coronary anomalies
- at least every 2-3 years; routine every 4 years if the recommendation for screening is to do this every 2 years
- Why not, if the examiner has the opportunity based on their own expertise and capabilities

**Indications for echocardiography**

- only for specific questions; only if required; only for corresponding <u>indications</u>. These could be mentioned;
- age-dependent; in young athletes, some things can be recognised that are not noticeable in the ECG; at least from the age of 50; possibly in older athletes over 60 once every 5 years
- only in case of <u>abnormalities and risk profile</u>; in case of corresponding suspicion/risk; (only) in case of hints; only anamnesis-indicated; only in case of abnormalities and previous illnesses; only in the case of clinical suspicion; only in the case of abnormalities, otherwise if necessary on the recommendation of an external specialist; Special examination, in case of abnormal auscultation findings, no preventive service
- only in the case of <u>known heart problems</u>; but can be performed in the case of e.g. vitium cordis
- depending on <u>the type of sport</u>; only once in certain constellations (selected sports and competitive sports)
- for athletes who do <u>intensive/high-intensity</u> sport at least once; but also depends on the intensity of the sporting activity.
- <u>in case of complaints</u>; only in case of complaints or positive family history
- Repeated follow-up checks in the event of <u>findings requiring monitoring</u> or after relevant illnesses

**Resource requirements**

- For an echocardiogram, almost all sports medicine examination centres (except cardiologists!) would have to send the patient on again. This is not feasible and makes it too complicated for athletes to have these examinations carried out.
- Cost issue;
- This would probably go beyond the scope; this would go beyond the scope and is certainly not feasible across the board; not feasible
- Requirements would be too complex in the context of sports medical check-ups;

**Qualification requirements**

- This is also not possible in a normal GP practice, referral to a cardiologist if suspected;
- No sports medicine examination centre can do everything
- Who can really do echo reliably? Or do we all want to rush to the cardiologist for a fee?
- This usually requires a cardiologist (in private practice). We probably do the echo at university furthermore

**Suggested wording**

1. "Not" should be shown in bold or underlined, otherwise it is often overlooked.
2. Echocardiography should only be performed in exceptional cases for special questions. (Negative formulation is often remembered the other way round. Better formulated positively)

3. Echocardiography can be performed as part of the sports medical check-up if necessary
4. Echocardiography can be performed as part of the sports medical check-up.
5. "may be performed in justified individual cases"
6. Echocardiography is not routinely required.
7. Echocardiography should be performed at least once in a lifetime. Recommendation: Role of echocardiography in screening and evaluation of athletes http://orcid.org/0000-0003-3089-1222 David Niederseer et al.

**17 An exercise ECG should be recorded as part of the sports medical check-up if there is an increased cardiovascular risk and/or exercise-induced symptoms.**

---

- Sports physicians: n=69 positive, n=23 positive with comment, n=30 negative, n=3 unclear, n=60 without comment
- Other specialists: n=6 positive, n=0 positive with comment, n=4 negative, n=0 unclear, n=8 without comment
- Comments on children/adolescents and professional athletes omitted as not the target group

**Criticism**

- No. Low evidence; useful yes, but predictive value ultimately more in the area of dice; this is not state of the art!
- Ergometry is unsuitable for detecting CHD!
- No -> if suspected to be necessary-> refer to specialist and SHI; if exercise-induced symptoms are present (A. p.), I would recommend a cardiologist;
- Yes, but then it gets expensive...
- Unfortunately, this is not the case in GP practices.

**Preference for an obligatory exercise ECG**

- A preventive medical check-up attempts to identify diseases, among other things. If we don't examine anything, how can we do that?
- Must; actually always; should always be carried out; <u>necessary for everyone</u>!; for everyone! with intensive loads. A bad tool, but do we have a better one?
- Exercise ECG always, because objectification of the anamnesis;
- I am generally in favour of a stress ECG before starting training, especially for endurance exercise;
- Ergometry (but only useful <u>with</u> <u>exhaustion</u>) for the detection of exercise-induced symptoms or arrhythmias, but then all age groups; an exercise ECG should in my opinion be a fundamental component, especially for endurance athletes.
- An exercise ECG or, if available<u>, better ischaemia imaging</u> or coronary CT
- I think it makes perfect sense, even if the stress ECG is becoming less important in cardiology, especially when it comes to ischaemia diagnosis. However, arrhythmias etc. can sometimes be recognised in the exercise ECG. An absolute must for me.
- In my opinion, not ONLY in the case of increased risk! Depending on the score, this may be underestimated. A stress test is actually part of it; obligatory even without risk factors;
- Compulsory from the <u>age</u> of 40;
- I would recommend it at the very <u>beginning.</u> The controls must be kept individual; I recommend analysing the study situation here. For example, it may make sense to offer a mandatory exercise ECG when an adult wants to exercise for the first time;
- In my experience, <u>at least once</u> for everyone if possible;
- Why not regularly?; why is the group of people restricted? People with unknown high blood pressure, an unknown cardiac arrhythmia, or other asymptomatic findings may not have an increased cardiovascular risk, but still should not have a stress test?
- Carried out or recommended if not possible on site;
- By the way, exercise testing is also useful for training planning (Karvonen formula).

**Additions**

- If necessary, specify <u>which risk factors</u> are meant here; how is "increased cardiovascular risk" defined?

- individually if necessary;
- in the case of increased exertion; ...or high exertion; targeted depending on findings and sporting exertion; and in the case of sports with high exertion, e.g. triathlon marathon high-altitude mountaineering....;
- for symptoms in any case, for risk factors too
- possibly still age-dependent (from the age of 40 even without other risk factors?); additionally for everyone regardless of symptoms over 45 years; in people over 60 always if possible; in older people (>65); in my opinion, "increased cardiovascular risk" would then be interpreted strictly, e.g. generally from the age of 40;
- only in the event of symptoms and only if the cardiologist considers it appropriate. The importance of the exercise ECG has diminished;
- Supplement ... for assessment and training planning for people who are untrained in sports;
- Yes, but with a recommendation for interpretation?
- Better spiroergometry; preferably more frequently in order to derive training recommendations and have a comparison, preferable to spiroergometry;
- not as a routine, only at the request of the athlete or if indicated (list),
- or on external recommendation;
- Special examination, in case of abnormal auscultation findings, not a preventive service;
- The sentence is misleading, as an exercise ECG should also be carried out if there is no increased cardiovascular risk, e.g. so that a heart rate-based training recommendation can be given at all.

**Suggested wording**

1. If there is an increased cardiovascular risk, an exercise ECG should be performed.
2. An exercise ECG should be performed as part of the sports medical check-up.
3. must
4. As part of the sports medical check-up for more than moderate sporting activity or exertion, an exercise ECG and, if necessary, a supplementary echocardiography should be performed if an increased cardiovascular risk and/or exercise-induced risk and/or exercise-induced symptoms are present.
5. "The patient is also recommended to have an exercise ECG if etc..." [whether this takes place in the patient's own practice or by referral is thus open and both are possible. With the recommendation fulfils the guideline. And the recommendation "should" not be made, but the recommendation is made if... ].

**18 Consider using the results of the exercise test to determine cardiorespiratory fitness and to derive training recommendations.**

- Sports physicians: n=82 positive, n=16 positive with comment, n=5 negative, n=7 unclear, n=77 without comment
- Other specialists: n=6 positive, n=1 positive with comment, n=0 negative, n=1 unclear, n=9 without comment

**Criticism**

- This requires more than ergometry
- Is certainly superficial; if it is a pure exercise ECG, then only very vaguely. The decisive factor for athletes >40 is the blood pressure behaviour and ECG abnormalities.
- Not possible in terms of time in a health insurance doctor's practice or the time involved is not paid for.

**Classification**

- Does not belong in the basic examination.
- This in turn is a preventive service and would therefore be part of an extended sports medical check-up including training recommendation.

**Qualification**

- This is only possible with the appropriate expertise.
- requires knowledge

**Additions**

- if done
- then please always with lactate; if necessary with lactate determination;
- As part of spiroergometry or alternatively by means of lactate performance diagnostics!
- Especially if they have been supplemented by a lactate analysis or spiroergometry; (e.g. the spiroergometric values are ideal for this); only if spiroergometry was used;
- Only if performed for a specific sport;
- It is essential that the test is carried out with a complete workload, not limited in time and ideally depending on the type of sport (treadmill, cycle ergometer, rowing ergometer).
- A maximum exercise test (at least BORG 17) should be carried out; the patient should indicate the BORG value at each exertion level; depending on the examination conditions. Test runners on a treadmill, cyclists on a bike with similar geometry to their geometry as their own training equipment

**Suggested wording**

1. The results of the exercise test are used to determine cardiorespiratory fitness and to derive training recommendations.
2. ...can be used for orientation purposes to determine... [In my opinion, an exercise ECG to rule out coronary insufficiency (in which the maximum performance should be reached between 10-14 min. should be reached) is not suitable as a substitute for performance diagnostics. The results are therefore only of limited applicable as a guide]

**Strength of recommendation**

- instead of "can" -> "should" (these are important sports medicine data with high clinical and prognostic relevance and our expertise in sports medicine)
- I would replace "can" with "should"; can? should!
- will! You should not. If no consequences can be derived from the examination then the examination does not need to be recommended; what is that supposed to mean? What does "can" mean here? Ergometry is the basis for training recommendation
- could

**19 Spiroergometry can be part of the sports medical check-up to determine cardiorespiratory** *['cardiorespiratory' added subsequently for clarity]* **fitness and for training recommendations or monitoring.**

---

- Sports physicians: n=66 positive, n=21 positive with comment, n=17 negative, n=11 unclear, n=72 without comment
- Other specialists: n=5 positive, n=2 positive with comment, n=3 negative, n=0 unclear, n=8 without comment
- Comments from professional athletes omitted as not the target group (often referring to competitive sport)

**Criticism 1**

- Too much for regular recreational athletes, in special cases it may make sense; this is an individual additional service and should be formulated differently.
- A spiroergo to determine fitness and for training recommendations/control does not belong in the area of "preventive care"; this is certainly not a service provided by the statutory health insurance funds and also goes well beyond the aim of a preventive check-up; not really a preventive care topic but specific training recommendation; in my opinion, not appropriate as part of preventive care.
- Ultimately too complex; too ambitious, complex and expensive; difficult to realise in practice due to capacity; too little capacity

- Generally not necessary and, above all, can only be implemented in a few places; obsolete nowadays; not necessary
- Only makes limited sense in competitive sports. Lactate performance diagnostics more useful and practicable
- Not wrong. But please think about how many doctors with "sports medicine" qualifications can carry out spiroergometry!
- That it can be done is undisputed. But if this examination is emphasised in the guidelines, then many sports physicians will immediately put the guidelines away...

**Criticism 2**

- This can also be an FMS or a Y-balance test ... if there is no specific need, it is one of many means of training control. Either a formulation should be included here for whom the examination is recommended or the formulation should be kept general "Further examinations and assessments to determine fitness and for training recommendation/control can .... (For example spirometry, spiroergometry, lactate measurement, FMS, Y-balance test .....);
- Whereby, in contrast to recommendation proposal 16 (echocardiography), it is not conclusive to formulate spiroergo as "may" and echo as "should not "routinely". Both as "can in justified individual cases" seems more coherent to me. This also implies that spirometry per se has no indication without exercise
- For initial examinations always.

**Indication**

- When is it indicated? when does it make sense?
- Useful for endurance athletes; helpful as an extended module, especially for intensive, possibly competitive recreational sport; only necessary for counselling at a very high level.
- for specific issues; but only for specific issues; also for specific issues (cardiac/pulmonary) or regarding performance diagnostics possible
- is not a basic examination!
- at the subject's request; it should NOT be an obligatory part of the sports medical check-up, but should be offered as an optional examination; whoever is interested and always pays, otherwise probably only with a corresponding performance profile or for training/load control
- The informative value of the examination is limited. Only for patients with lung problems; I would add that spiroergometry can also be used to visualise circulatory or ventilatory imitations; only in the case of abnormalities and then, if necessary, on the recommendation of external.

**Additions**

- Or lactate performance diagnostics
- Then only to be carried out at institutes

**Further points for discussion.**

- At most a "can" recommendation, as the examination is too specialise
- But should be determined individually, as the chances of getting an appointment are extremely low!
- Spiroergometry/lactate performance diagnostics .... A test with a mask is a deterrent, especially for people who are not (yet) so keen on sport, but it is precisely these people who need values....why the exclusion
- Requirements (equipment, expertise) for this are considerable

**Suggested wording**

1. As part of the sports medical check-up, an exercise ECG or, depending on the issue, a spiroergometry should be carried out. [In conjunction with recommendation 17]

**20 Carry out additional laboratory and instrumental examinations only in individually justified cases.**

- Sports physicians: n=96 positive, n=6 positive with comment, n=3 negative, n=5 unclear, n=77 without comment
- Other specialists: n=8 positive, n=0 positive with comment, n=2 negative, n=1 unclear, n=7 without comment

**Acknowledgement**

- Clear statement with room for interpretation.
- This is not intended to be diagnostic but preventive.
- Would otherwise be too expensive; depending on the setting, the patient could pay for it.
- After referral to a specialist, by the specialist.

**Useful examinations for everyone?**

- Analysing body composition is much better than BMI.
- I find the laboratory important for every examination
- A short pneumo test should be included (FVC or peak flow meter and flow-volume curve if necessary)
- Question about the lung function test - only if there are clinical abnormalities?
- Lung function (spirometry) is missing. This must naturally be an integral part. In sport, the cardiovascular system and the respiratory system and their interaction are of equal importance.
- I consider extended laboratory diagnostics with regard to nutrition (RED-S) or bone status and also instrumental diagnostics, MRI screening etc. to be justifiable, and not just in individual cases.

**Useful for individual**

- Pulmonary function test for ice sports athletes, if necessary
- If necessary, visual acuity test e.g. for marksmen etc.
- e.g. spirometry for chron. respiratory diseases or for diving sports
- Vit. B determination for vegans is important - but who pays for these examinations???? the doctor
- However, I think it makes sense to carry out a useful! extended laboratory diagnosis (e.g. iron status in endurance athletes).
- If there is a suspicion, further equipment examinations are helpful.
- Which ones are meant?

**Suggested wording**

1. conditional recommendation
2. ... can... (optional recommendation)
3. ... are only necessary and should only be carried out in justified individual cases.
4. Additional laboratory and instrumental examinations are carried out in justified individual cases carried out.

**21 Swollen joints, pain, relevant movement restrictions or instability of large joints as well as motion pain or pain on percussion of the spine with or without neurological deficits should be referred for specialist evaluation (specialist in orthopaedics/trauma surgery or physical and rehabilitative medicine).**

- Sports physicians: n=81 positive, n=16 positive with comment, n=14 negative, n=1 unclear, n=75 without comment
- Other specialists: n=7 positive, n=1 positive with comment, n=1 negative, n=2 unclear, n=7 without comment

**Criticism**

- No, rather leads to a sports ban!
- Not useful, not every ankle sprain requires an orthopaedic specialist.
- But unfortunately not feasible in everyday life due to scheduling difficulties. Appointments are pointless after 6 months.

- I don't think it makes sense to mention this in the guidelines. It goes without saying that the examiner will decide during the basic sports medicine examination will and must decide in which speciality the pathological condition merits a follow-up examination. This does not have to be written into the guidelines. This merely patronises the examiner; I also think that colleagues can be trusted colleagues can be trusted to send athletes with instabilities etc. to the appropriate specialists.
- That sounds like "too late" the O&U[8] specialist evaluation should already take place as standard, not only in the case of specialities, as the O&U specialist already notices characteristics of risk profiles that may escape the attention of non-specialist colleagues!
- Why the restriction to individual specialists here. For ECG, echo, ergometry, it did not say that this should be done by internists / cardiologists.
- According to the guideline would be better

**Qualification**

- I don't think it's necessary to specify which specialist the patients should be presented to, and I think this is rather restrictive. If the is a specialist in surgery (but not trauma surgery/orthopaedics) and considers the rheumatological presentation to be more appropriate rheumatological presentation, this would be a violation of the guidelines, which he would have to justify unnecessarily in his documentation based on the wording.
- If the examiner lacks experience; this depends on the examiner's experience; if not in the examiner's own area of expertise definitely yes; if the sports physician does not have the appropriate specialist qualification; a physician with a sports medicine qualification should be able to judge this and only send on if there are important consequences. should be able to judge this and only refer if there are important consequences; a good sports physician can also make statements about the musculoskeletal system. A good sports doctor can also make statements about the musculoskeletal system or it is better not to do the sports medical examination! In most cases the musculoskeletal system is the main focus of the sports medicine examination.
- Yes, but we are all medical specialists; perhaps insert if necessary, as some sports physicians do / initiate the diagnostics themselves or pass it on to the athlete's GP.
- Specialist in orthopaedics and trauma surgery with sports medicine.
- A specialist in rehabilitation medicine is not appropriate here; rather a specialist in orthopaedics/trauma surgery than PM&R[9]
- Other specialisms may also be relevant (rheumatology, endocrinology, imaging...); with neurological limitations; better also neurologist; neurological deficits => evaluation by neurologist; depending on anamnesis ortho or internal medicine, if possible with rheumatology.
- An experienced GP can also clarify this first.
- Can, not must (an orthopaedist is not always better than a GP with experience); even a solid orthopaedic examination is likely to be too much for most sports physicians who are not orthopaedically trained.

**Not only for orthopaedic abnormalities**

- All clinical abnormalities should be referred to a specialist for diagnosis; ALL abnormalities should be clarified further, even if it is the dental status!!!!
- What about other findings that require clarification? It could be formulated more generally that anamnestic or clinical abnormalities may require further clarification by a specialist. It is hardly possible to be more precise, as there are almost unlimited individual case constellations;
- The same should then apply to internal (and possibly other, such as gynaecological) issues; why does this not also apply to internal and other diseases?

**Additions**

- in case of pain or restriction;
- as a recommendation to the patient;

[8] O&U=Orthopädie und Unfallchirurgie, medical specialty area of orthopaedics and trauma surgery
[9] PM&R=preventive medicine and rehabilitation

- unless the complaints are already known and the patients have already been presented there for diagnosis and treatment.

**Suggested wording**

1. Preferably only "specialist evaluation" without specifying (a) discipline(s).
2. Joint swelling, pain, relevant movement restrictions or instability of large joints as well as movement or knocking pain of the spine with or without neurological deficits should be referred to a specific specialist evaluation.
3. Better: persistent ....
4. Musculoskeletal pain (instead of pain)
5. Deletion of "knocking pain"
6. strong recommendation

**22 People with artificial joints or a history of serious injury (e.g. vertebral body fractures, recurrent joint injuries) should be monitored regularly by a specialist.**

- Sports physicians: n=74 positive, n=12 positive with comment, n=30 negative, n=4 unclear, n=75 without comment
- Other specialists: n=7 positive, n=2 positive with comment, n=0 negative, n=2 unclear, n=7 without comment

**Criticism**

- The consultation and assessment by a sports medicine specialist are sufficient in this case as well.
- A doctor who is experienced in sports medicine has learnt to answer this question independently in his additional qualification in sports medicine. If uncertain, they may refer to a specialist, but mandating such referral in guidelines seems misguided. In our practice, we often find that a GP with expertise in sports medicine can provide better information in this area than an orthopaedist who is not well-versed in the topic.
- But not in the context of general practice; often unnecessary and without an evidence base.

**Rationale**

- Why? Either the injury has healed - in which case a regular check-up "only" because of sport is not necessary - or the injury has not healed, then the person is (ideally) being treated by a specialist anyway. A well-fitting endoprosthesis that does not cause any discomfort does not need to be "regularly monitored";
- ...in most cases the patients are already - guidelines relevant?; they are (anyway).

**Depending on complaints**

- If individually required, not routinely; only necessary in individual cases; should be carried out if symptoms occur; depending on medical history
- "Accompanied" is all the rage...follow-up or subsequent examinations may be useful depending on the damage
- I think this is excessive if there are no acute problems; not necessary if there are no symptoms.

**Period**

- Regularly - how often is that; what is meant by "regularly"? At least once a year?; 1x/year; possibly extend intervals if stable over 10 years.

**Further specifications**

- "Serious" injury is formulated too ambiguously, every patient/client with a relevant previous injury, e.g. time off work? incapacity for work? should have a medical specialist contact for advice on taking up sport.

- What does it mean to be accompanied? Specialist means orthopaedic? And what should the specialist then do?

**Qualification**

- We are all specialists; referring to the appropriate specialist.
- Would be good. However, not all orthopaedists do it; the orthopaedist must assess.
- ? A so-called sports physician (non-orthopaedist) should also be able to;
- But GP with an additional qualification in sports medicine and/or manual medicine can also do this; can also be a GP, depending on the doctor's experience; orthopaedists are not always better than GP with experience; doesn't have to be, practically anyone can assess it; can also be done by resourceful GP/double specialists; not mandatory, if symptom-free and healed, the GP or sports physician;
- Physiotherapy in particular.

**Additions**

- Although other illnesses also require regular medical check-ups;
- Do you also write this for e.g. heart patients, diabetics, etc.? I'm just wondering why it is explicitly written here for orthopaedic clinical pictures
- But nothing was written above about internal diseases.
- But of course also with cardiopulmonary risks.

**Suggested wording**

1. ...the appropriate specialist
2. can, not must
3. should be done if symptoms occur

**23 Risk to self and others and possible aggravation of previous injuries should be considered when assessing sports clearance.**

- Sports physicians: n=95 positive, n=3 positive with comment, n=2 negative, n=6 unclear, n=81 without comment
- Other specialists: n=9 positive, n=0 positive with comment, n=0 negative, n=9 without comment

**Communication**

- Previous damage should be clearly communicated to the patient/client, as should the resulting recommendations.

**Shared decision-making**

- The risk should be set in relation to the positive effect of the respective movement and this should be explained.
- Pay attention to "shared decision-making"

**Additions**

- What consequence?
- Important, especially in the case of epilepsy, for example.

**Uncertainties**

- What is the risk of danger to others; what is danger to others? What is self-endangerment in sport?
- Context somewhat unclear. I assume that it is the sport, not the person that poses a risk to themselves and others.

**Criticism**

- Too general; sounds sensible, but is usually not so clear-cut; difficult for the assessor to differentiate.

## Appendix 5. Effect of consumer and sports physicians' surveys on recommendations [10]

| Draft recommendation | Relevant consumer comments (selection) | Relevant sports physicians' comments (selection) | Modification following surveys<br>Final recommendation with strength of consensus in % |
|---|---|---|---|
| 1 Alternative A: Offer a sports medical check-up to adults who want to take up or return to sport.<br>1 Alternative B: Offer a sports medical check-up to adults who do sport. | The separation of the two variants in terms of content is not clear to me - if preventive examinations are justifiable for both (new) beginners and active athletes, a combined recommendation could also be made, focussing on active and future athletes.<br>I think regular examinations are important. For everyone.<br>... who do sport or would like to start in the future... | Adults who do sport, or especially those who are new to sport or want to start again<br>All adults who (want to) take part in sporting activities should be offered sports medicine counselling.<br>who do sport or want to take up sport again....<br>Adults who are new to or returning to sport and adults who regularly take part in sport should be offered a.... | two alternatives combined<br>A PPE should be offered to adults practicing or intending to start sports. (85% consensus, moderate) |
| 2 Alternative A: Use the relevant guidelines for people with chronic diseases.<br>2 Alternative B: Use the relevant guidelines for people with diseases. | Should guidelines not always be used?<br>Shouldn't such guidelines apply to everyone who has such check-ups, regardless of illness, whether chronic or not?<br>Overall unclear objective of the recommendation (use of the guidelines for sports medical check-ups or use of the specific guidelines for the treatment of existing illnesses) - further comment assumes that it is a matter of applying the guidelines for sports medical check-ups. | far too vague<br>too undifferentiated<br>far too general<br>The respective guidelines should always be used for all chronic diseases. Because this is self-evident, this sentence can be removed<br>Which guidelines? Who even knows the guidelines?<br>What is the connection to sports medicine? | no recommendation made, replaced by explanatory text |
| 3 A sports medical check-up should be conducted when starting a more intensive sports and exercise programme | – | – | none (editorial changes only)<br>A PPE should be conducted when starting an intense sport or exercise program. (100% consensus, strong) |
| 4 Alternative A: A sports medical check-up should be repeated regularly.<br>4 Alternative B: A sports medical check-up should be repeated regularly at intervals of 1-3 years. | "Regularly" is too vague: every year? Every 3 years? Every 10 years?<br>Interval specification is good | "Regularly" is too unspecific<br>too little room for individuality with a fixed time interval, not feasible for recreational sports every 1-3 years | two alternatives combined, using specific but wider intervals, focusing on offering rather than conducting a PPE, and added specific criteria:<br>A PPE should be offered at 1–5-year intervals, depending on the individual's risk profile, |

[10] Recommendations were translated with www.DeepL.com/Translator (free version) with manual modifications.

| Draft recommendation | Relevant consumer comments (selection) | Relevant sports physicians' comments (selection) | Modification following surveys<br>**Final recommendation with strength of consensus in %** |
|---|---|---|---|
| | Is the time interval realistic? Presumably more needs-orientated, or every 5 years except for strenuous sports (boxing, marathons, etc.)?<br>Not absolutely necessary, rather on one's own initiative<br>Depends on the patient and their physical condition. If the first examination was unremarkable, I don't think it necessarily has to be repeated or perhaps only every 5 years.<br>The frequency of repetition should be discussed with your doctor depending on risk factors, previous illnesses / injuries and the level of sporting activity (pure recreational sport, ambitious recreational athlete or competitive athlete). | As an offer, only if necessary (e.g. change in state of health), benefit in target population unclear<br>Please be more specific. under what conditions 1, 2 or 3 years? does this depend on age, findings, state of health?<br>A distinction should be made here between types of sport and intensity. Amateur athletes certainly require less intensive examination intervals than high-performance athletes.<br>The older (the athlete), the shorter the time intervals<br>In my opinion, it depends on the findings that need to be checked.<br>depending on the person and the intensity and risk of the sport | performance level, sport type, and intensity. (100% consensus, strong) |
| 5 Also use the results of the sports medical check-up to derive individualised exercise and training recommendations. | – | – | none (editorial changes only)<br>Use the PPE results to derive individualized exercise and training recommendations. (100% consensus, strong) |
| 6 Evidence-based communication strategies should be used to convey exercise recommendations. | I can't make much sense of that. Sounds too technical to me.<br>Makes sense, but the goal and the way to get there should still be communicated clearly and comprehensibly. | A meaningless sentence. What are "evidence-based communication strategies"? Sounds esoteric. With the exception of a small group of people, nobody here knows what is meant by this. People will only agree because it says "evidence-based". Phrase it more clearly or remove it.<br>And above all, evidence-based training concepts<br>The communication of exercise recommendations should be adapted to the individual situation and take into account the positive and negative biopsychosocial factors. (motivation, available time, workload, social involvement in sports clubs/groups, pre-existing conditions, etc.). | expanded concept, inclusion of goal:<br>Techniques to support behavioral change should be used when communicating exercise recommendations (100 % consensus, strong). |

| Draft recommendation | Relevant consumer comments (selection) | Relevant sports physicians' comments (selection) | Modification following surveys<br><br>**Final recommendation with strength of consensus in %** |
|---|---|---|---|
| 7 Alternative A: A sports medical check-up should be carried out by a specialised doctor, preferably with certification in sports medicine.<br><br>7 Alternative B: A sports medical check-up should be carried out by a specialised doctor with certification in sports medicine. | – | – | none (editorial changes only)<br><br>A PPE should be conducted by a specialized physician with an additional qualification in sports medicine. (80% consensus, moderate) |
| 8 A standardised medical history form should be used as part of the sports medical check-up. This should include the following items:<br><br>• personal and family medical history<br>• individual risk profile<br>• participation in earlier check-ups and their results<br>• information on surgical procedures and injuries<br>• nutritional history<br>• gynaecological history<br>• vaccination status | Use of medication (influence)<br><br>intake of WADA[11]-listed products (today/past). | Supplements/medications.<br><br>Taking medication (including the pill) and dietary supplements and painkillers!<br><br>Medication history, especially use of painkillers<br><br>Medication status, especially lifestyle products | addition of item:<br><br>A standardized medical history form should be used in the PPE. It should include:<br><br>• personal and family medical history<br>• sports history<br>• individual risk-factor profile<br>• *medication history*<br>• nutritional history<br>• gynecological history<br>• vaccination status<br>• participation and results of previous check-ups<br>• previous injuries and/or surgeries.<br><br>(100% consensus, strong) |
| 9 A validated score should be used to assess the cardiovascular risk. | It depends on the criteria. Not every score makes sense...<br><br>Okay, but currently not assessable as long as there are no proposals for scores.<br><br>I can't imagine that such a value can be found and that it is meaningful. | Preferably with an example<br><br>this should be specified<br><br>A list of suitable scores should be provided here.<br><br>yes. but specifically which one ???<br><br>Suggestion: Arriba.<br><br>Yes (SCORE2 or SCORE2-OP). | addition of examples for the score:<br><br>Use a validated score *(e.g., Arriba and SCORE2)* to assess cardiovascular risk from the age of 35 years. (100% consensus, strong) |
| 10 A sports history should include questions about<br><br>• the frequency, intensity, duration and types of sport | Isn't the goal and training plan missing here? | plus the patient's / athlete's objective (return to sport, ..., competitions, ...)<br><br>What is the aim for the future? | addition of item:<br><br>A sports history should include questions about the following factors: |

[11] World Anti Doping Agency

| Draft recommendation | Relevant consumer comments (selection) | Relevant sports physicians' comments (selection) | Modification following surveys<br><br>Final recommendation with strength of consensus in % |
|---|---|---|---|
| • previous experience with sport<br>• environmental conditions during sport, such as heat, cold or altitude<br>• complaints at rest and during exercise<br>• individual sport-related risk profile and<br>• any aids or restrictions (e.g. visual aids) | | Goals of the sporting activity? Intensification planned? possibly participation in a marathon or similar? | • frequency, intensity, time, and type (FITT)<br>• previous sporting experience<br>• <u>*goals of the physical activity*</u><br>• environmental conditions during the sport (e.g., heat, cold, or altitude)<br>• complaints at rest and during exercise<br>• individual sport-related risk profile<br>• aids (e.g. visual aids) or mobility restrictions. (100% consensus, strong) |
| 11 Obtain a whole-body assessment as part of the sports medical check-up:<br><br>• Collection of anthropometric data (height, weight, abdominal girth, calculation of body mass index)<br>• Pulse and blood pressure measurement at rest<br>• Examination of heart and lungs<br>• Assessment of vascular status<br>• Abdominal examination<br>• Examination of the musculoskeletal system including a) mobility and stability of the large joints b) mobility and tapping pain of the spine c) muscle status / function, tone, etc. Examination of the nervous system (gait pattern, reflex status, sensitivity, cranial nerve status if applicable)<br>• Examination of the sensory organs (incl. visual acuity assessment with eye charts)<br>• Inspection of the skin<br>• Inspection of the oral cavity, recording of dental status<br>• Assessment of lymph node status | (substantial variation in preferences for individual parameters)<br><br>appears to be very extensive<br><br>Here I would adapt the scope of the examination to the athlete's profile.<br><br>Depending on the frequency of the repeat examination, the scope can be reduced or extended depending on risk factors (previous) illnesses/injuries and the level of sporting activity<br><br>BMI is sometimes misleading for athletic/very muscular people<br><br>Could the BMI be replaced/supplemented by more helpful parameters, e.g. body fat percentage?<br><br>Urinalysis, rule out urinary tract infection. Bladder ultrasound, exclude residual urine formation. Incontinence?<br><br>Why should the mouth area be examined during a sports medicine check-up? Why an inspection of the skin?<br><br>But this is expensive and time-consuming. | (substantial variation in preferences for individual parameters)<br><br>Insofar as specifications are made, I consider differentiated information to be useful here<br><br>There is certainly a lot to be trimmed from the list. This scheme cannot be used for all age groups as it is far too extensive and time-consuming. Not realisable in practice.<br><br>Each item can be useful under certain circumstances. But - "reduce to the max!". A child wants to take part in canoeing competitions. Do they really need a "vascular status"? Surely some combination is theoretically conceivable where the status may be needed. But the whole thing has to remain manageable and not cover every possibility in principle. A sports physician can choose (!!) what he needs. Perhaps a template where you can tick a box? But not everything has to be ticked?<br><br>the orthopaedic examination could be more detailed with more room for additions | moved details to supporting text:<br><br>Determine whole-body status as part of the PPE, which should be based on the recommendations in Table 2. (100% consensus, strong) |
| 12 Consider measuring muscle strength as part of the sports medical check-up. | Why?<br><br>For what purpose?<br><br>Which muscles? Doesn't necessarily make sense for every sport.<br><br>Hand strength measurement | But how and why?<br><br>but expanding here: which strength tests? Which ones/goals? Either it is a specific and in-depth examination or it does not need to be listed separately as it is (not should be) included in the whole body status; | included aim and example of test:<br><br>Muscle strength can be determined <u>*to measure muscular fitness*</u> <u>*(e.g., hand grip strength*</u>) as part of a PPE. (100% consensus, strong) |

| Draft recommendation | Relevant consumer comments (selection) | Relevant sports physicians' comments (selection) | Modification following surveys<br>**Final recommendation with strength of consensus in %** |
|---|---|---|---|
| | | Determine how and which muscle strength should be measured<br>Handgrip is sufficient in my opinion | |
| 13 A full blood count, pre-prandial blood glucose concentration and lipid status, liver/kidney values, electrolyte concentration and urine status can also be determined if necessary. | – | instead of the pre-prandial BG, I would prefer the HbA1c value<br>better HbA1c instead of fasting blood glucose<br>please post-prandial - people should still perform well on the ergometer afterwards! | modified glucose test selection:<br>If necessary, a blood count, *plasma glucose, HbA1c* and lipid status, liver/kidney values, electrolyte concentration, and urine status can be determined as part of a PPE. (100% consensus, strong) |
| 14 Other parameters, e.g. inflammatory markers, renal retention values, should be determined if there is a corresponding clinical suspicion. | – | No sports medicine indication - referral to general practitioners<br>is not part of the "prevention for the asymptomatic person". If diseases are suspected, I would recommend that the patient arrange for further examinations<br>If a disease is suspected, it should be examined or an investigation recommended - as is the case everywhere else in medicine | no recommendation made, content covered by recommendation no. 20 |
| 15 A 12-lead resting ECG should be recorded as part of the sports medical check-up. | – | – | none (editorial changes only)<br>A 12-lead resting ECG should be performed as part of each PPE.(100% consensus, strong) |
| 16 Do not routinely perform echocardiography as part of the sports medical check-up. | – | – | none (modified following discussion during the consensus conference):<br>Perform an echocardiogram as part of a PPE if there is reason to suspect structural heart disease. (90% consensus, moderate) |
| 17 An exercise ECG should be recorded as part of the sports medical check-up if there is an increased cardiovascular risk and/or exercise-induced symptoms. | –<br>Contrary to the changes made, consumer preference was in favour of a stress ECG even in the absence of increased risk. | (substantial variation in preferences in favour or against the exercise ECG)<br>in case of symptoms definitely, in case of risk factors also<br>If necessary, specify which risk factors are meant here; how is "increased cardiovascular risk" defined?<br>Targeted depending on findings and exercise load | specified conditions for the test:<br>An exercise ECG should be performed as part of a PPE *if warranted by the PPE findings, the individual risk profile, possible exercise-induced symptoms, the type of sport in question, and the performance level and intensity.* (100% consensus, strong) |

| Draft recommendation | Relevant consumer comments (selection) | Relevant sports physicians' comments (selection) | Modification following surveys<br>**Final recommendation with strength of consensus in %** |
|---|---|---|---|
| 18 Consider using the results of the exercise test to determine cardiorespiratory fitness and to derive training recommendations. | – | – | none (recommendation grade lowered following discussion during the consensus conference)<br>A CPET can be used to determine cardiorespiratory fitness and provide training recommendations/guidance as part of a PPE. (100% consensus, strong) |
| 19 Spiroergometry can be part of the sports medical check-up to determine fitness and for training recommendations or monitoring. | –<br>Contrary to the non-recommendation of routine spiroergometry, consumer preference was in favour of a spiroergometry for everyone. | A spiroergo to determine fitness and for training recommendation/control does not belong in the area of "prevention"<br>generally not necessary and, above all, can only be implemented in a few places<br>obsolete today | no recommendation made, replaced by explanatory text |
| 20 Carry out additional laboratory and instrumental examinations only in individually justified cases. | – | – | none (editorial changes only)<br>Perform laboratory and instrumental examinations as part of a PPE that go beyond Recommendations 13–18 only in justified individual cases. (100% consensus, strong) |
| 21 Swollen joints, pain, relevant movement restrictions or instability of large joints as well as motion pain or pain on percussion of the spine with or without neurological deficits should be referred for specialist evaluation (specialist in orthopaedics/trauma surgery or physical and rehabilitative medicine). | – | – | none (editorial changes only)<br>Patients with joint swelling, joint pain, relevant range-of-motion restrictions or large joint instability, as well as movement or knocking pain in the spine (with or without neurological deficits), should be referred to a specialist for further evaluation (preferably a specialist in orthopedics/trauma surgery or physical and rehabilitative medicine). (100% consensus, strong) |
| 22 People with artificial joints or a history of serious injury (e.g. vertebral body fractures, recurrent joint injuries) should be monitored regularly by a specialist. | – | –<br>Contrary to the recommendation, assessment by a sports physician was often considered sufficient | addition of area of specialisation:<br>Individuals with arthroplasties or serious injuries in their medical history (e.g., vertebral fractures or severe/recurrent joint injuries) should be regularly monitored by a specialist, *preferably in orthopedics/trauma surgery or* |

| Draft recommendation | Relevant consumer comments (selection) | Relevant sports physicians' comments (selection) | **Modification following surveys**<br>**Final recommendation with strength of consensus in %** |
|---|---|---|---|
| | | | *physical and rehabilitative medicine*. (91% consensus, moderate) |
| 23 Risk to self and others and possible aggravation of previous injuries should be considered when assessing sports clearance. | – | – | none (editorial changes only)<br>During the PPE and counseling, assess each individual's risk of danger to themselves and others and consider the possible worsening of previous injuries. (100% consensus, strong) |


1. McKay, A.K.A., et al., *Defining Training and Performance Caliber: A Participant Classification Framework.* Int J Sports Physiol Perform, 2022. **17**(2): p. 317-331.
