# Supplementary material for: Sports Pre‐Participation Evaluation in Healthy Adults—A Survey‐Based Assessment of Guideline Acceptability and Feasibility Among Sports Physicians and Participants
Source: Clin Public Health Guidel. 2025 Aug 20;2(4):e70032. doi: 10.1002/gin2.70032 (PMC13563407; doi:10.1002/gin2.70032)
Supplement: Supplementary file 2 [file GIN2-2-e70032-s002.pdf]

Survey on sports pre-participation evaluations

Information about the survey [embedded video]

Video transcript, translated into English:

Do you do sport or are you thinking about starting? Then we would ask you to take about 30 minutes of your time and take part in our survey on sports pre-participation evaluations.

A sports pre-participation evaluation is a medical examination that is specially tailored to the needs of people who do sport or want to start doing sport. The aim is to identify possible risks and make your physical activity as safe as possible. This can result in training recommendations.

The survey is being conducted as part of the development of a guideline for sports pre-participation evaluations. A guideline is a set of instructions that your doctor can use to ensure the best possible medical care. Guidelines are drawn up by experts and contain systematically developed recommendations. They indicate which medical measures are best suited to recognising certain health risks, detecting, preventing, or treating illnesses. The main purpose of the guideline on sports pre-participation evaluations is to provide recommendations on who should consider undergoing such an examination and what should be done during the examination. It is important for the development of the guideline to take into account the opinions of people who may undergo such an examination. By taking part in the survey, you can influence the development of the guideline to reflect your needs and make an important contribution to improving healthcare.

Information about the survey

Do you practise sport or do you consider to (re)start practising? Then we would ask you to take about 30 minutes of your time and take part in our survey on sports pre-participation evaluations.

A sports pre-participation evaluation is a medical examination that is specifically tailored to the needs of people who practise sport or want to take up sport. Its aim is to identify possible risks and make your physical activity as safe as possible.

The survey is conducted as part of the development of a guideline for sports pre-participation evaluation. A guideline is an set of instructions that your doctor can use as a guide to ensure the best possible medical care. Guidelines are written by experts and contain systematically developed recommendations. They indicate which medical procedures are best suited to recognising certain health risks and detecting, preventing or treating illnesses. The guideline on sports pre-participation evaluation primarily aims to provide recommendations on who is eligible for such an examination and what should be done during the examination. For the preparation of the guideline, it is important to take into account the views of people who may undergo such an examination. By taking part in the survey, you can influence the development of the guideline to reflect your needs and make an important contribution to improving healthcare.

Information on data protection

The research data collected will be used exclusively for the development of the guideline and for scientific purposes. The research data is anonymous so that individuals cannot be identified. It is not possible to draw conclusions about the study participants. The research data only includes information that you provide to us directly via the fields in the online survey. This data is only accessible to individuals involved in the analysis.

The results of this study may be published for teaching and research purposes (articles in scientific journals). This will be done anonymously. In order to ensure anonymity, we ask you not to provide any data in the free text responses that would make you clearly recognisable as a person (identifying data such as name or e-mail address).

Information on participation

Participation in this study is voluntary and is aimed at people aged 18 and older. Non-participation has no disadvantages for you. You can cancel the survey before submitting the questionnaire without giving reasons. In this case, your response will not be used.

Please send any questions to the following e-mail address: leitlinien-ifom@uni-wh.de

This survey contains 40 questions.

Personal questions

How old are you?

Please enter your answer here:

This and all further information is voluntary.

What gender do you identify with?

Comment when selecting an answer.
Please select an option and provide your comments.

- ☐ male
- ☐ female
- ☐ other
- ☐ own specification
- ☐ no information

Please select only one option

What is your height? (in cm)

Please enter your answer here:

What is your current weight? (in kg)

What type of health insurance do you have?

Please select only one of the following options:

- ◯ Statutory insurance
- ◯ private insurance
- ◯ other insurance
- ◯ No comment

Do you have a chronic illnes?

Please select only one of the following options:

- ◯ yes
- ◯ no
- ◯ no comment

It is:

Only answer this question if the following conditions are fulfilled:
Answer was 'yes' to question ' [G1Q6]' (Do you have a chronic illness?)

Please select only one of the following options:

- ◯ A disease of the cardiovascular system (e.g. heart attack, heart disease, vascular disease)
- ◯ A disease of the respiratory tract (e.g. asthma, lung disease, chronic obstructive pulmonary disease (COPD))
- ◯ A metabolic disease (e.g. diabetes, obesity)
- ◯ A disease or injury of the musculoskeletal system (e.g. rheumatism, osteoporosis, arthrosis)
- ◯ Another disease, namely (please add in the comment field)
- ◯ No comment

Please write a comment on your selection:

Where would you most likely categorise yourself at the moment?

Please select only one of the following options:

- ◯ Rarely or never active in sports
- ◯ Active in your free time (at least 150 minutes at a relaxed intensity or 75 minutes at a high intensity per week AND muscle-strengthening activities at least twice a week)
- ◯ Trained (regular training approx. 3 times a week, focussing on a specific sport)
- ◯ Highly trained (training and competitions at national level)
- ◯ Elite (training and competitions at international level)
- ◯ No comment

Do you intend to:

Please select only one of the following options:

- ◯ (Re)start with practising sports?
- ◯ Maintain your sporting level?
- ◯ Exercise more often?
- ◯ Increase the intensity of your sport?
- ◯ Participate in sporting competitions/tournaments (e.g. triathlon, company run, football tournament)?
- ◯ No Comment

Have you ever had a sports medical examination? (this refers to sports pre-participation evaluation, not fitness tests or examinations for injuries)

Please select only one of the following options:

- ◯ yes
- ◯ no
- ◯ no comment

Your opinion on the sports pre-participation evaluation

With the following questions, we would like to gather your personal opinion on a sports pre-participation evaluation in general. In order to be able to answer the questions, it is important that you know what is done during a sports pre-participation evaluation and what its aim is. For this, you can watch the video or read the information texts for the individual questions.

What is a sports pre-participation evaluation [embedded video]

Video transcript, translated into English:

A sports pre-participation evaluation is a medical examination. It is specifically tailored to the needs of people who do sport or want to start doing sport. The examination can be carried out by a doctor who has undergone further training on the subject and holds the additional qualification of "sports medicine".

The aim of the sports pre-participation evaluation is to identify possible risks, such as injuries or cardiovascular problems. The aim is to make sport as safe as possible. Your doctor will ask you various questions and carry out tests to assess your health and physical fitness. These include, for example, a physical examination, an ECG of your heart and, if necessary, a blood test. You will also be asked questions about your medical history, your diet, your lifestyle and your physical activity. This will give your doctor a comprehensive picture of your health. Based on the results of the examination, he or she can give you recommendations on how you can organize your physical activity and avoid injuries. For example, if you have had an injury or have a chronic illness, your doctor may recommend that you avoid a certain type of sport or choose an alternative that is less strenuous for your body. If a disease is suspected during the examination, you may be referred to other specialists (e.g. cardiology) for additional tests.

Currently, a sports pre-participation evaluation is not covered by statutory health insurance. Nevertheless, many health insurance companies cover all or part of the costs. Depending on the scope of the examination, the examination costs amount to approx. 70-200€. You can find out directly from your health insurance company whether and to what extent your health insurance covers the costs. The health insurance company will of course cover any follow-up examinations by specialists.

To what extent to you agree with the following statements?

Please select the appropriate answer for each item:

	disagree	partially disagree	undecided	partially agree	agree
I would have a PPE done.<br>Read information	○	○	○	○	○
I would accept the burden involved in a PPE (e.g. blood sampling, emotional stress caused by test results).<br>Read information	○	○	○	○	○
I would be able to take the individual tests of the PPE without any problems.<br>Read information	○	○	○	○	○
The effort associated with PPE would be feasible form me (travelling, time required, other).<br>Read information	○	○	○	○	○
I understand the purpose of a PPE.<br>Read information	○	○	○	○	○

I have comments on the PPE:

Please insert your answer here:

A PPE would help to diagnose or rule out health conditions relevant to exercising. Read information	◯	◯	◯	◯	◯
A PPE would help me to choose the most appropriate form and intensity of physical activity. Read information	◯	◯	◯	◯	◯
I would only go for a PPE if it is reimbursed by the health insurance company. Read information	◯	◯	◯	◯	◯
I would have concerns or reservations about having a PPE done.	◯	◯	◯	◯	◯

Your opinion on the proposed recommendations

In the last part of the survey you will find the provisional recommendations for the guideline on sports pre-participation evaluation. This means that, as things stand at present, your doctor would carry out the examination on you in accordance with these recommendations.

You now have the opportunity to give your opinion on the provisional recommendations and thus influence these. Please select one or more subjects on which you would like to read and comment on. Imagine that you are taking part in sports pre-participation evaluation.

How would you feel if you were examined in accordance with the recommendation?

For instance, you can consider the following aspects in the comments:

- Is the recommendation acceptable to you or do you have any concerns? If so, what are they?
- Do you find the recommendation clear and considerate?
- Does the recommendation leave room for personal particularities, e.g. a physical limitation?
- Are you missing something? Would you like to add something?

In some cases, two variants are suggested for a recommendation. Here you have the opportunity to indicate in the comments field which you prefer and why.

Once the survey has been completed, the survey results are edited for the experts who prepared the recommendations. Based on the survey results, the recommendations are then revised as necessary.

For which subjects would you like to comment on the recommendations?

Please select all applicable answers :

- ☐ General recommendations (e.g. frequency of examination, 7 recommendations)
- ☐ Personal medical history, ‘anamnesis’ (3 recommendations)
- ☐ Physical examination and laboratory (4 recommendations)
- ☐ Cardiovascular and other examinations (6 recommendations)
- ☐ Referral to specialists, suitability for sport (3 recommendations)

Multiple selection is possible.

1 Alternative A:

Offer a sports preparticipation evaluation to adults who want to take up or return to sport.

Only answer this question if the following conditions are fulfilled:
Answer was “General recommendations” (e.g. frequency of examination, 7 recommendations)' for question '[G3Q0]' (For which subjects would you like to comment on the recommendations?)

Please insert your answer here:

Please use the field for comments or suggestions for changes to the recommendation:

1 Alternative B:

Offer a sports preparticipation evaluation to adults who do sport.

Only answer this question if the following conditions are fulfilled:
((G3Q0_1.NAOK == "Y"))

Please insert your answer here:

Please use the field for comments or suggestions for changes to the recommendation:

2 Alternative A:

Use the relevant guidelines for people with chronic diseases.

Only answer this question if the following conditions are fulfilled:
Answer was “General recommendations” (e.g. frequency of examination, 7 recommendations)' for question '[G3Q0]' (For which subjects would you like to comment on the recommendations?)

Please insert your answer here

Please use the field for comments or suggestions for changes to the recommendation:

2 Alternative B:

Use the relevant guidelines for people with diseases.

Only answer this question if the following conditions are fulfilled:
Answer was “General recommendations” (e.g. frequency of examination, 7 recommendations)' for question '[G3Q0]' (For which subjects would you like to comment on the recommendations?)

Please insert your answer here:

Please use the field for comments or suggestions for changes to the recommendation:

3 Alternative A:

A sports preparticipation evaluation should be conducted when starting a more intensive sports and exercise programme.

Only answer this question if the following conditions are fulfilled:
Answer was “General recommendations” (e.g. frequency of examination, 7 recommendations)' for question '[G3Q0]' (For which subjects would you like to comment on the recommendations?)

Please insert your answer here

Please use the field for comments or suggestions for changes to the recommendation:

4 Alternative A:

A sports preparticipation evaluation should be repeated regularly.

Only answer this question if the following conditions are fulfilled:
Answer was “General recommendations” (e.g. frequency of examination, 7 recommendations)' for question '[G3Q0]' (For which subjects would you like to comment on the recommendations?)

Please insert your answer here:

Please use the field for comments or suggestions for changes to the recommendation:

4 Alternative B:

A sports preparticipation evaluation should be repeated regularly at intervals of 1-3 years

Only answer this question if the following conditions are fulfilled:
((G3Q0_1.NAOK == "Y"))

Please insert your answer here:

Please use the field for comments or suggestions for changes to the recommendation:

5:

The results of the sports preparticipation evaluation should also be used to derive individualised exercise and training recommendations.

Only answer this question if the following conditions are fulfilled:
Answer was “General recommendations” (e.g. frequency of examination, 7 recommendations)' for question '[G3Q0]' (For which subjects would you like to comment on the recommendations?)

Please insert your answer here:

Please use the field for comments or suggestions for changes to the recommendation

6:

Evidence-based communication strategies should be used to convey exercise recommendations.

Only answer this question if the following conditions are fulfilled:
Answer was “General recommendations” (e.g. frequency of examination, 7 recommendations)' for question '[G3Q0]' (For which subjects would you like to comment on the recommendations?)

Please insert your answer here:

Please use the field for comments or suggestions for changes to the recommendation:

7 Alternative A:

A sports preparticipation evaluation should be carried out by a specialised doctor, preferably with certification in sports medicine.

Only answer this question if the following conditions are fulfilled:
Answer was “General recommendations” (e.g. frequency of examination, 7 recommendations)' for question '[G3Q0]' (For which subjects would you like to comment on the recommendations?)

Please insert your answer here:

Please use the field for comments or suggestions for changes to the recommendation:

7 Alternative B:

A sports preparticipation evaluation should be carried out by a specialised doctor with certification in sports medicine.

Only answer this question if the following conditions are fulfilled:
((G3Q0_1.NAOK == "Y"))

Please insert your answer here:

Please use the field for comments or suggestions for changes to the recommendation:

8:

A standardised medical history form should be used as part of the sports preparticipation evaluation. This should include the following items:

- Personal and family medical history
- Individual risk profile
- Participation and results of previous check-ups
- Information on surgical procedures and injuries
- Nutritional history
- gynaecological history
- Vaccination status

Only answer this question if the following conditions are fullfiled:
Answer was 'personal medical history, anamnesis, (3 recommendation)' for question ' [G3Q0]' (For which subjects would you like to comment on the recommendations?)

Please insert your answer here:

Please use the field for comments or suggestions for changes to the recommendation:

9:

A validated score should be used to assess the cardiovascular risk.

Only answer this question if the following conditions are fulfilled::
Answer was 'personal medical history, anamnesis, (3 recommendation)' for question ' [G3Q0]' (For which subjects would you like to comment on the recommendations?)

Please insert your answer here:

Please use the field for comments or suggestions for changes to the recommendation:

10:

A sports history should include questions about:

- The frequency, intensity, duration and types of sport
- Previous experience with sport
- Environmental conditions during sport such as heat, cold or altitude
- Complains at rest and during exercise
- Individual sport-related risk profile
- Any aids or restrictions (e.g. visual aids)

Only answer this question if the following conditions are fulfilled:

Answer was ‘personal medical history, anamnesis, (3 recommendation)' for question ' [G3Q0]' (For which subjects would you like to comment on the recommendations?)

Please insert your answer here:

Please use the field for comments or suggestions for changes to the recommendation:

11:

Obtain a whole-body assessment as part of the sports preparticipation evaluation:

- Collection of anthropometric data (height, weight, abdominal girth, calculation of body mass index)
- Pulse and blood pressure measurement at rest
- Examination of the heart and lungs
- Assessment of the vascular status
- Abdominal examination
- Examination of the musculoskeletal system including a) mobility and stability of the large joints b) mobility and tapping pain of the spine c) muscle status / function, tone etc.), Examination of the nervous system (gait pattern, reflex status, sensitivity, cranial nerve status if necessary)
- Examination of the sensory organs (including visual acuity assessment with eye charts)
- Inspection of the skin
- Inspection of the oral cavity, assessment of the dental status
- Assessment of the lymph node status

Only answer this question if the following conditions are fulfilled:

Answer was ‘physical examination and laboratory (4 recommendations)' for question ' [G3Q0]' (For which subjects would you like to comment on the recommendations?)

Please insert your answer here:

Please use the field for comments or suggestions for changes to the recommendation:

12:

Consider measuring muscle strength as part of the sports preparticipation evaluation.

Only answer this question if the following conditions are fulfilled:

Answer was ‘physical examination and laboratory (4 recommendations)' for question ' [G3Q0]' (For which subjects would you like to comment on the recommendations?)

Please insert your answer here:

Please use the field for comments or suggestions for changes to the recommendation:

13:

A full blood count, pre-prandial blood glucose concentration and lipid status, liver/kidney values, electrolyte concentration and urine status can also be determined if necessary.

Only answer this question if the following conditions are fulfilled:

Answer was ‘physical examination and laboratory (4 recommendations)' for question ' [G3Q0]' (For which subjects would you like to comment on the recommendations?)

Please insert your answer here:

Please use the field for comments or suggestions for changes to the recommendation:

14:

Other parameters, e.g. inflammatory markers, renal retention values, should be determined if there is a corresponding clinical suspicion.

Only answer this question if the following conditions are fulfilled:

Answer was ‘physical examination and laboratory (4 recommendations)' for question ' [G3Q0]' (For which subjects would you like to comment on the recommendations?)

Please insert your answer here:

Please use the field for comments or suggestions for changes to the recommendation:

15:

A 12-lead resting ECG should be recorded as part of the sports preparticipation evaluation.

Only answer this question if the following conditions are fulfilled:

Answer was cardiovascular and other examinations (6 recommendations)' for question ' [G3Q0]' (For which subjects would you like to comment on the recommendations?)

Please insert your answer here:

Please use the field for comments or suggestions for changes to the recommendation:

16:

Do not routinely perform echocardiography as part of the sports preparticipation evaluation.

Only answer this question if the following conditions are fulfilled:

Answer was cardiovascular and other examinations (6 recommendations)' for question ' [G3Q0]' (For which subjects would you like to comment on the recommendations?)

Please insert your answer here

Please use the field for comments or suggestions for changes to the recommendation:

17:

An exercise ECG should be recorded as part of the sports preparticipation evaluation if there is an increased cardiovascular risk and/or exercise-induced symptoms.

Only answer this question if the following conditions are fulfilled:

Answer was cardiovascular and other examinations (6 recommendations)' for question ' [G3Q0]' (For which subjects would you like to comment on the recommendations?)

Please insert your answer here:

Please use the field for comments or suggestions for changes to the recommendation:

18:

Consider using the results of the exercise test to determine cardiorespiratory fitness and to derive training recommendations.

Only answer this question if the following conditions are fulfilled:

Answer was cardiovascular and other examinations (6 recommendations)' for question ' [G3Q0]' (For which subjects would you like to comment on the recommendations?)

Please insert your answer here:

Please use the field for comments or suggestions for changes to the recommendation:

19:

Spiroergometry can be part of the sports preparticipation evaluation to determine fitness and for training recommendations or monitoring.

Only answer this question if the following conditions are fulfilled:

Answer was cardiovascular and other examinations (6 recommendations)' for question ' [G3Q0]' (For which subjects would you like to comment on the recommendations?)

Please insert your answer here:

Please use the field for comments or suggestions for changes to the recommendation:

20:

Carry out additional laboratory and instrumental examinations only in individually justified cases.

Only answer this question if the following conditions are fulfilled:

Answer was cardiovascular and other examinations (6 recommendations)' for question ' [G3Q0]' (For which subjects would you like to comment on the recommendations?)

Please insert your answer here:

Please use the field for comments or suggestions for changes to the recommendation:

21:

Swollen joints, pain, relevant movement restrictions or instability of large joints as well as motion pain or pain on percussion of the spine with or without neurological deficits should be referred for specialist evaluation (specialist in orthopaedics/trauma surgery or physical and rehabilitative medicine).

Only answer this question if the following conditions are fulfilled:

Answer was Referral to specialists, suitability for sport (3 recommendations)' for question ' [G3Q0]' (For which subjects would you like to comment on the recommendations?)

Please insert your answer here:

Please use the field for comments or suggestions for changes to the recommendation:

22:

People with artificial joints or a history of serious injury (e.g. vertebral body fractures, recurrent joint injuries) should be monitored regularly by a specialist.

Only answer this question if the following conditions are fulfilled:

Answer was Referral to specialists, suitability for sport (3 recommendations)' for question ' [G3Q0]' (For which subjects would you like to comment on the recommendations?)

Please insert your answer here:

Please use the field for comments or suggestions for changes to the recommendation:

23:

Risk to self and others and possible aggravation of previous injuries should be considered when assessing sports clearance.

Only answer this question if the following conditions are fulfilled:

Answer was Referral to specialists, suitability for sport (3 recommendations)' for question ' [G3Q0]' (For which subjects would you like to comment on the recommendations?)

Please insert your answer here:

Please use the field for comments or suggestions for changes to the recommendation:

30.10.2023 – 06:50

Send in your survey

Thank you for participating.
