# Supplementary material for: Sports Pre‐Participation Evaluation in Healthy Adults—A Survey‐Based Assessment of Guideline Acceptability and Feasibility Among Sports Physicians and Participants
Source: Clin Public Health Guidel. 2025 Aug 20;2(4):e70032. doi: 10.1002/gin2.70032 (PMC13563407; doi:10.1002/gin2.70032)
Supplement: Supplementary file 3 [file GIN2-2-e70032-s003.pdf]

Feasibility of sports preparticipation evaluations from the perspective of sports physicians

Are you a physician? Do you have the additional qualification 'Sports Medicine'? If so, we would like to ask you to take around 30 minutes of your time and take part in our survey on sports medicine screening.

The survey is being conducted as part of the development of an S2k guideline on 'Sports preparticipation evaluations'. The German Society for Sports Medicine and Prevention (DGSP) is in charge of drawing up the guideline with the participation of representatives from numerous other specialist societies. The recommendations of the guideline are currently in proposal status. Before the recommendations are voted on as part of a consensus conference, we ask you to comment on the proposed recommendations and assess their feasibility. The recommendations will be edited on the basis of your feedback in order to improve the feasibility of the guideline in clinical practice. By taking part in this survey, you are helping to improve the guideline!

Information on data protection

The collected research data is used exclusively for the development of the guideline and for scientific purposes. The research data is anonymous so that no personal reference can be made. It is not possible to draw conclusions about the study participants. The research data only includes information that you provide to us directly via the fields in the online survey. This data is only accessible to individuals who are involved in the analysis.

The results of this study may be published for teaching and research purposes (articles in scientific journals). This will be done anonymously. In order to ensure anonymity, we ask you to not provide any data in the free text responses that would make you clearly recognisable as a person (identifying data such as name or e-mail address).

Information on participation

Participation in this study is voluntary and is aimed at sports physicians and physicians with the additional qualification of

'sports medicine'. Non-participation will not affect you negatively in any way. You can cancel the survey before submitting the questionnaire without giving a reason. In this case, your details will not be taken into account.

Please send any queries to the following e-mail address:

leitlinien-ifom@uni-wh.de

This survey contains 40 questions.

Personal questions

In which year were you born?

Please insert your answer here:

This and all other information is voluntary.

Which gender do you identify with?

Please select the points that apply and write a comment on them:

- [ ] male
- [ ] female
- [ ] other
- [ ] own information
- [ ] no information

Please select only one of the following answers and enter your own information in the field provided, if necessary:

Please enter your medical speciality:

Please select the points that apply and write a comment on them:

- [ ] General medicine
- [ ] Internal medicine
- [ ] Cardiology
- [ ] Surgery
- [ ] Orthopaedics/Traumatology
- [ ] Paediatrics
- [ ] other, namely
- [ ] no information

Which sector do you work in?

Please select only one of the following answers:

- Inpatient
- Outpatient
- No information

In which setting are you active?

Please select only one of the following answers:

- private medical practice
- hospital –based medical practice
- No information

What is your employment relationship?

Please select only one of the following answers:

- Self-employed
- Employed
- No information

Where is your focus of activity?

Please select only one of the following answers:

- University
- Non-university
- No information

Do you have the additional qualification ‘Sports Medicine’“?

Please select only one of the following answers:

- Yes
- no
- No information

How often do you carry out sports preparticipation evaluations for squad athletes?

Please select the points that apply and write a comment on them:

☐ At least 1 time per week

☐ At least 1 time per month

☐ Less often, namely

☐ Never

☐ No information

Please select only one of the following answers and, if necessary, enter your own details in the field provided:

How often do you carry out sports preparticipation evaluations for amateur athletes?

Please select the points that apply and write a comment on them:

☐ At least 1 time per week

☐ At least 1 time per month

☐ Less often, namely

☐ Never

☐ No information

Please select only one of the following answers and, if necessary, enter your own details in the field provided:

Do you know and use the **S1-guideline** „Vorsorgeuntersuchung im Sport“ of the DGSP?

Please select the points that apply and write a comment on them:

☐ Yes, I know the guideline and use it regularly

☐ Yes, I know the guideline, but I rarely/never use it

☐ No, I do not know the guideline

☐ I tend to follow other guidance:

☐ No information

Your opinion on the recommendations

The S2k guideline on sports preparticipation evaluations refers to supposedly healthy adults, with or without disabilities, who want to take part in sport or (re)start doing so. The following recommendations were developed by experts from various professional associations and are currently in the proposal stage.

In this part of the survey, we would like to give you the opportunity to give your opinion.

What aspects could be taken into account in the comments, for example?

1. Is the recommendation stated clearly?
2. Does the recommendation leave room for personal particularities, e.g. a physical limitation?
3. Do you find the recommendation acceptable or do you have concerns about it? If so, what are they?
4. In your opinion, can the recommendation be implemented in everyday clinical practice? If not, why not?
5. Do you miss anything? Would you like to add anything?

In some cases, two variants are suggested for a recommendation. Here you have the option of indicating your preference in the comments field, if necessary with reasons.

The results are then processed and passed on to the experts for revision of the recommendations.

1 Alternative A:

Offer a sports preparticipation evaluation to adults who want to take up or return to sport.

Please insert your answer here:

Please use the field for comments or suggestions for changes to the recommendation.

1 Alternative B:

Offer a sports preparticipation evaluation to adults who do sport.

Please insert your answer here:

Please use the field for comments or suggestions for changes to the recommendation.

2 Alternative A:

Use the relevant guidelines for people with chronic diseases.

Please insert your answer here:

Please use the field for comments or suggestions for changes to the recommendation.

2 Alternative B:

Use the relevant guidelines for people with diseases.

Please insert your answer here:

Please use the field for comments or suggestions for changes to the recommendation.

3:

A sports preparticipation evaluation should be conducted when starting a more intensive sports and exercise programme.

Please insert your answer here:

Please use the field for comments or suggestions for changes to the recommendation.

4 Alternative A:

A sports preparticipation evaluation should be repeated regularly

Please insert your answer here:

Please use the field for comments or suggestions for changes to the recommendation.

4 Alternative B:

A sports preparticipation evaluation should be repeated regularly at intervals of 1-3 years.

Please insert your answer here:

Please use the field for comments or suggestions for changes to the recommendation.

5:

Also use the results of the sports preparticipation evaluation to derive individualised exercise and training recommendations.

Please insert your answer here:

6:

Evidence-based communication strategies should be used to convey exercise recommendations.

Please insert your answer here:

Please use the field for comments or suggestions for changes to the recommendation.

7 Alternative A:

A sports preparticipation evaluation should be carried out by a specialised doctor, preferably with certification in sports medicine.

Please insert your answer here:

Please use the field for comments or suggestions for changes to the recommendation.

7 Alternative B:

A sports preparticipation evaluation should be carried out by a specialised doctor with certification in sports medicine.

Please insert your answer here:

Please use the field for comments or suggestions for changes to the recommendation.

8:

A standardised medical history form should be used as part of the sports preparticipation evaluation. This should include the following items:

- Personal and family medical history
- Individual risk profile
- Participation in ealier check-ups and their results
- Information on surgical procedures and injuries
- Nutritional history
- Gynaecological history
- Vaccination status

Please insert your answer here:

Please use the field for comments or suggestions for changes to the recommendation.

9:

A validated score should be used to assess the cardiovascular risk.

Please insert your answer here:

Please use the field for comments or suggestions for changes to the recommendation.

10:

A sports history should include questions about:

- The frequency, intensity, duration and types of sport
- Previous experience with sport
- Complains at rest and during exercise
- Individual sport-related risk profile and
- Any aids or restrictions (e.g. visual aids)

Please insert your answer here:

Please use the field for comments or suggestions for changes to the recommendation.

11:

Obtain a whole-body assessment as part of the sports preparticipation evaluation:

- Collection of anthropometric data (height, weight, abdominal girth, calculation of body mass index)
- Pulse and blood pressure measurement at rest
- Examination of heart and lungs
- Assessment of vascular status
- Abdominal examination
- Examination of the musculoskeletal system including
  a) mobility and stability of the large joints
  b) mobility and tapping pain of the spine
  c) muscle status / function, tone, etc.
- Examination of the nervous system (gait pattern, reflex status, sensitivity, cranial nerve status if applicable)

Examination of the sensory organs (incl. visual acuity assessment with eye charts)

- Inspection of the skin
- Inspection of the oral cavity, recording of dental status
- Assessment of lymph node status

Please insert your answer here:

12:

Consider measuring muscle strength as part of the sports preparticipation evaluation.

Please insert your answer here:

Please use the field for comments or suggestions for changes to the recommendation.

13:

A full blood count, pre-prandial blood glucose concentration and lipid status, liver/kidney values, electrolyte concentration and urine status can also be determined if necessary.

Please insert your answer here:

Please use the field for comments or suggestions for changes to the recommendation.

14:

Other parameters, e.g. inflammatory markers, renal retention values, should be determined if there is a corresponding clinical suspicion.

Please insert your answer here:

Please use the field for comments or suggestions for changes to the recommendation.

15:

A 12-lead resting ECG should be recorded as part of the sports preparticipation evaluation.

Please insert your answer here:

Please use the field for comments or suggestions for changes to the recommendation.

16:

Do not routinely perform echocardiography as part of the sports preparticipation evaluation.

Please insert your answer here:

Please use the field for comments or suggestions for changes to the recommendation.

17:

An exercise ECG should be recorded as part of the sports preparticipation evaluation if there is an increased cardiovascular risk and/or exercise-induced symptoms.

Please insert your answer here:

Please use the field for comments or suggestions for changes to the recommendation.

18:

Consider using the results of the exercise test to determine cardiorespiratory fitness and to derive training recommendations.

Please insert your answer here:

Please use the field for comments or suggestions for changes to the recommendation.

19:

Spiroergometry can be part of the sports preparticipation evaluation to determine cardiorespiratory fitness and for training recommendations or monitoring.

Please insert your answer here:

Please use the field for comments or suggestions for changes to the recommendation.

20:

Carry out additional laboratory and instrumental examinations only in individually justified cases.

Please insert your answer here:

Please use the field for comments or suggestions for changes to the recommendation.

21:

Swollen joints, pain, relevant movement restrictions or instability of large joints as well as motion pain or pain on percussion of the spine with or without neurological deficits should be referred for specialist evaluation (specialist in orthopaedics/trauma surgery or physical and rehabilitative medicine.

Please insert your answer here:

Please use the field for comments or suggestions for changes to the recommendation.

22:

People with artificial joints or a history of serious injury (e.g. vertebral body fractures, recurrent joint injuries) should be monitored regularly by a specialist.

Please insert your answer here:

Please use the field for comments or suggestions for changes to the recommendation.

23:

Risk to self and others and possible aggravation of previous injuries should be considered when assessing sports clearance.

Please insert your answer here:

Please use the field for comments or suggestions for changes to the recommendation.

Machbarkeit der sportmedizinischen Vorsorgeuntersuchung

Please indicate to what extent you agree with the following statements:

Please select an answer for each item:

	disagree	partially disagree	undecided	partially agree	agree
**I would offer PPEs or am already doing so.**	○	○	○	○	○
**I feel qualified to carry out the internal medicine part of a PPE.**	○	○	○	○	○
**I feel qualified to carry out the orthopaedic part of a PPE.**	○	○	○	○	○
**Time and effort involved in PPEs would be feasible in my practice or clinic.**	○	○	○	○	○
**I understand why PPEs would be useful for my patients.**	○	○	○	○	○
**PPEs would help me to make correct diagnoses.**	○	○	○	○	○
**PPEs would help me advise patients on the correct form, amount and intensity of physical activity.**	○	○	○	○	○
**I could only offer PPEs if they are reimbursed by statutory or private health insurance.**	○	○	○	○	○
**I would have concerns or reservations about offering PPEs.**	○	○	○	○	○

I have comments on the sports preparticipation evaluation:

Please insert your answer here:

18.10.2023 – 06:00
Send in your survey.
Thank you for participating.
